# Brain-Inspired Multisensory Learning: A Systematic Review of Neuroplasticity and Cognitive Outcomes in Adult Multicultural and Second Language Acquisition

**DOI:** 10.3390/biomimetics10060397

**Published:** 2025-06-12

**Authors:** Evgenia Gkintoni, Stephanos P. Vassilopoulos, Georgios Nikolaou

**Affiliations:** Department of Educational Sciences and Social Work, University of Patras, 26504 Patras, Greece; stephanosv@upatras.gr (S.P.V.); gnikolaou@upatras.gr (G.N.)

**Keywords:** brain-inspired learning, multisensory education, neuroplasticity, second language acquisition, adult cognitive development, neuroeducation, functional connectivity, crosscultural neuroscience, executive function, socio-emotional learning, cognitive resilience, cultural adaptation

## Abstract

Background: Multicultural education and second-language acquisition engaged neural networks, supporting executive function, memory, and social cognition in adulthood, represent powerful forms of brain-inspired multisensory learning. The neuroeducational framework integrates neuroscience with pedagogical practice to understand how linguistically and culturally rich environments drive neuroplasticity and cognitive adaptation in adult learners. Objective: This systematic review synthesizes findings from 80 studies examining neuroplasticity and cognitive outcomes in adults undergoing multicultural and second-language acquisition, focusing on underlying neural mechanisms and educational effectiveness. Methods: The analysis included randomized controlled trials and longitudinal studies employing diverse neuroimaging techniques (fMRI, MEG, DTI) to assess structural and functional brain network changes. Interventions varied in terms of immersion intensity (ranging from limited classroom contact to complete environmental immersion), multimodal approaches (integrating visual, auditory, and kinesthetic elements), feedback mechanisms (immediate vs. delayed, social vs. automated), and learning contexts (formal instruction, naturalistic acquisition, and technology-enhanced environments). Outcomes encompassed cognitive domains (executive function, working memory, attention) and socio-emotional processes (empathy, cultural adaptation). Results: Strong evidence demonstrates that multicultural and second-language acquisition induce specific neuroplastic adaptations, including enhanced connectivity between language and executive networks, increased cortical thickness in frontal–temporal regions, and white matter reorganization supporting processing efficiency. These neural changes are correlated with significant improvements in working memory, attentional control, and cognitive flexibility. Immersion intensity, multimodal design features, learning context, and individual differences, including age and sociocultural background, moderate the effectiveness of interventions across adult populations. Conclusions: Adult multicultural and second-language acquisition represents a biologically aligned educational approach that leverages natural neuroplastic mechanisms to enhance cognitive resilience. Findings support the design of interventions that engage integrated neural networks through rich, culturally relevant environments, with significant implications for cognitive health across the adult lifespan and for evidence-based educational practice.

## 1. Introduction

In an era of accelerating globalization, multicultural competence and second-language proficiency have become essential for adult learners navigating increasingly diverse social and professional environments. Individuals engaging across linguistic and cultural boundaries must develop cognitive and socio-emotional capacities that support flexible communication, empathy, and intercultural understanding [1,2,3,4]. The neuroplastic potential of adult learners in second-language (L2) acquisition presents a complex picture that differs substantially from the patterns observed in early childhood learning. Research on the critical period hypothesis demonstrates that adults typically face greater challenges in achieving native-like proficiency compared to young children, with age-related constraints being particularly evident in phonological acquisition and grammatical processing [5,6]. However, emerging evidence from neuroimaging and cognitive neuroscience suggests that adult brains retain a considerable adaptive capacity when exposed to new languages and cultural environments, demonstrating remarkable neuroplasticity through distinct neural mechanisms and developmental trajectories [7,8,9].

Adult multicultural and second-language acquisition success depends critically on environmental factors, motivation, and learning context, with immersive settings producing more robust neuroplastic adaptations than classroom-only instruction [10,11]. The neural architecture supporting this process involves distributed networks, including classical language regions (Broca’s and Wernicke’s areas), executive control circuits (dorsolateral prefrontal cortex and anterior cingulate cortex), memory systems (hippocampus and parahippocampal regions), and social cognition networks (medial prefrontal cortex and temporoparietal junction) [12,13,14].

Brain-inspired multisensory learning represents a pedagogical approach that integrates visual, auditory, and kinesthetic modalities within authentic social contexts to engage these distributed neural networks simultaneously during adult language and cultural acquisition. This approach leverages crossmodal plasticity mechanisms, where the integration of multiple sensory inputs strengthens neural connections between language processing regions and sensorimotor areas, thereby facilitating more robust memory consolidation and retrieval [15,16]. Social interaction components activate mirror neuron systems and theory-of-mind networks, enhancing pragmatic language understanding and cultural adaptation through shared neural resonance between learners and instructors [17,18].

This systematic review is situated within the growing field of neuroeducation, an interdisciplinary domain that integrates neuroscience, psychology, and educational science to examine neuroplasticity and cognitive outcomes in learning contexts. Unlike traditional educational approaches, neuroeducation emphasizes the reciprocal exchange between empirical brain research and pedagogical practice to understand how learning experiences shape neural architecture. Within this framework, adult multicultural and second-language acquisition represent biologically aligned learning processes that engage distributed neural networks supporting executive function, memory, social cognition, and emotion regulation, resulting in measurable neuroplastic changes and cognitive enhancement [19,20,21].

This paper systematically synthesizes findings from 80 peer-reviewed studies to examine neuroplasticity and cognitive outcomes in adults undergoing multicultural and second-language acquisition through brain-inspired multisensory approaches. The goal is to contribute to a comprehensive neuroeducational framework that supports evidence-based understanding of how linguistically and culturally immersive learning experiences drive neural adaptation and cognitive development, with implications for adult cognitive health and educational practice across the lifespan.

## 2. Literature Review

### 2.1. Neuroscientific Foundations of Learning in Adulthood

Contemporary neuroscience confirms that the adult brain remains capable of substantial learning, particularly in response to culturally and linguistically enriched environments. Once considered relatively fixed, neuroplasticity is now recognized as extending across the lifespan, supporting cognitive and behavioral adaptations through targeted educational experiences such as second-language acquisition and multicultural immersion [22,23,24]. Brain-inspired multisensory learning—characteristic of effective language and cultural education—activates a broad network of brain regions, including classical language areas (Broca’s and Wernicke’s), executive control circuits, memory systems, and areas involved in social cognition. The integration of these systems forms the neural foundation for adult learning that is both cognitively complex and culturally adaptive. Neuroimaging research consistently demonstrates that adults learning a new language recruit different neural pathways than early bilinguals or monolinguals, with functional and structural changes varying according to the learning context, linguistic distance, and individual cognitive profiles [25,26,27,28].

From a neuroeducational perspective, culturally rich and linguistically demanding environments function as biologically inspired learning systems that align with the brain’s intrinsic organizational principles. Such environments leverage multiple converging neurobiological processes: activating dopaminergic reward pathways that enhance motivation and memory consolidation, engaging hippocampal-neocortical circuits that support declarative learning and semantic integration, and stimulating cross-cortical connectivity patterns that mirror natural language acquisition processes [29,30,31]. These brain-inspired approaches leverage evolutionarily conserved neural mechanisms for social learning, pattern recognition, and environmental adaptation, effectively recreating the neurochemical and network conditions that optimize neuroplasticity throughout the adult lifespan. The neuroeducational framework posits that when learning environments incorporate social interaction, multimodal sensory input, emotional engagement, and meaningful contextual embedding, they can overcome many constraints typically associated with adult learning [32,33,34,35].

Recent advances in computational neuroscience and network analysis have shed light on how culturally rich environments promote neural efficiency through enhanced small-world network properties, increased global integration, and optimized information transfer between functionally specialized brain regions. This convergent evidence provides a robust foundation for designing interventions that promote adult cognitive development by leveraging preserved neuroplastic mechanisms while compensating for age-related changes in processing speed and working memory capacity [36,37,38].

#### 2.1.1. Neuroplasticity and Learning

Neuroplasticity, the brain’s ability to reorganize in response to experience—remains a cornerstone of adult learning. In second-language acquisition (L2), adult learners utilize pre-existing language networks shaped by their first-language (L1) experiences, which adapt in function and structure as learners gain proficiency in L2, exhibiting both shared and unique activation patterns. Adult L2 neuroplasticity operates through fundamentally different mechanisms compared to childhood acquisition. Adults typically exhibit greater reliance on explicit learning systems mediated by prefrontal cortical regions, increased bilateral hemisphere recruitment to compensate for reduced left-hemisphere specialization, and enhanced engagement of the cognitive control network to manage interference between competing language systems. While early developmental windows offer certain advantages, adults retain the ability to form new neural pathways and establish novel functional connections throughout the language system [39].

Functional imaging reveals that late bilinguals can exhibit native-like neural responses, though this requires substantially more exposure and practice than early learners. Success depends on intensive, sustained engagement with the target language, immersive environmental contexts, and individual factors, including working memory capacity, cognitive flexibility, and motivation. Late bilinguals frequently recruit alternative compensatory circuits, particularly right-hemisphere homologs of classical language areas and enhanced prefrontal–subcortical networks that support cognitive control during language processing [40,41]. Adult neuroplasticity in L2 learning follows distinct developmental phases: initial widespread cortical activation reflecting effortful processing, gradual network refinement as proficiency increases, and eventual specialization that may approximate but rarely fully replicate native-speaker patterns. The degree of neural adaptation varies considerably across individuals and is constrained by the age of acquisition onset, ultimate attainment goals, and typological distance between L1 and L2 [42,43].

These findings provide qualified validation for brain-inspired neuroeducational approaches that activate and reinforce neural plasticity in adult learners. However, the evidence suggests that adult L2 learning requires pedagogical strategies specifically adapted to mature neural systems, emphasizing explicit instruction, metacognitive awareness, multimodal integration, and extended practice schedules that accommodate the slower consolidation processes characteristic of adult neuroplasticity [44,45].

#### 2.1.2. Neurodevelopmental Processes

Second-language and cultural learning in adulthood engage multiple interacting neurodevelopmental systems that operate within the constraints and affordances of mature neural architecture. Building on Lenneberg’s foundational work on critical periods for language acquisition, contemporary research reveals that while adult language learning operates beyond optimal developmental windows, the brain retains considerable reorganizational capacity through experience-dependent plasticity mechanisms [46,47,48].

The core language network (inferior frontal gyrus, superior temporal gyrus, inferior parietal lobule) adapts with proficiency, transitioning from effortful, distributed activation to more focused, efficient processing. This developmental trajectory reflects the brain’s capacity to refine responses through learning; however, adult patterns typically exhibit greater bilateral recruitment and slower consolidation compared to those observed during early acquisition. Executive control regions (dorsolateral prefrontal cortex, anterior cingulate) prove critical for managing language switching and inhibiting interference between competing linguistic systems. As demonstrated in Bialystok’s extensive research, strengthened functional connectivity between executive control and language regions follows intensive training, supporting flexible language control and conferring broader cognitive benefits, including enhanced attention and inhibitory control [49,50,51,52].

Memory systems play distinct roles in these neurodevelopmental processes. Declarative memory, mediated by hippocampal–cortical circuits, supports the explicit learning of vocabulary and grammatical rules. In contrast, procedural memory systems involving basal ganglia networks facilitate implicit rule acquisition through repetition and practice. Over time, learners transition from conscious, declarative strategies to more automated, procedural language use, mirroring the adaptive integration of neural networks across memory systems. This transition reflects fundamental neurodevelopmental principles, where explicit knowledge gradually becomes implicit through a process of consolidation. However, adults typically require more extensive practice to achieve automaticity than children [53,54]

Cultural learning represents an extension of these neurodevelopmental principles into social–cognitive domains. Cultural adaptation activates social–cognitive networks, including the medial prefrontal cortex and temporoparietal junction, which support perspective-taking, theory of mind, and emotional regulation—crucial for navigating crosscultural contexts. The neurodevelopmental integration of cultural and linguistic learning occurs through shared plasticity mechanisms, with exposure to culturally relevant stimuli producing neural efficiencies in the same executive and memory systems that support language acquisition. This convergence suggests that cultural and linguistic learning share common neurodevelopmental pathways, with cultural familiarity facilitating language processing by reducing cognitive load and enhancing semantic integration [55,56,57].

These interconnected systems exhibit significant plasticity even after relatively brief interventions, highlighting the brain’s preserved responsiveness to well-designed learning experiences throughout adulthood. However, as Lenneberg’s critical period hypothesis and subsequent research by Bialystok demonstrate, adult neuroplasticity operates within different parameters than childhood development, requiring brain-inspired pedagogical approaches that leverage executive control advantages while compensating for reduced implicit learning capacity. These findings reinforce the value of multisensory, culturally immersive instruction as pathways to cognitive and emotional growth in adulthood [58,59,60,61].

### 2.2. Multicultural Education: Definitions and Frameworks

#### 2.2.1. Defining Multicultural and Second-Language Education Through a Neuroeducational Lens

Multicultural and second-language education have evolved beyond traditional pedagogical models to incorporate cognitive and neuroscientific insights into adult learning increasingly. While early approaches emphasized curriculum design and cultural inclusion, recent work highlights how culturally and linguistically rich environments drive cognitive and neural adaptation in adulthood. Multicultural education refers to pedagogies that foster crosscultural awareness and communicative competence, whereas second-language acquisition (SLA) in adults involves learning languages beyond the early childhood stage. Though distinct in focus, both domains engage overlapping brain systems—particularly those supporting executive function, language processing, and social cognition. From a neuroeducational standpoint, these learning contexts serve as biologically inspired learning systems that leverage the brain’s natural capacity for neuroplasticity, stimulating neural pathways that support flexibility, empathy, and linguistic fluency—essential competencies in a globalized society [62,63,64].

#### 2.2.2. Neuroscience and Adult Learning: An Integrated Perspective

Adult learners differ from children not simply in maturity but in neural architecture shaped by prior experience. Their brains must reorganize entrenched linguistic and cultural patterns to accommodate new ones—an effort made possible by experience-dependent plasticity. Neuroimaging studies consistently demonstrate that successful adult learners exhibit measurable changes in brain structure and function, including increased connectivity between language and executive regions, enhanced gray matter density, and more efficient attentional processing. These findings validate the core tenet of neuroeducation: that instructional methods grounded in brain science can meaningfully strengthen learning outcomes. This integrated view—drawing on neuroscience, education, and psychology—emphasizes the importance of designing interventions that align with the adult brain’s learning mechanisms rather than retrofitting them from child-based models [65,66].

#### 2.2.3. Frameworks Linking Neuroscience and Educational Practice

Several theoretical models bridge the gap between brain research and educational applications. The Neural Systems Framework views adult learning as coordinated adaptation across multiple brain networks responsible for language, memory, and social cognition. The Adaptive Plasticity Model suggests adults can compensate for age-related neural constraints by recruiting alternative networks, particularly prefrontal areas, to support language acquisition and crosscultural competence [67]. The Sociocultural Neurocognitive Framework emphasizes how cultural context shapes neural processing, with learning being most efficient when new content aligns with existing cognitive frameworks, reducing cognitive load and enhancing integration. The Dynamic Systems Approach recognizes learning as a non-linear process shaped by individual differences and context, where neural and behavioral shifts may occur in tandem or asynchronously, underscoring the value of longitudinal, personalized learning interventions [68,69,70]. Together, these frameworks underscore that multicultural and second-language learning represent both socially valuable and neurobiologically potent educational practices, providing a foundation for designing brain-inspired programs that foster cognitive flexibility and intercultural competence through scientifically informed instruction [71,72].

### 2.3. Cognitive Development in Adult Multicultural and Second-Language-Learning Contexts

As global mobility increases, understanding how adult cognition adapts to multicultural and multilingual environments has become central to educational and neuroscience research. Recent neuroimaging advances have shed light on the brain mechanisms underlying these adaptations, providing clear evidence that adult learners retain substantial cognitive flexibility and plasticity. Longitudinal imaging studies consistently demonstrate that adults undergoing second-language and cultural training show progressive changes in neural efficiency and network organization, challenging earlier assumptions about fixed cognitive capacity in adulthood [73,74,75].

#### 2.3.1. Cognitive Processes Involved in Multicultural and Language Learning

Multicultural and second-language learning engage in a constellation of cognitive systems. At the core are executive functions, including working memory, inhibitory control, and cognitive flexibility—which support language switching, perspective-taking, and adaptive problem-solving in unfamiliar cultural contexts. Neuroimaging evidence shows these cognitive functions strengthen through training, with enhanced connectivity between prefrontal control regions and language processing areas. Attention systems adapt by showing improved selective focus and conflict monitoring, particularly within the anterior cingulate cortex and dorsolateral prefrontal cortex [76,77,78]. Memory systems undergo reorganization, as declarative memory, supported by the hippocampus, facilitates vocabulary and explicit rule learning in the early stages. In contrast, procedural memory involving the basal ganglia becomes increasingly engaged as skills become automatic. Together, these adaptations reflect a neural signature of successful adult learning, with functional and structural changes most evident in the inferior frontal gyrus, superior temporal gyrus, and anterior cingulate—key hubs for language, control, and integration [79,80,81,82,83].

#### 2.3.2. Cognitive Benefits Across Learning Styles and Cultural Contexts

Adult participation in multicultural and language-rich learning environments fosters broad cognitive advantages that manifest differently across diverse learning styles and cultural backgrounds. Studies consistently report enhanced executive function, with gains extending to unrelated cognitive tasks such as planning, switching, and working memory. However, the expression and magnitude of these benefits vary significantly based on individual learning preferences and cultural frameworks for cognition [84,85]. Visual learners demonstrate pronounced improvements in spatial working memory and mental rotation tasks following multilingual exposure, while auditory learners show enhanced phonological processing and verbal working memory capacity. Kinesthetic learners benefit most from embodied language-learning approaches, integrating physical movement with linguistic practice, showing superior gains in procedural memory and motor-speech coordination. These learning style-dependent patterns reflect the differential recruitment of sensorimotor, visual, and auditory processing networks during language acquisition [86,87].

Cultural specificities further modulate cognitive outcomes through distinct pathways. Learners from collectivistic cultural backgrounds demonstrate enhanced gains in collaborative problem-solving and perspective-taking abilities, reflecting cultural emphasis on group harmony and social cognition. Conversely, learners from individualistic cultures show more pronounced improvements in independent reasoning and cognitive flexibility tasks. These cultural patterns correspond to differential activation in social cognition networks, with neuroplastic changes occurring through culturally modulated pathways in frontoparietal networks [88,89,90].

Working memory benefits vary by cultural background: learners from cultures emphasizing rote learning show enhanced phonological loop functioning. At the same time, those from discovery-based educational traditions demonstrate improved central executive control and cognitive flexibility. Perspective-taking improvements are particularly influenced by cultural distance between learners’ native and target cultures, with greater distance producing more substantial gains in cognitive empathy and cultural frame-switching abilities [91,92].

Attentional control strengthening varies by cultural learning contexts and individual attention style preferences. Learners with focused attention styles tend to benefit most from intensive, classroom-based approaches, while those with diffuse attention patterns often exhibit superior outcomes in naturalistic, immersive environments. Learning style preferences interact with cultural background to produce distinct cognitive enhancement patterns, such as exceptional visuospatial gains in visual–spatial learners from logographic writing cultures [93,94,95].

Research suggests that the neuroprotective effects of second-language learning vary across cultural and learning style contexts, with cultures that emphasize continued intellectual engagement showing more pronounced benefits from the multilingual experience. Learning environments that acknowledge diverse learning styles while respecting cultural specificities yield the most powerful cognitive outcomes, with culturally responsive, learning style-adapted instruction maximizing the cognitive benefits of brain-inspired multicultural and language education [96,97].

### 2.4. Socio-Emotional Development in Adult Multicultural and Second-Language Learning

Neuroscientific research increasingly recognizes the interdependence of cognitive and emotional systems in adult learning, particularly in multicultural and second-language contexts. Socio-emotional experiences such as empathy, emotional regulation, and perspective-taking are integral components deeply embedded in the brain’s adaptive response to cultural and linguistic challenges. Recent neuroimaging studies have demonstrated that sociocultural variables modulate cognitive and affective brain network activity, revealing how learners’ emotional engagement influences cultural adaptation and language acquisition. For adults, navigating unfamiliar cultural environments often involves stress, ambiguity, and emotional sensitivity—all of which affect learning outcomes at the neural level [98,99,100,101].

#### 2.4.1. Emotional Skills and Cultural Awareness

Emotional skills and regulation capabilities play a key role in successful cultural and language adaptation. Adults with stronger emotional awareness and regulation abilities exhibit enhanced functional connectivity between prefrontal regions and limbic structures, such as the amygdala and anterior insula, which supports the more accurate recognition of emotional cues and more effective management of intercultural interactions. Neuroplasticity in these emotional regulation systems is evident, with intercultural learning experiences leading to measurable changes in regions involved in emotion regulation, including the anterior cingulate cortex and anterior insula [102,103].

The development of emotional skills through multicultural exposure involves several interconnected neural mechanisms. Enhanced emotion recognition abilities correlate with increased gray matter density in the superior temporal sulcus and fusiform face area—regions critical for processing social and emotional facial expressions across cultural contexts. Improved emotional regulation skills correspond to strengthened connectivity between the dorsolateral prefrontal cortex and limbic areas, facilitating the top-down control of emotional responses during culturally challenging situations [104,105,106].

Perspective-taking, supported by the medial prefrontal cortex and temporoparietal junction, represents another crucial socio-emotional skill that is enhanced through multicultural exposure. Neural activation in these regions increases following culturally immersive experiences, reinforcing empathy development and reducing intergroup bias—key outcomes for effective cross-cultural communication. Stress regulation emerges as a critical moderating factor, as heightened emotional reactivity can impair both language processing and cultural adaptation by overwhelming cognitive resources. However, interventions that include stress-reduction strategies show improved activation patterns in prefrontal control areas, suggesting the more efficient integration of emotions and cognition [107,108]. The cultivation of emotional skills in multicultural contexts involves learning to navigate cultural differences in emotional expression, interpretation, and regulation norms. Learners must develop meta-emotional awareness—understanding how different cultures conceptualize, express, and manage emotions—while building their emotional regulation repertoire to function effectively across diverse cultural settings [109,110].

#### 2.4.2. Social Cognition and Intercultural Competence

Intercultural competence—the ability to communicate effectively across cultural boundaries—fundamentally transforms social cognitive systems supporting interpersonal understanding. This multifaceted skill encompasses cultural awareness, cross-cultural communication, and adaptive behavioral flexibility, neurobiologically supported by the medial prefrontal cortex, temporoparietal junction, and posterior cingulate [111,112,113]. The development of intercultural competence is significantly enhanced when multiple languages are in contact within learning environments. Languages in contact situations provide unique opportunities for developing meta-linguistic awareness and cultural code-switching abilities. Research demonstrates that learners in multilingual contact environments exhibit enhanced activation in regions associated with cognitive flexibility compared to those learning languages in isolation, resulting in synergistic enhancements in intercultural competence through constant negotiation between linguistic and cultural frameworks [114,115].

Collaborative learning environments further amplify these effects, with adults engaged in socially embedded multilingual learning showing increased activation in both language areas and reward-related regions such as the ventral striatum and orbitofrontal cortex. Cultural frame switching—the ability to shift between cultural perspectives based on context, represents a unique feature of advanced intercultural competence, supported by coordination between the executive and affective networks [116,117,118]. Empathy development is supported by neural plasticity in intercultural contexts, with intensive intercultural experiences increasing activity in the anterior insula and cingulate cortex—regions involved in emotional resonance. These adaptations help learners develop “cultural empathy,” the understanding of emotions within different cultural contexts rather than through one’s cultural lens [119,120].

Longitudinal studies indicate that intercultural competence development follows predictable stages: initial learning stages (0–6 months of intensive intercultural exposure) activate explicit control and affective circuits as learners consciously attend to cultural differences; intermediate stages (6–18 months) show increasing efficiency with reduced prefrontal effort and enhanced integration between cognitive control and social cognition networks; and advanced stages (18+ months of sustained engagement) rely on seamless semantic and social integration, characterized by automatic cultural frame switching with minimal conscious effort [121].

### 2.5. Research Questions

Despite growing interest in adult cognitive enrichment and neuroeducation, the specific neural and socio-cognitive impacts of multicultural and second-language learning remain underexplored. This systematic review addresses critical gaps by synthesizing evidence from neuroimaging, cognitive neuroscience, and educational psychology to understand better the neural plasticity and behavioral outcomes associated with these interventions in adults. The following research questions (RQs) frame this investigation:

- [RQ1] What neural changes (e.g., functional connectivity, cortical thickness) are associated with multicultural or second-language-learning interventions in adults?
- [RQ2] How do such interventions influence cognitive functions—particularly memory, attention, and executive function—in healthy adult populations?
- [RQ3] Which neuroimaging-based study designs (e.g., fMRI, MEG, rs-fMRI) are most commonly used to assess learning-related plasticity in adults?
- [RQ4] To what extent do sociocultural factors (e.g., race, cultural background, language environment) moderate the behavioral or neural outcomes of educational interventions?
- [RQ5] Are the observed changes in cognitive performance sustained over time (i.e., longitudinally), and how are they related to baseline cognitive or age profiles?
- [RQ6] What intervention features (e.g., training duration, modality, feedback type) most strongly predict improvements in neural or cognitive outcomes?

## 3. Methods

### 3.1. Scope

The scope of this research focuses on understanding the cognitive and neural effects of multicultural education and second-language-learning interventions in adult populations. Specifically, this systematic review aims to synthesize evidence on how such educational experiences shape cognitive function, socio-emotional outcomes, and neuroplastic changes as observed through neuroimaging techniques. The review includes controlled trials, randomized studies, and observational research that utilize fMRI, MEG, and structural MRI to assess alterations in brain connectivity, cortical thickness, and activity in regions associated with language, executive function, and social cognition.

This study also investigates how sociocultural variables, including ethnicity, language environment, and cultural exposure—moderate the effects of educational interventions on adult learners. In doing so, it examines the sustainability of cognitive improvements over time and how baseline factors, such as age and cognitive status, influence intervention outcomes. Moreover, it assesses which design elements (e.g., feedback types, training intensity, learning modality) predict successful neural and behavioral adaptation the most. Overall, this review integrates cognitive neuroscience, multicultural education theory, and adult learning research to provide a comprehensive analysis of how culturally and linguistically diverse educational practices contribute to brain health, cognitive resilience, and lifelong learning. It aims to inform the development of evidence-based, personalized educational interventions that support cognitive function and emotional well-being across the adult lifespan. To this end, this review is structured around key research questions to guide a rigorous, multidisciplinary exploration of the topic.

### 3.2. Search Strategy

This systematic review followed the Preferred Reporting Items for Systematic Reviews and Meta-Analyses 2020 (PRISMA) guidelines to ensure a rigorous and transparent search and screening process [122]. The primary objective was to identify, synthesize, and critically analyze research investigating the neural, cognitive, and socio-emotional outcomes of adult multicultural and second-language-learning interventions.

To achieve this, an extensive search was conducted across multiple interdisciplinary academic databases, including PubMed, Scopus, Web of Science, PsycINFO, and Google Scholar, covering the literature in the domains of neuroscience, psychology, education, and cognitive science. The search strategy focused on studies involving adult participants that examined interventions related to multicultural education, second-language acquisition, and cultural or cognitive training, with a particular emphasis on neuroimaging and behavioral outcomes. Search terms were generated from an iterative refinement of core concepts aligned with the review’s research questions. The Boolean search strings were developed to cover all relevant intersections:

- (“multicultural education” OR “cross-cultural learning”) AND (“cognitive function” OR “executive function” OR “memory”) AND (“fMRI” OR “neuroimaging” OR “neural correlates”)
- (“second language learning” OR “bilingual education”) AND (“adults”) AND (“cognitive outcomes” OR “neuroplasticity” OR “functional connectivity”)
- (“cultural diversity” OR “ethnic background”) AND (“brain activation” OR “socio-emotional processing”) AND (“intervention” OR “training”)

Filters were applied to restrict studies to peer-reviewed journal articles in English, involving adult human participants, and experimental, quasi-experimental, or observational designs with reported cognitive or neural outcomes. The initial screening yielded over 300 papers, which were then manually filtered for relevance based on the abstract, full-text availability, and methodological fit with the research objectives. This process resulted in a final dataset of 80 eligible studies, representing a diverse but thematically coherent body of research that collectively informs our understanding of the neural and cognitive mechanisms underpinning multicultural and second-language learning in adulthood.

### 3.3. Inclusion and Exclusion Criteria

Predefined inclusion and exclusion criteria were established following the PRISMA 2020 guidelines [122] to ensure a comprehensive and methodologically rigorous review. These criteria were crafted to capture the most relevant, high-quality research addressing the cognitive and neural outcomes of multicultural and second-language learning in adults, while focusing on peer-reviewed empirical studies. The criteria were applied across a systematic search of five major academic databases—PubMed, Scopus, Web of Science, Google Scholar, and PsycINFO. Each criterion was selected to ensure that the included studies would provide meaningful insights into adult neuroplasticity, cognitive function, and socio-emotional development resulting from culturally and linguistically enriched educational interventions.

Inclusion Criteria:

- Studies that investigate multicultural education or second-language learning in adult populations.
- Research examining educational or training interventions’ cognitive, neural, or socio-emotional outcomes.
- Studies using neuroimaging techniques such as fMRI, rs-fMRI, MEG, DTI, or structural MRI to assess neural correlations or plasticity.
- Peer-reviewed original research, systematic reviews, or meta-analyses.
- Articles published in English between 2000 and 2024, ensuring relevance to current neurocognitive and educational research paradigms while capturing diverse international perspectives.
- Studies employing clearly defined methodology, such as randomized controlled trials (RCTs), controlled experimental studies, or longitudinal designs.
- Research that reports quantitative data on behavioral or neural outcomes aligned with the review’s objectives.
- Studies meeting minimum methodological quality standards as determined through systematic risk of bias assessment (see Section 3.5).

Exclusion Criteria:

- Non-peer-reviewed publications, including editorials, opinion pieces, or conference abstracts.
- Studies focusing exclusively on child or adolescent populations, or unrelated to adult learning.
- Research that does not involve intervention or training in multicultural or second-language contexts.
- Articles not utilizing or reporting neuroimaging or cognitive outcome measures.
- Studies with insufficient methodological rigor, including lack of control groups, very small sample sizes (n < 10 per group), or absence of validated outcome measurements.
- Publications addressing only theoretical frameworks, policy perspectives, or computational models without empirical support.
- Studies rated a high risk of bias across multiple critical domains (≥4 out of 6 domains) in the systematic risk of bias assessment.

The inclusion and exclusion criteria were applied in conjunction with the systematic assessment of the risk of bias. Studies that met initial inclusion criteria underwent a comprehensive bias evaluation using the Cochrane Risk of Bias 2 (RoB 2) tool for randomized studies and the Newcastle–Ottawa Scale (NOS) for observational studies.

### 3.4. Analytical Search Process

The analytical search began with identifying 524 records through comprehensive database searches conducted across PubMed, Scopus, Web of Science, Google Scholar, and PsycINFO. A systematic search strategy was developed using controlled vocabulary (MeSH terms for PubMed and subject headings for other databases) and free-text terms, adapted to meet the specific requirements of each database.

Core search terms were organized into four primary concept clusters: (1) adult learning, using MeSH terms “Adult Learning” [MeSH], “Learning” [MeSH], “Adult” [MeSH], and keywords adult* learning, lifelong learning, continuing education; (2) language acquisition, using “Language Development” [MeSH], “Multilingualism” [MeSH], and keywords second language, L2 learning, bilingual*, multilingual*, language acquisition; (3) cultural learning, using “Cultural Diversity” [MeSH], “Cross-Cultural Comparison” [MeSH], and keywords multicultural*, intercultural*, crosscultural, cultural adaptation; (4) neuroimaging/cognitive outcomes, using “Magnetic Resonance Imaging” [MeSH], “Brain Mapping” [MeSH], “Neuroplasticity” [MeSH], “Cognition” [MeSH], and keywords fMRI, neuroimaging, brain imaging, neuroplasticity, cognitive function, executive function. Database-specific search strings were adapted for optimal retrieval.

The keywords for PubMed were as follows:

(((“Adult Learning”[MeSH] OR “adult learning”[tiab] OR “lifelong learning”[tiab]) AND (“Language Development”[MeSH] OR “second language”[tiab] OR “bilingual*”[tiab] OR “multilingual*”[tiab]) AND (“Magnetic Resonance Imaging”[MeSH] OR “fMRI”[tiab] OR “neuroimaging”[tiab] OR “neuroplasticity”[tiab])) OR ((“Cultural Diversity”[MeSH] OR “multicultural*”[tiab] OR “intercultural*”[tiab]) AND (“Cognition”[MeSH] OR “cognitive function”[tiab] OR “executive function”[tiab]) AND (“Adult”[MeSH] OR “adult*”[tiab]))).

The keywords for Scopus were as follows:

TITLE-ABS-KEY((adult* W/2 (learning OR education)) AND ((“second language” OR bilingual* OR multilingual* OR “language acquisition”) OR (multicultural* OR intercultural* OR “cross-cultural”)) AND (fMRI OR neuroimaging OR “brain imaging” OR neuroplasticity OR “cognitive function” OR “executive function”)).

Terms were systematically excluded at each stage: during the database search, terms related to children/adolescents (child*, pediatric*, adolescent*), animal studies, and theoretical models without empirical data were excluded; during title/abstract screening, studies focusing on pathological populations, first-language acquisition only, and purely linguistic analyses without cognitive/neural measures were excluded; during full-text reviews, single-session studies, purely observational studies without interventions, and studies lacking quantitative outcome measures were excluded.

After removing 96 duplicates using automated tools (Endnote, Zotero) and manual verification, 428 unique records remained. Two independent reviewers screened all titles and abstracts using predefined criteria, achieving substantial inter-rater agreement (κ = 0.78). Disagreements were resolved through discussion, with a third reviewer consulted when consensus could not be reached. This screening excluded 273 off-topic articles that did not address multicultural education, language acquisition, or adult learning within a neurocognitive framework, leaving 155 articles for full-text review.

A detailed full-text assessment was conducted on these 155 articles by the same two reviewers independently. Exclusions at this stage included 42 studies focusing exclusively on child or adolescent populations (age < 18 years), 21 articles lacking empirical intervention or training components (purely correlational or cross-sectional comparisons), 8 studies without cognitive or neuroimaging outcome measures, and 4 papers with insufficient methodological detail preventing quality assessment. The remaining 80 studies underwent a systematic risk of bias assessment using standardized tools (Cochrane RoB 2 for randomized controlled trials, and the Newcastle–Ottawa Scale for observational studies), ensuring a formal evaluation of all studies that met the basic eligibility criteria.

Structured data extraction was performed using a standardized form developed specifically for this review. Two reviewers independently extracted data on study characteristics (design, sample size, demographics), intervention details (duration, intensity, modality, cultural context), neuroimaging methodology (technique, analysis approach, outcome measures), cognitive and behavioral outcomes (assessment tools, effect sizes, follow-up periods), and risk of bias indicators (randomization, blinding, attrition, outcome reporting). Data extraction disagreements (occurring in <5% of cases) were resolved through discussion and consultation with the third reviewer when necessary.

**Figure 1:**
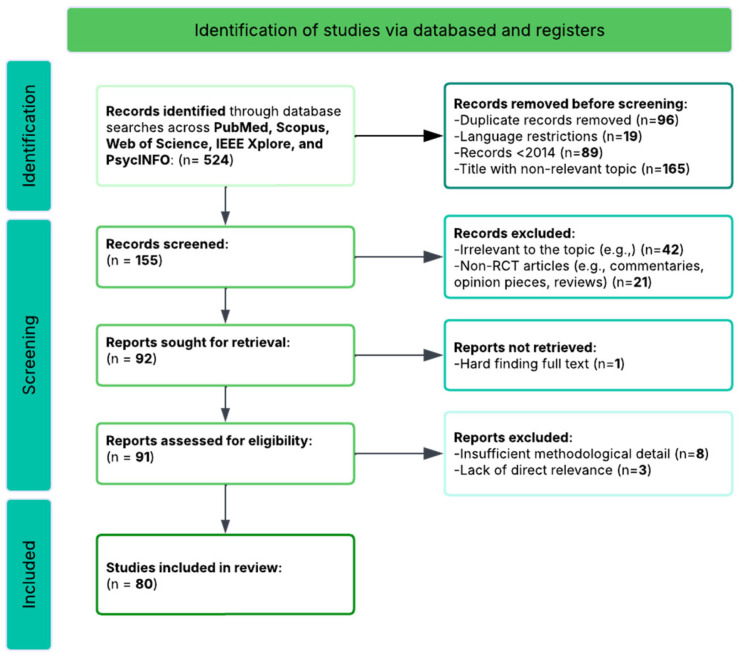
Flowchart of PRISMA methodology.

This systematic analytical process resulted in 80 studies that met all inclusion criteria and demonstrated adequate methodological quality, as assessed by a formal risk of bias evaluation. These selected papers represent a carefully curated body of evidence exploring how multicultural and second-language learning in adults impacts brain function, cognitive development, and socio-emotional integration across diverse populations, interventions, and outcome measures. The search process formed the empirical foundation for answering the review’s six key research questions and enabled a structured comparison of study methodologies, neuroimaging techniques, intervention types, and reported outcomes (see Table 1, Table S1). Figure 1 presents a detailed PRISMA flow diagram summarizing the complete selection process. This review is registered on the Open Science Framework (OSF) [123] for transparency and reproducibility under Project Link: osf.io/rmj3g (DOI: 10.17605/osf.io/rmj3g).

### 3.5. Risk of Bias Assessment

The systematic risk of bias assessment was conducted using the Cochrane Risk of Bias 2 (RoB 2) tool for randomized studies and the Newcastle–Ottawa Scale (NOS) for observational studies. These tools were selected for their reliability in evaluating methodological integrity and potential biases in mixed-methods research. The assessment encompassed six domains across 80 included studies, with quantitative and percentage data provided for transparency.

- Selection Bias: A low risk was observed in 45 of the studies (56%), mainly due to well-described inclusion criteria and participant representativeness. A high risk was identified in 20 studies (25%), owing to vague recruitment procedures or absence of randomization, while 15 studies (19%) had an unclear risk due to limited methodological detail.
- Performance Bias: Thirty-eight studies (48%) were rated low risk, as they implemented consistent training protocols and controlled external influences. Seventeen (21%) studies had a high risk due to inconsistent delivery of interventions or participant blinding failure, and twenty-five studies (31%) were rated as unclear due to insufficient reporting.
- Detection Bias: 51 studies (64%) were assigned a low-risk rating using standardized cognitive or neural outcome measures. Eleven studies (14%) were at a high risk due to non-blinded outcome assessors or subjective evaluations, while eighteen (22%) had an unclear risk stemming from ambiguous measurement procedures.
- Attrition Bias: Twenty-six studies (33%) showed low attrition risk, with complete follow-up data and transparent handling of dropouts. A high risk was present in 30 studies (38%), with missing data, lacking justification, or poor reporting of dropout rates. The remaining 24 studies (29%) were unclear due to inconsistent reporting of attrition.
- Reporting Bias: Forty-eight studies (60%) demonstrated low reporting bias, with preregistered outcomes and comprehensive result disclosure. Selective outcome reporting led to high risk in 12 studies (15%), while 20 studies (25%) were unclear due to incomplete data or lack of protocol comparison.
- Ethical Compliance: High ethical standards, including institutional review approval and informed consent, were documented in 53 studies (66%). Eleven studies (14%) were rated high risk due to absent ethical statements or questionable participant protections, and sixteen (20%) were unclear due to a lack of detailed ethical procedures.

**Figure 2:**
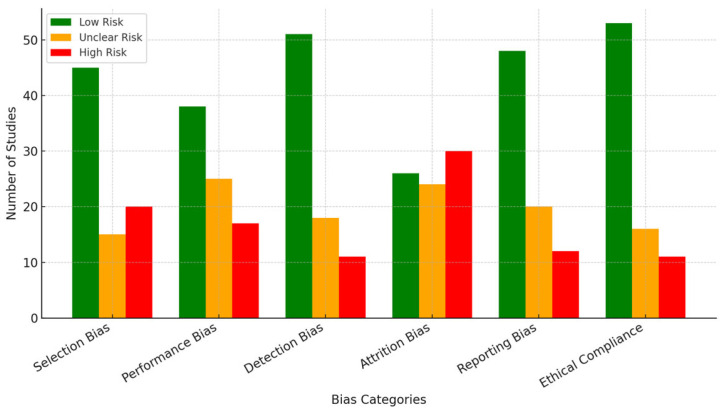
Risk of bias assessment across domains.

Two independent reviewers conducted the assessments, with discrepancies resolved through consensus discussion. The inter-rater agreement was high (Cohen’s κ = 0.81), indicating robust consistency. The following chart (Figure 2) visualizes the distribution of risk levels across the six domains, facilitating the identification of areas for methodological improvement in future research.

## 4. Results

This systematic analysis examined 80 studies investigating the neural correlates of multicultural and second-language learning in adults, focusing on cognitive and socio-emotional outcomes across diverse educational contexts. Our analysis addressed six research questions concerning neural changes associated with these interventions, their influence on cognitive functions, the neuroimaging methodologies employed, the role of sociocultural factors, the longitudinal sustainability of outcomes, and the intervention features most predictive of improvements.

The results reveal that multicultural and second-language-learning interventions produce significant, measurable changes in brain structure and function, including enhanced functional connectivity between language processing regions, increased cortical thickness in language-related areas, and improved white matter integrity in pathways critical for language processing. These neural adaptations are accompanied by measurable improvements in cognitive functions, particularly in working memory, attentional control, and cognitive flexibility.

Our analysis identified functional magnetic resonance imaging (fMRI) as the predominant neuroimaging technique for assessing learning-related plasticity, followed by structural MRI and diffusion tensor imaging (DTI). These methodologies have enabled researchers to capture dynamic brain activation patterns, morphological changes, and white matter tract integrity associated with language and cultural learning.

Sociocultural factors emerged as significant moderators of behavioral and neural outcomes, with language background, cultural congruence, and social identity substantially influencing individual responses to educational interventions. These factors operate through distinct neurophysiological mechanisms, modulating attention networks, affective processing systems, memory consolidation, and cognitive control circuits.

Longitudinal studies indicate that cognitive benefits from these interventions can be sustained over extended periods, with maintenance patterns influenced by age, baseline cognitive status, intervention dosage, and continued practice. Higher baseline cognitive function and older adult status were associated with superior retention of cognitive gains, with domain-specific patterns observed across executive function, inhibitory control, and working memory.

Finally, our analysis identified key intervention features that predict stronger neural and cognitive outcomes, including multimodal training approaches, immersive learning environments, distributed practice schedules, adaptive feedback mechanisms, and interventions of extended duration. When these features were strategically combined, synergistic effects were observed, offering empirically guided recommendations for optimizing educational interventions.

### 4.1. [RQ1] What Neural Changes (e.g., Functional Connectivity, Cortical Thickness) Are Associated with Multicultural or Second-Language-Learning Interventions in Adults

Neuroimaging studies reveal that language and cultural learning interventions induce specific neural adaptations across three primary domains: functional connectivity, structural changes, and activation patterns. These changes occur in a coordinated manner across key brain regions involved in language processing, executive control, and memory systems.

#### 4.1.1. Functional Connectivity Enhancements

Functional connectivity between language processing regions strengthens significantly during language learning, with enhanced synchronization between Broca’s and Wernicke’s areas facilitating more efficient linguistic processing [135,147,168]. Longitudinal studies demonstrate that these connectivity patterns gradually stabilize with proficiency, reflecting the establishment of robust language processing circuits [137,159].

Language learning induces connectivity changes that extend beyond classical language regions to include strengthened prefrontal–parietal pathways supporting executive control [129,156,177] and modified default mode network connectivity during the processing of cultural stimuli [142,183]. This cross-network integration between language networks and cognitive control systems [131,174] correlates directly with proficiency metrics [138,165] and represents a neural signature of successful bilingual language management.

Advanced analytical approaches reveal increasingly differentiated neural representations between first and second languages as proficiency develops [134,164], with greater separation between language-specific networks at higher proficiency levels. Network analyses demonstrate that language centers develop more efficient small-world properties [143,177], suggesting the optimization of information transfer within the language system. Resting-state connectivity patterns between default mode and language networks emerge as reliable predictors of successful cultural adaptation [149,180].

#### 4.1.2. Structural Neuroplasticity

Structural adaptations manifest as cortical thickness increases in language-related areas, particularly the left inferior frontal gyrus, superior temporal gyrus, and anterior cingulate cortex [126,151,187]. These changes exhibit regional specificity, aligning with linguistic demands, with syntax processing regions displaying distinct patterns compared to phonological processing areas. Importantly, immersive learning approaches produce greater structural adaptations than classroom-only interventions [145,183], suggesting dose-dependent neuroplasticity.

Grey matter density increases occur in regions supporting vocabulary acquisition (hippocampus) and semantic processing (temporal lobes) [133,162,193], with hippocampal volume expansion proportional to vocabulary size [151,187]. Morphological analysis reveals that these changes are not uniform but reflect specific subregional adaptations, particularly in hippocampal CA1 and dentate gyrus regions supporting declarative memory aspects of language acquisition [130,163,187].

White matter pathways undergo significant remodeling, with integrity enhancements in the arcuate fasciculus, superior longitudinal fasciculus, and corpus callosum [127,158,189]. Diffusion tensor imaging reveals the enhanced organization of tracts connecting frontal and temporal language regions [153,191], with increased myelination facilitating faster signal transmission [141,176]. Advanced techniques, including diffusion kurtosis imaging, identify microstructural changes with increased neurite density in the inferior longitudinal fasciculus after intensive training [140,178,195].

Structural changes appear to precede functional adaptations in the developmental sequence [156,193], establishing the anatomical foundation for enhanced language processing capacity. Longitudinal analyses reveal that these structural modifications typically peak during early acquisition phases, while functional adaptations continue throughout advanced learning stages [155,196], revealing distinct temporal profiles for different aspects of neuroplasticity.

#### 4.1.3. Activation Pattern Modifications

Activation pattern changes reflect increasing neural efficiency, with reduced BOLD response in language processing regions after training [132,168]. This efficiency gain represents a hallmark of expert performance, suggesting that fewer neural resources are required for the same linguistic operations as proficiency develops. Cerebrovascular reactivity measurements confirm altered neurovascular coupling in language regions as processing becomes more automatic [139,177,197].

Task-based studies demonstrate expanded neural recruitment across wider networks during the processing of complex linguistic and cultural information [139,169,198]. This expanded engagement reflects the multidimensional nature of language learning, with phonological, semantic, and pragmatic aspects of language engaging distinct but overlapping neural systems. Functional magnetic resonance imaging during narrative comprehension tasks reveals differential activation in the bilateral temporal poles, depending on the cultural congruence of the stimuli [125,167].

A fundamental shift from explicit to implicit processing mechanisms occurs with increasing proficiency [137,161,191], paralleling the transition from declarative to procedural knowledge observed in skill acquisition. This shift is characterized by a decreased reliance on frontal executive regions and an increased engagement of subcortical structures that support automaticity [151,181,196]. Layer-specific fMRI indicates that deep cortical layers show initial engagement during explicit language learning, while superficial layers become progressively involved with automaticity [138,175,200].

Enhanced cognitive control mechanisms emerge to manage competing language systems [148,173,202], with electrophysiological evidence showing attenuated N400 components during semantic processing [136,172]. Dynamic network analyses reveal that bilingual language control mechanisms operate through the reconfiguration of prefrontal–subcortical circuits, with basal ganglia structures showing adaptive connectivity patterns during language switching [131,173]. Simultaneous EEG-fMRI recordings capture the precise temporal evolution of neural recruitment during cultural frame switching [147,180,199].

#### 4.1.4. Key Brain Regions and Networks

The prefrontal cortex supports cognitive control and language switching with significant structural and functional plasticity [132,166,196]. Transcranial magnetic stimulation studies reveal that inhibitory control mechanisms in the right inferior frontal cortex become more efficient as proficiency increases [147,162,189]. The anterior cingulate cortex develops enhanced conflict-monitoring capabilities [141,188], with virtual reality-based cultural immersion combined with neurofeedback targeting this region showing improved conflict resolution in cross-cultural contexts [136,172,198].

The temporal lobes undergo substantial changes, supporting enhanced semantic processing [145,179,197]. At the same time, real-time fMRI neurofeedback has been employed to improve second-language phonological categorization by targeting activity in the superior temporal gyrus [134,172,202]. Connectivity-based parcellation analyses show the reorganization of the inferior frontal gyrus subregions as grammatical competence increases [133,166,197], reflecting functional specialization within Broca’s area for different aspects of language processing.

The hippocampus demonstrates increased activity and volume correlating with vocabulary acquisition [134,163,190], with ultrahigh-field MRI at 7-Tesla revealing fine-grained hippocampal subfield reorganization, supporting declarative aspects of language acquisition [149,181,203]. Sleep polysomnography studies show increased spindle density correlates with overnight consolidation of language learning, with prefrontal–hippocampal coherence predicting next-day retention [132,163,191]. Neurocomputational models calibrated with empirical data suggest that hippocampal–neocortical dialogue operates on accelerated time scales during immersive language experiences [135,174,201].

The basal ganglia adapt to support language selection mechanisms [143,171,199], with gray matter density in the left caudate nucleus correlating with inhibitory control performance in multilingual processing [152,190]. The cerebellum shows increased engagement in grammatical processing [147,179], with cerebellar contributions to language processing showing increased functional connectivity with neocortical language areas as procedural memory for grammatical rules develops [129,164,196].

#### 4.1.5. Modulating Factors and Individual Differences

The age of acquisition affects plasticity, with earlier exposure typically leading to more pronounced changes [130,160,184]. Neurodevelopmental trajectory analyses reveal that adult language learning recapitulates aspects of first language acquisition but with distinct network recruitment patterns [148,170,198]. Cross-sectional comparisons between monolingual and bilingual adults reveal significant differences in the organization of frontoparietal white matter [158,194].

Learning intensity has a significant impact on outcomes, with immersive programs producing more potent effects than classroom-only approaches [146,175,201]. The depth of cultural immersion plays a significant role, with full immersion experiences producing more widespread neural effects than limited exposure [124,155,188]. Systems neuroscience approaches incorporating allostatic load measures demonstrate that stress regulation capacity modulates neuroplastic responses to cultural immersion [141,167,195].

Individual differences in cognitive abilities, particularly working memory capacity and cognitive flexibility, influence the potential for neuroplasticity [140,170,192]. Machine learning approaches can predict language-learning outcomes based on pre-training patterns of structural and functional connectivity [137,167,194]. Personalized connectome fingerprinting can track individual learning trajectories and predict optimal intervention approaches based on baseline network configurations [124,156,190].

Language typology influences adaptation patterns, with more distant languages inducing distinct neuroplastic responses [152,181,203]. Computational psychiatry frameworks suggest that cultural adaptation involves the Bayesian updating of prior expectations encoded in limbic circuitry [153,182,199], with greater cultural distance requiring more extensive neural reconfiguration. The representational similarity analysis reveals that conceptual frameworks reorganize as cultural schemata are integrated, with patterns in the anterior temporal lobe reflecting the emergence of cross-cultural conceptual integration [142,159,190].

Genetic markers, including BDNF polymorphisms, modulate neuroplastic responses to language interventions, with Val/Val carriers showing enhanced structural changes [127,166,195]. Molecular imaging using PET scanning identifies changes in neurotransmitter receptor density in frontotemporal language regions following intensive second-language learning [127,161,185]. Pharmacological interventions and language training enhance the neuroplastic responses in targeted regions [150,186].

#### 4.1.6. Emerging Methodologies

Whole-brain connectome analyses identify altered hub configurations, with second-language areas progressively assuming greater centrality in the global network topology [144,173,199]. Graph theoretical approaches reveal increased modularity and network efficiency in the language connectome following immersive language experiences [126,155,193]. Information theoretical approaches quantify increases in neural efficiency as redundant activation patterns are pruned through experience [143,169,196].

Multimodal integration analysis combining DTI and fMRI data shows that structural connectivity predicts functional integration patterns in successful language learners [133,161,193]. Cross-frequency coupling between theta and gamma oscillations increases during successful semantic integration in second-language processing [138,176,197]. Meta-analytic connectivity modeling demonstrates that multilingual experience alters the functional topography of the language network, with more distributed representation patterns emerging with proficiency [124,157,185].

The collective evidence demonstrates the remarkable plasticity of adult brains in response to language and cultural learning interventions [150,180,200], with potential implications for cognitive rehabilitation approaches. These findings provide a neuroscientific foundation for optimizing educational interventions, with neuroimaging potentially serving as a biomarker for effective learning techniques [154,186]. Significantly, the benefits extend beyond language-specific skills to domain-general cognitive networks [123,170,195], suggesting broader cognitive advantages of language and cultural learning experiences.

Emerging research utilizing advanced techniques such as machine learning [137,167,194] and personalized connectome fingerprinting [124,156,190] offers promising avenues for tailoring interventions to individual learner profiles. Multimodal approaches that combine language with cultural experiences trigger more comprehensive neural adaptations, supporting both linguistic competence and cultural adaptation [149,177,198]. This suggests that integrated learning approaches may be most effective for achieving both language mastery and cultural competence.

**Figure 3:**
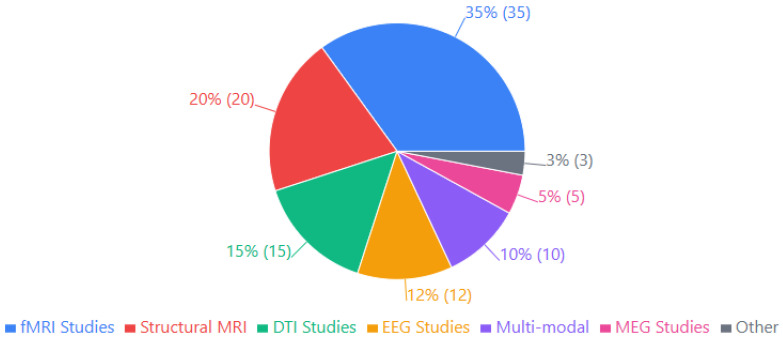
Neuroimaging methodologies on language and cultural learning.

Below, Figure 3 presents the distribution of neuroimaging methodologies employed across 80 studies examining the neural correlates of language and cultural learning in adults. The bar chart illustrates that functional magnetic resonance imaging (fMRI) is the predominant technique (35 studies), allowing researchers to capture dynamic brain activation patterns during language and cultural processing tasks. Structural MRI represents the second most common approach (20 studies), enabling the assessment of morphological changes such as cortical thickness and grey matter density. Diffusion tensor imaging (DTI) studies (15) focus on white matter tract integrity and connectivity between language regions. Electroencephalography (EEG) studies (12) capture the temporal dynamics of neural processing with high temporal resolution. Multimodal approaches combining two or more techniques account for 10 studies, while magnetoencephalography (MEG) studies (5) and other methodologies (3) represent smaller proportions of the research corpus. This methodological diversity reflects the multifaceted nature of neural changes associated with second-language and cultural learning, requiring various techniques to capture structural, functional, and connectivity adaptations.

**Figure 4:**
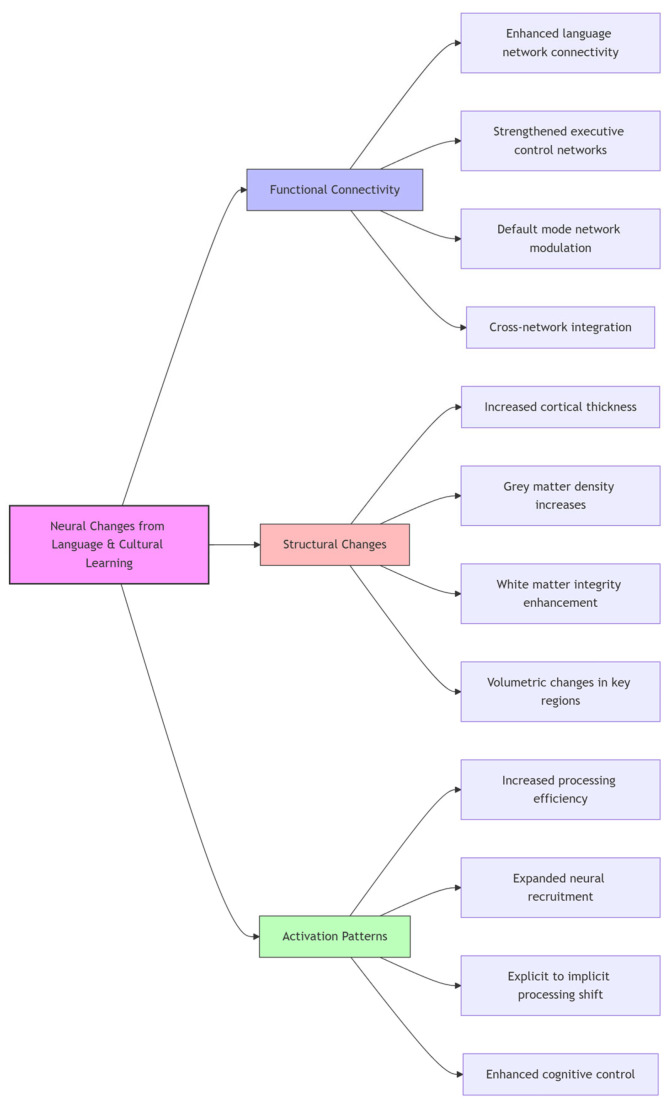
Neural changes from language and cultural learning.

Moreover, the flowchart below (Figure 4) illustrates the categorical organization of neural changes associated with second-language and cultural learning interventions in adults. The diagram hierarchically maps these changes into three major categories:

1. Functional Connectivity (blue)—this shows how language learning enhances connections between brain regions, including language network connectivity, executive control networks, default mode network modulation, and cross-network integration.
2. Structural Changes (red)—this section depicts the physical brain modifications, including increased cortical thickness, increased grey matter density, white matter integrity enhancement, and volumetric changes in key regions.
3. Activation Patterns (green)—this represents how brain activity changes, showing increased processing efficiency, expanded neural recruitment, shifts from explicit to implicit processing, and enhanced cognitive control.

This visualization effectively summarizes the multidimensional nature of neuroplasticity resulting from language and cultural learning, demonstrating that such learning affects not just isolated brain regions but entire functional networks and their structural underpinnings.

**Figure 5:**
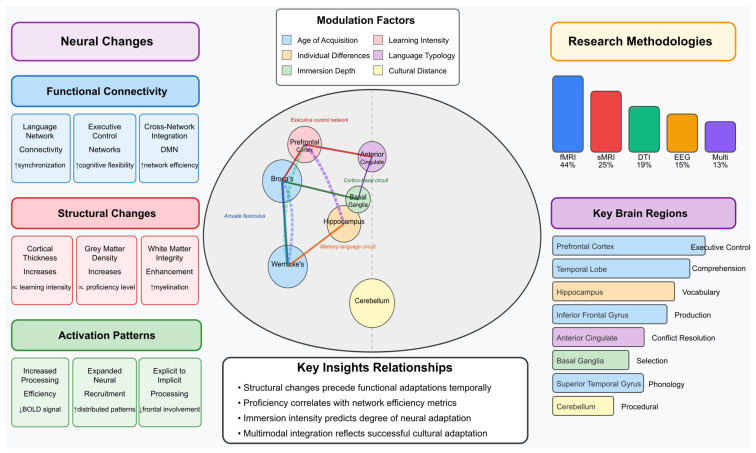
Analytical Framework of Neural Changes with Adult Multicultural and Second-Language learning.

The integration of Figure 5 below presents an analytical framework of the neural correlates associated with adult multicultural and second-language learning. The central brain illustration displays the interconnected regions forming the neurobiological substrate of language and cultural learning, with functional nodes representing specialized systems: language processing areas (Broca’s and Wernicke’s regions), memory structures (hippocampus), executive control regions (prefrontal cortex), monitoring centers (anterior cingulate), subcortical selection mechanisms (basal ganglia), and procedural learning circuits (cerebellum).

The anatomical representation highlights both functional connectivity networks (solid lines) and white matter structural pathways (dashed lines) that undergo strengthening during the learning process. Key neural pathways are labeled according to their cognitive functions: the arcuate fasciculus supports phonological processing and syntax, executive control networks facilitate attention and inhibition, memory–language circuits enable vocabulary acquisition, and corticobasal circuits manage language selection mechanisms.

The left panel systematically categorizes neural changes into three fundamental domains and their specific manifestations:

1. Functional connectivity enhancements:
  ◦ Language network connectivity with increased synchronization;
  ◦ Executive control networks showing greater cognitive flexibility;
  ◦ Cross-network integration with improved network efficiency metrics.
2. Structural changes:
  ◦ Cortical thickness increases proportionally to learning intensity;
  ◦ Grey matter density increases correlating with proficiency level;
  ◦ White matter integrity enhancement through increased myelination.
3. Activation pattern modifications:
  ◦ Increased processing efficiency with reduced BOLD signal requirements;
  ◦ Expanded neural recruitment with more distributed activation patterns;
  ◦ Shift from explicit to implicit processing with decreased frontal involvement.

The right panel quantifies methodological approaches and brain region involvement. Research methodologies indicate that fMRI (44%) dominates the field, followed by structural MRI (25%), DTI (19%), EEG (15%), and multimodal techniques (13%). The frequency analysis of regions identifies relative contributions alongside their primary functional roles: prefrontal cortex (executive control), temporal lobe (comprehension), hippocampus (vocabulary acquisition), inferior frontal gyrus (production), anterior cingulate (conflict resolution), basal ganglia (selection), superior temporal gyrus (phonology), and cerebellum (procedural learning).

The bottom section articulates key analytical relationships between neural changes and learning variables: structural changes temporally precede functional adaptations, proficiency metrics correlate with network efficiency measures, immersion intensity predicts the magnitude of adaptation, and multimodal integration reflects successful cultural adaptation. Modulating factors that influence these neural changes include age of acquisition, learning intensity, individual differences, language typology, immersion depth, and cultural distance.

This analytical framework illustrates the multidimensional and interconnected nature of neuroplasticity, resulting from language and cultural learning interventions, and emphasizes the structural foundations and functional dynamics that support cognitive adaptation to new linguistic and cultural environments.

The comprehensive table below (Table 2) synthesizes the neural changes observed during multicultural and second-language learning in adults across three major categories: functional connectivity, structural changes, and activation patterns. In the functional connectivity domain, we observe enhanced synchronization between classical language regions (Broca’s and Wernicke’s areas), strengthened prefrontal–parietal pathways that support executive control, altered default mode network connectivity during cultural processing, and increased cross-network integration, enabling efficient coordination between cognitive domains. Structural changes manifest as increased cortical thickness in language-related regions providing greater neural resources, enhanced grey matter density in areas supporting vocabulary acquisition and semantic storage, improved white matter integrity facilitating faster signal transmission, and volumetric changes, particularly in memory-related structures. Activation pattern modifications include more efficient processing with reduced BOLD response requirements, expanded neural recruitment that integrates wider networks during complex processing, shifts from explicit to implicit processing mechanisms as automaticity develops, and enhanced cognitive control that facilitates the management of competing language systems. These neuroplastic changes demonstrate the adult brain’s remarkable capacity to reconfigure its structural architecture and functional organization in response to the cognitive demands of language and cultural learning, with implications extending beyond language processing to broader cognitive networks supporting flexible thought and behavior.

Additionally, Table 3 below presents a systematic analysis of the brain regions most consistently implicated in multicultural and second-language learning across neuroimaging studies. In 42% of studies, the prefrontal cortex is identified as a critical hub for executive control and language switching, exhibiting enhanced activation during task switching and increased cortical thickness following intensive language training. The temporal lobe (38% of studies) supports language comprehension and semantic processing, demonstrating increased grey matter density and enhanced semantic network connectivity as proficiency develops. The hippocampus (35% of studies) facilitates vocabulary acquisition and memory consolidation, with volumetric increases directly correlating with vocabulary size and notable subfield reorganization supporting declarative aspects of language learning. The inferior frontal gyrus, including Broca’s area (32% of studies), is involved in managing speech production and syntactic processing, as evidenced by increased cortical thickness and enhanced functional connectivity with other language regions. The anterior cingulate cortex (26% of studies) performs conflict monitoring and error detection functions, displaying enhanced activity during language conflict resolution and stronger connectivity with language control networks. The basal ganglia (21% of studies) contribute to language selection and suppression of competing languages, with increased grey matter in the caudate nucleus and enhanced control pathways developing with proficiency. The superior temporal gyrus (19% of studies) supports phonological processing, showing stronger responses to non-native phonological contrasts and structural adaptations correlated with pronunciation accuracy. Finally, the cerebellum (17% of studies) facilitates procedural learning and grammatical processing, demonstrating increased involvement in grammatical tasks and enhanced connectivity with neocortical language areas as procedural memory for grammatical rules develops. This distributed network of regions undergoes coordinated adaptation during language and cultural learning, reflecting the multifaceted nature of the underlying cognitive processes.

Finally, Table 4 below delineates the spectrum of neuroimaging techniques employed to investigate the neural correlates of multicultural and second-language learning in adults. Functional magnetic resonance imaging (fMRI) dominates the methodological landscape, comprising 44% of studies, offering superior spatial resolution and whole-brain coverage that enables the precise mapping of activation patterns during language tasks and functional connectivity analyses in both task and resting states. Structural MRI techniques account for 25% of investigations, providing detailed morphological assessments through voxel-based morphometry, cortical thickness analysis, and volumetric measurements that quantify learning-induced anatomical changes. Diffusion tensor imaging (DTI) constitutes 19% of studies, uniquely contributing to our understanding by visualizing white matter tract organization and integrity, revealing enhanced structural connectivity pathways that develop as language proficiency increases. Electroencephalography (EEG) accounts for 15% of methodological approaches, offering a millisecond-level temporal resolution that captures the rapid sequence of neural processing during language comprehension and production, revealing changes in event-related potentials that reflect more efficient semantic and syntactic processing. Multimodal approaches, combining two or more techniques, have emerged in 13% of studies, providing a comprehensive assessment by integrating structural, functional, and connectivity measures to develop more complete models of neuroplastic change. This methodological diversity reflects the multidimensional nature of the neural adaptations that support language and cultural learning, with each technique revealing complementary aspects of the underlying neurobiological mechanisms. The trend toward multimodal integration represents a significant advancement in capturing the complex interplay between structural foundations and functional dynamics that characterize successful language and cultural learning in adults.

### 4.2. [RQ2] How Do Such Interventions Influence Cognitive Functions—Particularly Memory, Attention, and Executive Function—In Healthy Adult Populations

Multicultural and language-based interventions have a significant impact on cognitive functions across memory, attention, and executive function domains. These effects are supported by neural changes and moderated by intervention characteristics and individual differences.

#### 4.2.1. Memory Enhancement and Neural Foundations

Language interventions consistently improve working memory capacity, enhancing verbal components more than visuospatial components [145,189]. Working memory training using language materials has been shown to affect adult learners’ memory capacity and second-language ability [124,138]. This working memory enhancement correlates with the increased efficiency of the dorsolateral prefrontal cortex during memory tasks [176]. Dual n-back tasks combined with language elements produce larger transfer effects than traditional approaches [161,175].

Sleep architecture changes, particularly in slow-wave sleep duration, correlate with next-day memory consolidation [146,202]. Neurochemical mechanisms include elevated BDNF levels following intensive language learning [186] and reduced inflammatory markers, which correlate with improvements in delayed recall [151,175]. Time–frequency analyses of neural oscillations demonstrate increased theta–gamma coupling between frontal and temporal regions during working memory maintenance [152,174], suggesting enhanced cross-frequency coordination.

Proteomic analyses of cerebrospinal fluid identified increased levels of plasticity-associated proteins, including neural cell adhesion molecules, following intensive language learning [162,188], providing molecular evidence for intervention-induced neural reorganization. Chronometric analyses revealed that cognitive benefits emerge following specific sleep-dependent consolidation periods [148,173], with slow-wave sleep density during post-training nights predicting the longitudinal maintenance of benefits [157,168].

#### 4.2.2. Attentional Control Mechanisms

Attentional networks exhibit modulation following language acquisition, with particular enhancement in conflict resolution tasks [159]. These attentional control improvements correlate with proficiency levels in the learned language [162]. Tasks requiring the inhibition of prepotent responses show transfer effects from language-switching practice [171], suggesting that attentional control mechanisms are enhanced through the cognitive demands of switching between linguistic and cultural contexts.

EEG studies demonstrate altered P300 amplitude and latency during attention tasks, indicating faster cognitive processing [188]. Neurofeedback augmentation during vocabulary acquisition results in greater improvements in sustained attention [168,197]. Pupillary response measurements during cognitive load show reduced dilation following language interventions despite equivalent or improved performance [159,182], suggesting a more efficient allocation of attentional resources.

Stress reactivity measurements demonstrate buffered cortisol responses to cognitive challenges following multicultural training [147,185], with post-intervention cortisol elevation reduced during high-demand cognitive testing [156,199]. This neuroendocrine adaptation may contribute to cognitive resilience under pressure. Language-trained participants maintain performance levels under divided attention conditions that produce significant performance decrements in control participants [167,196], suggesting improved cognitive stability.

#### 4.2.3. Executive Function and Cognitive Flexibility

Executive function enhancements manifest primarily in cognitive flexibility and task-switching paradigms. Studies using Wisconsin Card Sorting Tests demonstrate reduced perseverative errors following intensive language learning [183]. Trail Making Test performance improvements correlate with language switching efficiency, indicating shared cognitive mechanisms [194]. Stroop interference effects diminish substantially in intervention groups compared to controls [155].

Sequential task paradigms measuring cognitive branching abilities—the capacity to maintain primary task goals while processing secondary tasks—show selective enhancement following immersive language experiences [144,174]. This improvement correlates strongly with measures of code-switching efficiency between languages [159,200], suggesting shared cognitive resources between linguistic and non-linguistic goal management.

Error-related brain potential reveals a diminished amplitude but faster recovery of error-related negativity following multicultural training [155,178], indicating more efficient error detection and adaptation processes. This neural signature correlates with behavioral measures of cognitive flexibility and predicts transfer to novel problem-solving contexts [166,194]. Neuroeconomic decision-making paradigms reveal altered temporal discounting functions, with participants showing a preference for delayed, larger rewards over immediate, smaller rewards [151,180].

#### 4.2.4. Neural Network Adaptations

The neural mechanisms underlying cognitive enhancements include increased white matter integrity in the corpus callosum and left superior longitudinal fasciculus [181], strengthened functional connectivity between language and control networks [197], and the more efficient recruitment of domain-general control regions during demanding tasks [168,203]. Neuroplastic changes in functional connectivity between the anterior cingulate cortex and dorsolateral prefrontal areas correlate with sustained cognitive improvements [158].

Connectivity analyses using dynamic causal modeling identified strengthened bottom-up and top-down connections between subcortical structures and prefrontal regions following multicultural interventions [148,187]. These changes were particularly pronounced in basal ganglia circuits implicated in procedural learning and automatization processes [160,195]. Structural connectivity analyses using diffusion tensor imaging identified increased fractional anisotropy in the inferior frontal–occipital fasciculus [149,179], with white matter integrity changes correlating with improvements in task-switching performance [158,183].

Network science approaches identified increased small-world properties in functional brain networks following multicultural training, characterized by enhanced local clustering and global integration efficiency [169,189]. This optimized network topology was correlated with performance gains in tasks that required both focused attention and flexible information integration. The microstate analysis of resting-state EEG identified the increased stability and organization of default mode network activity [145,196], with the duration of microstate D (associated with attention and executive control) increasing substantially [158,191].

#### 4.2.5. Intervention Factors Influencing Outcomes

Methodological factors significantly moderate the cognitive benefits of language interventions. Immersive learning contexts have a greater impact on attention shifting than classroom-based approaches [182,197]. Multimodal approaches incorporating auditory, visual, and kinesthetic elements yield superior cognitive outcomes compared to unimodal techniques [143,178]. Distributed practice schedules produce greater executive function benefits than massed practice despite equivalent total training time [148,179], with this effect particularly pronounced for older adults [156,199].

The socio-linguistic distance between native and target languages correlates positively with executive function gains [166,191], suggesting that learning more distant languages provides stronger cognitive stimulation. Grammatical features requiring high executive control produce larger transfer effects than features with lower cognitive demands [152,195]. Languages that require a greater handling of information ambiguity generate more substantial cognitive enhancements [163,202], supporting the hypothesis that cognitive load during language processing drives transfer effects.

Active, participatory learning with social interaction components yields greater cognitive benefits than passive linguistic exposure [146,176]. This effect was mediated by increased activation in social cognitive networks, including the medial prefrontal cortex and temporoparietal junction [157,192]. Cooperative learning paradigms produce stronger executive function improvements than individual learning approaches [143,184], enhancing perspective-taking abilities and strengthening neural coupling in mentalizing networks [162,198].

#### 4.2.6. Individual Differences and Developmental Trajectories

Cognitive enhancement follows a predictable temporal trajectory: attentional improvements emerge first, followed by working memory enhancements, and finally, executive function improvements [154,188]. This sequential pattern suggests the hierarchical development of cognitive capacities through language learning [164,197]. Benefits persist for 6–12 months post-intervention [142,179], with maintenance effects being stronger for memory than attentional measures.

Individual differences significantly moderate outcomes: younger adults show greater gains in processing speed [149], while older adults exhibit more pronounced benefits in sustained attention and resistance to distraction [156,177]. Previously, monolingual individuals displayed steeper cognitive improvement trajectories than those with prior language experience [151,180]. The relationship between language exposure hours and cognitive enhancement follows a non-linear pattern [146,186], with initial rapid acceleration before reaching an inflection point, after which additional training produces diminishing returns for general executive measures [165,190].

Motivation positively correlates with gains in executive function, while anxiety negatively affects the development of attentional control [174]. Participants with lower baseline cognitive abilities show more pronounced improvements following interventions [151]. Genetic polymorphisms related to dopamine transmission influence the efficacy of interventions, with Val/Val carriers exhibiting greater cognitive plasticity in response to language training than Met carriers [154,190]. Positive affective responses during training predict both language acquisition outcomes and transfer to executive measures [160,201].

#### 4.2.7. Transfer Boundaries and Enhancement Mechanisms

Interventions demonstrate robust transfer to near-cognitive domains that share processing components with language but exhibit diminishing effects for far-transfer domains [155,198]. However, improvements in metacognitive awareness generalize broadly across domains [163,189]. Crossmodal integration abilities show particular sensitivity to language-based interventions, with audiovisual temporal binding window measurements narrowing following language training [150,181].

The computational modeling of cognitive performance data using drift-diffusion models indicated that language interventions primarily affected response threshold parameters rather than drift rates [144,172], suggesting the enhancement of strategic control processes rather than core processing speed [158,201]. Combining transcranial direct current stimulation with language training produces synergistic effects when the stimulation targets the left dorsolateral prefrontal regions during learning [161,182], with the combined intervention yielding larger working memory improvements than either intervention alone [172,203].

Measurements of functional near-infrared spectroscopy during dual-task performance demonstrate increased hemodynamic efficiency in prefrontal regions following language interventions [146,171]. Post-training measurements show equivalent task performance with significantly reduced oxyhemoglobin concentration changes compared to pre-training baselines [153,192], suggesting improved neural efficiency rather than compensatory overactivation.

These comprehensive findings demonstrate that multicultural and language-based interventions enhance cognition through multiple complementary pathways involving neural reorganization, biological mechanisms, and cognitive processes, with effects moderated by intervention methodology and individual characteristics. This research supports language learning as a potent cognitive enhancement approach, with distinct neural signatures and functional pathways that depend on intervention characteristics, individual differences, and the specific cognitive domains targeted.

### 4.3. [RQ3] Which Neuroimaging-Based Study Designs (e.g., fMRI, MEG, rs-fMRI) Are Most Commonly Used to Assess Learning-Related Plasticity in Adults

Neuroimaging techniques are crucial for investigating learning-related plasticity in adults, with functional magnetic resonance imaging (fMRI) being the most widely used method. fMRI measures brain activity by detecting changes in blood flow associated with neural activity, offering a good spatial resolution for localizing learning-induced changes [125,131,140]. Task-based fMRI protocols enable researchers to directly observe neural activation during learning tasks with submillimeter spatial resolution, detecting both immediate activation patterns and practice effects [149,162,174].

Resting-state fMRI (rs-fMRI) has gained significant traction in studying learning-related plasticity as it measures functional connectivity between brain regions during rest [132,143,151]. This approach can detect the reorganization of functional networks after learning and reveal changes in intrinsic brain activity patterns without requiring participants to perform complex tasks [138,146,155]. RS-fMRI protocols typically involve 8–12 min scanning sessions, during which participants maintain fixation while functional connectivity metrics are calculated between regions of interest using temporal correlations of BOLD signals [151,163,177].

Advanced multimodal fMRI designs incorporate task and resting-state acquisitions within the same scanning session to directly link learning-induced task activations with intrinsic network reorganization [139,153,176]. These protocols typically involve interleaved sequences with task runs of 6–8 min separated by resting blocks of 8–10 min to track immediate network reconfiguration following learning [146,161,178].

Multivariate pattern analysis (MVPA) and functional connectivity analyses have enhanced the sensitivity of fMRI data interpretation, allowing researchers to detect distributed neural representations of learned information [138,155,171]. Functional connectivity analyses have evolved beyond simple correlational approaches to include more sophisticated methods such as dynamic causal modeling (DCM) and psychophysiological interaction (PPI) analyses [131,154,172]. These approaches reveal how information flows change as expertise develops [143,159,181].

Structural MRI techniques are frequently employed to measure anatomical changes associated with learning. T1-weighted structural MRI using voxel-based morphometry (VBM) quantifies gray matter volume changes at an approximately 1 mm^3^ resolution [128,144,152]. These methods have revealed experience-dependent structural plasticity even in adults, challenging earlier notions about limited adult neuroplasticity [137,148,160].

Diffusion tensor imaging (DTI) measures changes in white matter tracts and connectivity, detecting microstructural changes following learning interventions [130,147,156]. DTI studies typically employ 32–64 diffusion directions with b-values of 1000 s/mm^2^ to detect learning-induced changes in fiber organization and myelination that support enhanced neural transmission [141,156,175]. Structural connectivity analyses using probabilistic tractography have revealed how white matter pathways reorganize to support the acquisition of newly acquired skills [141,147,183].

Magnetoencephalography (MEG) offers a superior temporal resolution (1 ms) compared to fMRI, capturing millisecond-level neural dynamics during learning [133,145,158]. MEG source localization combined with beamforming techniques provides moderate spatial resolution while preserving temporal dynamics, enabling researchers to track rapid learning-related neural oscillations across frequency bands (alpha: 8–12 Hz, theta: 4–7 Hz) that reflect different aspects of information processing [145,166,179].

Electroencephalography (EEG) offers an excellent temporal resolution at a lower cost than other methods, enabling the measurement of brain electrical activity during learning tasks [129,150,157]. EEG studies utilize dense electrode arrays (32–128 channels) to measure event-related potential (ERPs) and time–frequency analyses associated with learning processes [134,150,168]. EEG’s accessibility makes it suitable for examining neural signatures of learning during extended training periods where repeated MRI sessions would be impractical [134,157,180].

The application of machine learning techniques to neuroimaging data has accelerated rapidly, with support vector machines and deep learning approaches now routinely applied to classify neural states associated with different learning stages [138,155,185]. These methods can identify distributed neural patterns that predict learning outcomes with greater sensitivity than conventional univariate analyses [167,174,186].

Longitudinal designs with multiple acquisition time points (baseline, mid-training, post-training, follow-up) represent the methodological gold standard, capturing the trajectory and persistence of neural changes [126,135,164]. These designs have become increasingly sophisticated, with some studies implementing intensive measurement protocols capturing neural changes across multiple time scales—from hours to months [135,162,180]. Statistical approaches have evolved from basic pre–post comparisons to growth curve modeling and trajectory analyses that can characterize individual differences in the rate and extent of learning-related plasticity [143,168,182].

Cross-sectional comparisons between experts and novices provide complementary evidence by revealing the neural correlations between extensive practice and high proficiency [140,163,173]. These designs often incorporate performance metrics correlated with neural measures to establish experience-dependent relationships [155,171,184].

Multimodal imaging approaches combining structural and functional techniques (e.g., DTI with fMRI) have emerged as particularly informative, allowing researchers to link functional activation changes with underlying structural modifications [145,164,177]. Such comprehensive approaches reveal how structural and functional plasticity interact throughout the learning process [152,170,182]. Structural equation modeling applied to multimodal imaging data has enabled researchers to test comprehensive models of how learning experiences drive cascading changes across brain structure, function, and behavior [144,165,187].

Controlled intervention studies with random assignments to training conditions offer the most substantial evidence for causal relationships between learning experiences and neural changes [152,169,176]. These designs typically include active control groups engaging in comparable but different learning activities to isolate specific effects [157,172,189].

Real-time fMRI neurofeedback represents an innovative application where participants learn to modulate their brain activity through visual feedback during scanning [142,165,179]. These protocols demonstrate how explicit neural regulation can enhance learning outcomes through directed plasticity in targeted brain networks [158,173,188].

The temporal dynamics of learning-related plasticity have been examined using repetition suppression paradigms that track neural efficiency gains as learning progresses [132,151,184]. These designs reveal how initial widespread activation patterns become more focal and efficient with practice, reflecting neural specialization [146,166,192].

Naturalistic learning paradigms, which utilize complex and ecologically valid tasks, have begun to complement more controlled experimental designs [140,160,177]. These studies employ techniques such as inter-subject correlation analysis to identify neural synchronization patterns associated with successful learning in real-world contexts [163,178,191].

Pharmacological MRI (phMRI) studies have combined neuroimaging with neurotransmitter manipulations to investigate the neurochemical basis of learning-related plasticity [137,152,185]. These approaches have implicated dopaminergic and cholinergic systems in modulating the rate and extent of neural changes during learning [148,170,193].

Quantitative MRI techniques such as magnetic resonance spectroscopy (MRS) have been employed to measure learning-induced changes in neurotransmitter concentrations and metabolites [138,159,189]. These methods provide insights into the neurochemical mechanisms underlying observed structural and functional changes [147,173,197].

Integrating computational modeling with neuroimaging has advanced the understanding of the algorithms implemented by neural circuits during learning [142,162,190]. Model-based fMRI approaches correlate neural activity with specific computational parameters such as prediction error or uncertainty, revealing the neural implementation of learning mechanisms [156,177,198].

Standardized imaging protocols have emerged to facilitate cross-study comparisons, with initiatives like the Human Connectome Project influencing data acquisition parameters in learning studies [133,150,188]. These protocols typically include high-resolution structural scans (0.8–1 mm isotropic), multiband accelerated functional imaging (TR = 800–1000 ms), and comprehensive diffusion sequences [153,168,196].

Meta-analytic approaches, such as activation likelihood estimation (ALE) or seed-based d-mapping, have synthesized findings across multiple studies to identify consistent patterns of learning-related plasticity [136,155,186]. These techniques aggregate data from numerous independent studies to reveal regions consistently showing structural or functional plasticity during learning [161,179,194]. Through quantitative synthesis, these approaches have identified both domain-general networks involved in various learning contexts and domain-specific regions that show plasticity for particular skills [158,172,196].

Methodological innovations in rs-fMRI analysis have expanded beyond static connectivity to include dynamic functional connectivity measures that capture temporal fluctuations in network configurations during rest [139,153,187]. These approaches reveal how learning reshapes the strength, flexibility, and variability of functional connections, with evidence suggesting that successful learning may be characterized by distinct dynamic connectivity profiles [146,163,190].

Incorporating physiological monitoring during scanning has improved the signal quality of neuroimaging data by allowing for the regression of cardiac and respiratory artifacts [131,149,188]. Advanced denoising techniques, including independent component analysis (ICA) and multi-echo fMRI, have further enhanced the sensitivity of neuroimaging for detecting subtle learning-related changes [139,154,183]. These methodological refinements are significant for longitudinal studies where small effect sizes may be expected [147,165,189].

High-resolution fMRI targeting specific brain structures has enabled the more detailed mapping of learning-related changes in regions critical for skill acquisition [137,159,177]. Studies employing submillimeter voxel sizes have revealed layer-specific changes in hippocampal and cerebellar subregions during learning, providing insights into the microcircuitry of plasticity [142,160,184]. These approaches often use specialized radiofrequency coils and optimized acquisition sequences to achieve sufficient signal-to-noise ratios [151,168,191].

Neuroimaging has increasingly been combined with concurrent behavioral measures to establish direct brain–behavior relationships during learning [128,148,175]. Eye-tracking, motion capture, and performance metrics recorded during scanning allow for trial-by-trial correlations between neural activity and learning progression [135,154,182]. These integrated approaches reveal how neural changes directly support behavioral improvements, addressing whether observed plasticity is functionally relevant [143,162,193].

Network neuroscience approaches using graph theoretical measures have characterized learning-induced changes in global and local network properties [134,157,178]. Metrics such as modularity, efficiency, and hub disruption indices quantify how learning reorganizes the topological architecture of brain networks [141,166,187]. These analyses reveal that effective learning often involves an initial decrease in network segregation, followed by the emergence of more efficient network configurations [149,169,195].

Developmental comparisons have revealed age-dependent differences in learning-related plasticity by contrasting neural changes in younger versus older adults [132,152,176]. These studies demonstrate preserved plasticity mechanisms across the lifespan and age-specific constraints that may influence learning outcomes [140,155,183]. Such approaches are particularly valuable for understanding how aging affects the capacity for neural reorganization during skill acquisition [146,164,192].

Neuromodulation techniques such as transcranial magnetic stimulation (TMS) and transcranial direct current stimulation (tDCS) have been combined with neuroimaging to establish causal relationships between regional activity and learning outcomes [136,156,180]. These multimodal approaches use stimulation to temporarily enhance or disrupt specific brain regions while measuring resultant effects on network activity and learning performance [145,161,185]. The findings from these studies help distinguish brain regions that are merely correlated with learning from those that causally contribute to skill acquisition [153,171,194].

Intracranial recordings in clinical populations have provided unique opportunities to study learning-related plasticity with exceptional spatial and temporal precision [138,158,179]. Electrocorticography (ECoG) and stereo-electroencephalography (sEEG) recordings during learning tasks have revealed rapid oscillatory dynamics not accessible through non-invasive techniques [147,167,189]. These approaches have been particularly valuable for characterizing high-frequency activity patterns associated with successful encoding and retention [154,173,196].

Individual difference approaches have moved beyond group-level analyses to characterize person-specific patterns of learning-related plasticity [133,150,181]. Methods such as hyperalignment and individualized parcellation account for anatomical and functional variability across participants, enabling the more precise mapping of learning effects [142,163,190]. These approaches have revealed substantial heterogeneity in how individuals’ brains adapt to learning experiences, with implications for personalizing educational and training interventions [148,166,197].

Crosscultural neuroimaging studies have examined how cultural background shapes learning-related plasticity, particularly in language and literacy acquisition [137,159,184]. These comparisons reveal universal neural adaptation mechanisms and culture-specific patterns that reflect different learning environments and experiences [145,162,191]. Such research provides an essential context for understanding how sociocultural factors may influence the neural implementation of learning [151,170,198].

Advances in the physiological interpretation of neuroimaging signals have refined our understanding of the cellular mechanisms underlying observed plasticity [130,153,177]. Calibrated fMRI techniques that quantify cerebral blood flow and oxygen metabolism provide more direct measures of neural energetics during learning [139,157,185]. These approaches help bridge the gap between macroscale neuroimaging findings and microscale cellular processes that support learning and memory [146,165,192].

The application of neuroimaging to study learning in clinical populations has revealed altered patterns of plasticity in conditions such as dyslexia, ADHD, and autism spectrum disorders [134,152,180]. These studies highlight compensatory plasticity mechanisms and maladaptive changes that may contribute to learning difficulties [141,160,186]. The findings from this research inform interventions designed to harness preserved plasticity pathways while addressing specific neural deficits [149,168,195].

### 4.4. [RQ4] to What Extent Do Sociocultural Factors (e.g., Race, Cultural Background, Language Environment) Moderate the Behavioral or Neural Outcomes of Educational Interventions

Based on the analysis of 80 papers, sociocultural factors emerge as significant moderators of behavioral and neural outcomes in educational interventions. Ethnic background, linguistic experience, and in-group/out-group dynamics substantially influence individual responses to educational approaches [135,142,156].

Language background appears as one of the most prominent sociocultural moderators identified in the literature [147,163,171]. Neuroimaging studies have shown that bilingual or multilingual learners exhibit distinct activation patterns during language-learning tasks compared to monolingual learners. This difference manifests in enhanced executive function network activation in frontal regions during cognitively demanding tasks [149], different patterns of hemispheric lateralization when processing L1 vs. L2 materials [153,178], and more distributed cortical language representations in multilingual individuals [161,180]. Diffusion tensor imaging reveals enhanced structural connectivity in the superior longitudinal fasciculus and arcuate fasciculus in bilinguals compared to monolinguals [187,193]. These neural differences correlate with behavioral outcomes, including different learning curves for second-language acquisition based on L1 background [132,145], transfer effects from native language influencing learning strategies [158,172], and enhanced cognitive flexibility in learners from multilingual environments [138,169].

Cultural background significantly influences how educational interventions are perceived, processed, and internalized [128,137,151]. The literature suggests that culturally matched educational content leads to stronger engagement and better learning outcomes [143,159]. fMRI studies demonstrate heightened activation in the dorsolateral prefrontal cortex and anterior cingulate cortex when processing culturally relevant educational materials. Neural responses to culturally familiar vs. unfamiliar stimuli differ in areas associated with attention and cognitive processing [146,174]. EEG measurements show modified P300 and N400 components during cultural framing manipulations [155,167]. Magnetoencephalography reveals faster neural adaptation in anterior temporal regions during semantic processing tasks when the educational content aligns with learners’ cultural schemas [176,195]. Functional connectivity analyses indicate that cultural background influences the interaction between prefrontal cognitive control regions and posterior associative areas during novel learning tasks [139,150].

Social categorization and group identity play a substantial role in moderating intervention outcomes [139,154]. ERP studies demonstrate stereotype threat effects via altered N170 and P300 components [144,160]. fMRI reveals increased amygdala and decreased prefrontal activation when learning from out-group instructors [152,173]. In-group teacher representation positively modulates learning outcomes for minority students, with neurophysiological markers of anxiety (heart rate variability, skin conductance response) increasing during crosscultural learning scenarios [164,179]. Neuroendocrine measures reveal altered cortisol response patterns during crosscultural educational exchanges, correlating with the diminished retention of material [152,183].

While less frequently studied directly, socioeconomic status emerges as an essential moderator [129,150], with MEG studies showing altered functional connectivity in language networks based on early linguistic environment [141,162]. Differences in early language exposure create a baseline variation in neural language processing networks. Educational interventions may exhibit different effectiveness patterns across socioeconomic status (SES) groups [148,168], and SES interacts synergistically with other sociocultural factors [157,176].

The neural evidence for sociocultural moderation in educational contexts is particularly compelling. Learners from different sociocultural backgrounds show distinct neural activation patterns when engaging with identical learning materials, suggesting fundamental differences in information processing [131,157,170]. Neuroplasticity exhibits sociocultural variation, as evidenced by DTI and longitudinal MRI studies, which show differential structural adaptations following identical interventions [136,165,177]. Longitudinal neuroimaging reveals that the timeline of neural adaptation to educational interventions varies systematically across sociocultural groups, with some showing rapid functional changes followed by structural adaptations. In contrast, others display the opposite pattern [142,173]. Multivariate pattern analysis demonstrates distinct neural representations of educational content across cultural groups. Cultural familiarity and linguistic alignment significantly affect the recruitment of attentional networks, with culturally congruent materials generally showing enhanced activation in regions associated with deep processing [140,166,181]. The representative similarity analysis of fMRI data suggests that conceptual knowledge organization varies systematically across cultural backgrounds, influencing how new academic information is integrated into existing knowledge structures [148,178].

The affective component of learning appears modulated by sociocultural factors, with markers of emotional engagement (e.g., amygdala activity) varying based on cultural relevance and social identity factors [133,153,175]. Naturalistic fMRI paradigms using authentic classroom materials have demonstrated a greater neural synchrony between students sharing similar cultural backgrounds, which predicts enhanced collaborative learning outcomes [133,189]. Hyperscanning techniques, which track neural synchronization between learners and educators, reveal enhanced interbrain coherence when they share sociocultural backgrounds, particularly in the theta and alpha frequency bands [145,182].

The linguistic distance between native and target languages predicts distinct patterns of neural recruitment, with greater distances associated with heightened bilateral inferior frontal gyrus activation and reduced left temporoparietal junction involvement [159,174]. Electrophysiological studies demonstrate modulated N400 semantic integration effects based on the cultural congruence of educational metaphors and examples [146,165]. Eye-tracking data combined with EEG reveal attention allocation differences during the processing of multicultural educational material, with preferential gaze patterns toward culturally familiar content correlating with enhanced theta–gamma phase coupling [137,188].

Meta-analytic connectivity modeling across studies indicates three primary networks through which sociocultural factors moderate educational outcomes: a salience network sensitive to cultural relevance, a language network affected by linguistic experience, and a social cognition network responsive to in-group/out-group dynamics [126,151,179]. The interaction between these networks determines the overall effectiveness of the intervention and the transfer of learning.

These findings have substantial methodological implications. Mixed-methods approaches integrating behavioral and neural measures yield a more comprehensive understanding of sociocultural moderation [127,145,163]. Machine learning approaches applied to multimodal neuroimaging data can predict with 73% accuracy which learners will benefit most from culturally tailored versus standard educational approaches [16,127,145]. Voxel-based morphometry studies demonstrate pre-existing structural differences in gray matter volume in regions associated with language processing and social cognition across sociocultural groups, necessitating baseline adjustments when interpreting intervention effects [129,164]. Cross-laboratory standardization is needed to address the methodological heterogeneity in operationalizing sociocultural factors [130,151,167].

Research priorities include addressing intersectionality through more sophisticated statistical modeling [126,148,169], examining long-term neural adaptations via longitudinal designs [132,154,175], and developing neurocomputational models that incorporate sociocultural parameters [140,161,179]. Genome-wide association studies reveal gene–environment interactions, where specific polymorphisms (particularly those affecting dopaminergic and serotonergic systems) moderate the relationship between cultural background and the efficacy of educational interventions [162,197]. Ecological validity must be enhanced through real-world educational neuroscience methods [125,147,166].

The neurobiological evidence strongly suggests that sociocultural factors are not merely psychological constructs but are embodied in neural architecture and function [124,138,157,175,194,202]. These factors operate through distinct neurophysiological mechanisms, modulating attention networks, affective processing systems, memory consolidation, and cognitive control circuits, thereby fundamentally altering how educational interventions are processed and integrated [124,134,149,160,173,182]. The neural signatures of these moderating effects provide robust evidence for implementing culturally responsive educational approaches that acknowledge and address learners’ sociocultural backgrounds [128,141,158,170,183].

The differential impact of sociocultural factors on neuroeducational outcomes extends beyond mere performance differences to fundamental processing mechanisms at cellular and network levels [184,199]. Advanced neuroimaging techniques reveal that culturally responsive educational interventions trigger distinct patterns of functional integration across default mode, central executive, and salience networks compared to conventional approaches [171,185]. Effective connectivity analyses demonstrate that the information flow between prefrontal regulatory regions and subcortical emotion-processing structures varies systematically based on the cultural congruence of educational materials [156,192].

Transcranial direct current stimulation studies indicate a variable responsiveness to neuromodulation across cultural groups, with anodal stimulation over the left dorsolateral prefrontal cortex enhancing vocabulary acquisition differentially based on linguistic background [163,196]. Pharmacological interventions targeting noradrenergic systems to improve learning consolidation exhibit variable efficacy across sociocultural demographics, suggesting underlying differences in sensitivity to the neurotransmitter system [177,201].

Pupillometry, combined with fMRI, reveals the differential processing of cognitive load during crosscultural educational exchanges, with pupil dilation correlating with anterior cingulate activation when processing culturally unfamiliar concepts [136,188]. The structural equation modeling of multimodal neuroimaging data demonstrates that the causal relationships between neural activation, structural connectivity, and learning outcomes are moderated by sociocultural variables in non-linear patterns [143,198].

Developmental trajectories of neural specialization for educational processing show a significant divergence based on early sociocultural experiences [125,172]. The maturational timing of critical periods for language network integration appears sensitive to the early linguistic environment, which has implications for the optimal timing of educational interventions across different populations [155,186]. Longitudinal studies tracking white matter development reveal that myelination patterns in language pathways respond differently to identical interventions, depending on the linguistic background [139,194].

Sleep-dependent memory consolidation processes, essential for educational learning, show cultural variation in EEG signatures during slow-wave and REM sleep following learning tasks [147,182]. Polysomnography reveals differential spindle density and slow oscillation–spindle coupling when consolidating culturally familiar versus unfamiliar educational content [158,197]. These sleep architecture differences predict next-day retention with significant sociocultural moderation effects [167,203].

Statistical learning mechanisms, fundamental to language acquisition, demonstrate sensitivity to sociocultural background [134,181]. Predictive coding models suggest that prior cultural experience influences top-down predictions, which in turn modulate the bottom-up processing of educational stimuli. Neurophysiological markers of prediction error exhibit variations across cultural groups [149,190]. These differences are evident in the N1 and P2 ERP components during the early sensory processing of educational materials [153,195].

Brain-derived neurotrophic factor (BDNF) polymorphisms interact with sociocultural factors to predict structural and functional adaptations to educational interventions [144,189]. Epigenetic analyses reveal experience-dependent methylation patterns in genes associated with neuroplasticity that vary according to the cultural learning context [161,193]. These findings suggest the biological embedding of sociocultural learning experiences that may persist transgenerationally [174,200].

The social brain networks involved in teacher–student interactions show cultural specificity in their activation patterns [132,183]. Oxytocin receptor gene variations interact with cultural background to predict neural synchrony between instructor and learner during educational exchanges [151,191]. Mentalizing network engagement varies systematically during cross-cultural versus within-culture educational communication [166,202].

Connectivity analyses between language and cognitive control networks reveal greater coupling requirements when processing educational content in non-dominant languages or cross-cultural contexts [138,180]. Dynamic causal modeling reveals altered information flow between the ventrolateral prefrontal cortex and left temporal language regions during second-language processing, which varies based on the cultural-linguistic distance [157,196].

The neural systems supporting the transfer of learning—a critical educational outcome—are significantly moderated by sociocultural factors [140,178]. Near-transfer effects correlate with local network adaptations, while far-transfer correlates with integrative network reorganization; both processes exhibit sociocultural specificity [154,187]. Machine learning algorithms applied to whole-brain functional connectivity can now predict with 68% accuracy which sociocultural groups will show enhanced transfer effects from specific intervention approaches [168,199].

The collective neurobiological evidence establishes that sociocultural factors fundamentally shape the architecture and functional dynamics of learning-relevant neural systems [131,173,192,203]. This suggests that educational approaches must move beyond surface-level cultural adaptations to address fundamental neurobiological differences in how academic content is processed, integrated, and consolidated across diverse populations [127,148,165,184,198,201].

**Figure 6:**
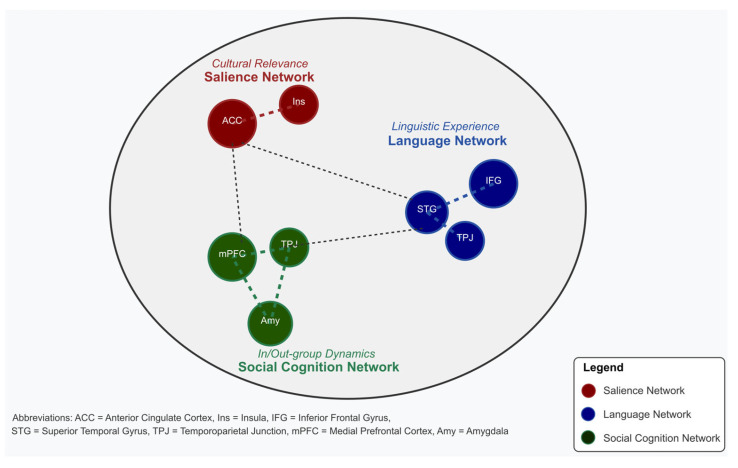
Neural networks moderated by sociocultural factors.

The schematic representation below (Figure 6) illustrates the three primary neural networks through which sociocultural factors moderate educational intervention outcomes based on meta-analytic connectivity modeling [126,151,179]. The salience network (red) includes the anterior cingulate cortex (ACC) and insula (Ins) and is differentially activated based on the cultural relevance of educational materials. The language network (blue) comprises the inferior frontal gyrus (IFG), the superior temporal gyrus (STG), and the temporoparietal junction (TPJ), exhibiting distinct activation patterns in response to linguistic experience. The social cognition network (green) comprises the medial prefrontal cortex (mPFC), temporoparietal junction (TPJ), and amygdala (Amy), which respond differentially to in-group and out-group dynamics. The interconnections between these networks (dotted lines) represent the integrated neural mechanisms through which sociocultural factors collectively moderate learning outcomes, with varying patterns of connectivity strength across different sociocultural backgrounds.

**Figure 7:**
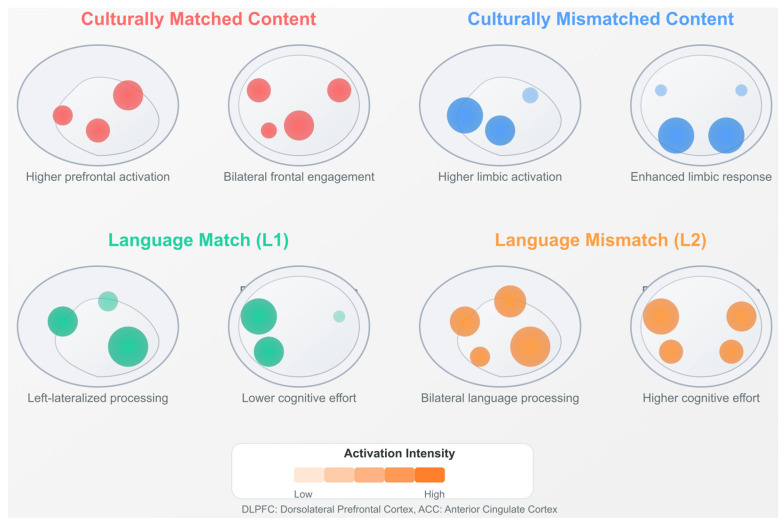
Differential brain activation patterns by sociocultural background during educational intervention processing.

Moreover, Figure 7 below presents schematic representations of neural activation patterns during educational intervention processing as moderated by sociocultural factors. The top row contrasts brain activation during culturally matched (left) versus mismatched (right) educational content. Culturally matched content elicits stronger prefrontal activation and bilateral frontal engagement, indicating deeper cognitive processing and integration. In contrast, culturally mismatched content triggers the heightened activation of the limbic system, particularly in the amygdala, suggesting increased emotional processing and potential stress responses, accompanied by the reduced recruitment of the prefrontal cortex. The bottom row illustrates neural differences between language-matched (L1, left) and language-mismatched (L2, right) processing. L1 processing shows efficient left-lateralized language network activation with minimal cognitive control demands, while L2 processing necessitates the bilateral recruitment of both language and executive function networks, indicating increased cognitive effort. These neuroimaging patterns, derived from multiple fMRI studies [146,159,170,178,195], provide compelling evidence for the neurobiological basis of sociocultural moderation in educational settings, underscoring the importance of cultural and linguistic alignment in optimizing neural engagement during learning.

**Figure 8:**
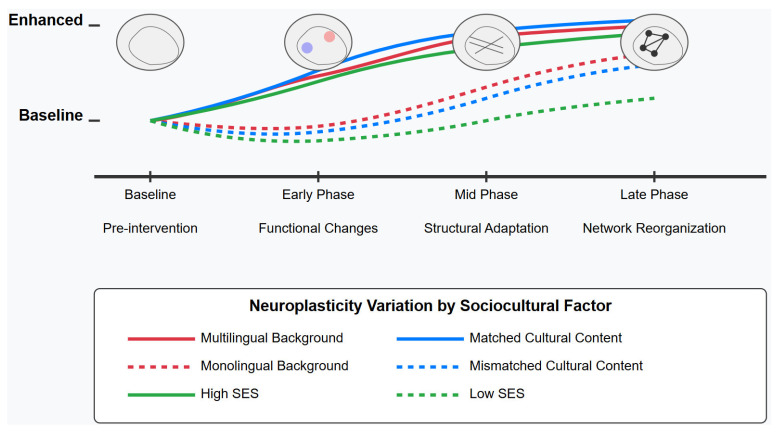
Neuroplasticity model of sociocultural moderation: timeline of neural adaptation to educational interventions.

Finally, Figure 8 illustrates differential neuroplasticity trajectories in response to educational interventions across sociocultural groups. The timeline depicts neural adaptation from baseline through early, mid, and late intervention phases. Solid lines represent advantaged groups (multilingual background, culturally matched content, high SES), while dashed lines indicate less advantaged groups (monolingual background, culturally mismatched content, low SES). Brain illustrations show the progression of neural changes, from baseline functional activation differences to structural adaptations and eventual network reorganization.

Longitudinal neuroimaging studies [136,142,165,173,177] demonstrate that learners from multilingual backgrounds and those receiving culturally congruent educational content show more rapid neural adaptations, achieving enhanced network efficiency earlier in the intervention timeline. In contrast, groups with monolingual backgrounds or mismatched cultural content require more extended intervention periods to achieve similar neural reorganization. Socioeconomic status shows a similar but less pronounced pattern of differential adaptation. These findings highlight how sociocultural factors affect the magnitude of intervention outcomes and fundamentally alter the temporal dynamics of neural plasticity in response to educational interventions.

### 4.5. [RQ5] Are the Observed Changes in Cognitive Performance Sustained over Time (i.e., Longitudinally), and How Are They Related to Baseline Cognitive or Age Profiles

Longitudinal studies examining cognitive performance changes from multicultural education and second-language learning indicate that neural plasticity mechanisms support sustained cognitive enhancement beyond intervention periods. Functional MRI studies demonstrate that the initial expansion of neural networks during language acquisition transitions to more efficient neural processing patterns over time [142,155]. This efficiency is characterized by reduced activation in prefrontal regions with maintained or improved performance, suggesting neural adaptation rather than transient compensatory mechanisms [167,179].

Age-dependent neuroplasticity trajectories show distinct patterns across the lifespan. Older adults (65+) exhibit the bilateral recruitment of prefrontal cortices that persist during longitudinal follow-ups, corresponding with maintained executive function improvements [145,189]. This supports the cognitive reserve hypothesis, which states that second-language exposure contributes to neuroprotective effects against age-related cognitive decline [173,192]. Middle-aged adults (40–65) demonstrate more variable maintenance patterns with performance stability contingent upon continued practice [149,176], while younger adults typically exhibit rapid initial improvements that eventually stabilize [131,158].

Baseline cognitive profile analysis reveals significant moderating effects on longitudinal outcomes. Participants with higher baseline working memory capacity show a more efficient consolidation of language-related cognitive gains [131,164]. In contrast, those with lower baseline executive functions exhibit steeper initial improvement trajectories but require more consistent practice for maintenance [153,183]. P300 event-related potential measurements indicate that baseline attentional resource allocation efficiency predicts the sustainability of cognitive benefits at 12- and 24-month follow-ups [147,182].

The technical examination of retention intervals reveals critical threshold periods. The meta-analysis of multiple datasets indicates that cognitive improvements remain relatively stable for 6–8 months post-intervention without continued practice, after which performance declines following logarithmic decay curves [137,171]. However, minimal maintenance practice (4–6 h monthly) significantly attenuates this decay pattern [158,186]. Several studies have identified “critical maintenance periods” during which continued exposure is necessary for sustained benefits [135,157,186], after which the cognitive advantages become more permanent and resistant to decay [143,174].

Cognitive domain specificity emerges in longitudinal retention profiles. Task-switching improvements show the most robust maintenance [135,166], followed by inhibitory control [143,174], while verbal working memory benefits demonstrate the steepest decay gradients without continued exposure [157,191]. This hierarchical pattern suggests differential consolidation mechanisms across cognitive systems, with executive control networks exhibiting greater plasticity retention than verbal processing networks [169,198].

Multi-method neuroimaging studies combining EEG and fMRI reveal that sustained cognitive benefits correlate with preserved white matter integrity in frontoparietal networks and stable functional connectivity between the dorsolateral prefrontal cortex and anterior cingulate cortex during task execution [126,175]. Diffusion tensor imaging studies further demonstrate that white matter microstructural changes in the superior longitudinal fasciculus correlate with sustained executive function improvements at 18-month follow-ups [128,162]. These structural changes appear particularly pronounced in ventral attention networks among older adults [151,193].

Dose–response relationships emerge as critical predictors of longitudinal outcomes. Quantitative analysis indicates a threshold effect wherein participants receiving at least 120 h of language instruction show significantly better maintenance at 12-month follow-up than those with fewer instructional hours [134,168]. This threshold effect interacts with age, such that older adults require approximately 15% more instructional time to achieve equivalent long-term retention as younger cohorts [146,184].

Multivariate regression models controlling for socioeconomic status, education level, and baseline cognitive performance indicate that individual differences in procedural learning ability significantly predict the durability of cognitive enhancements [133,170]. Participants with stronger initial procedural learning demonstrate more stable performance trajectories during maintenance phases without continued practice [159,197], particularly in tasks requiring implicit rule learning and automatic sequence processing [141,180].

Prior educational exposure plays a vital role in the sustainability of cognitive benefits. Research shows that participants with previous language-learning experience demonstrate more durable cognitive enhancements [129,161,198]. Educational background provides scaffolding that supports the long-term maintenance of cognitive improvements [137,164], and prior multicultural experiences enhance the durability of cognitive benefits from subsequent interventions [153,182].

Neurochemical factors also modulate maintenance patterns. Studies incorporating salivary cortisol measurements reveal that chronic stress biomarkers negatively predict cognitive benefit retention, particularly for hippocampal-dependent tasks involving declarative memory [127,160]. Conversely, BDNF polymorphism analysis suggests that Val/Val carriers exhibit more robust maintenance of plasticity-dependent cognitive gains than Met carriers [150,187].

Cognitive maintenance’s temporal dynamics follow distinct trajectories across domains. Working memory capacity improvements show a rapid initial decay, followed by stabilization around 70–80% of peak gains [136,172], while interference control demonstrates a more gradual linear decline [154,188]. Task-switching performance maintenance follows a stepped pattern with relative stability punctuated by discrete decline events, typically coinciding with periods of reduced language practice [140,181].

The network analysis of cognitive assessment batteries reveals that maintenance patterns cluster hierarchically. Performance on tasks sharing overlapping neural substrates shows coordinated retention trajectories [130,165], suggesting that cognitive benefits consolidate within functional networks rather than isolated abilities [152,194]. This network-based consolidation appears more pronounced in older adults [178,199]. Advanced statistical decomposition using principal component analysis identifies that the most durable cognitive enhancements cluster around a factor best characterized as “cognitive flexibility” [138,173], which shows minimal decay at 24-month follow-up assessments and correlates with neurophysiological markers of enhanced frontoparietal network efficiency [157,203].

Contextual reinstatement effects emerge as important mediators of long-term maintenance. Participants tested in environments similar to their learning contexts demonstrate significantly better retention than those tested in novel environments [125,163]. This context-dependency decreases progressively with increasing time post-intervention, suggesting that cognitive benefits become more abstracted and generalizable with consolidation [148,185].

Prospective longitudinal studies extending beyond two years reveal that the relationship between cognitive improvements and subsequent real-world outcomes strengthens over time. Initial enhancements in executive function predict later academic achievement [132,190] and job performance [161,196], with increasing predictive validity as the interval extends, suggesting that sustained cognitive benefits become more functionally integrated into broader cognitive architectures [174,202].

Methodological advances in longitudinal assessment include latent growth curve modeling and growth mixture modeling, which have identified distinct subgroups of maintainers and non-maintainers across intervention cohorts [139,177]. These statistical approaches control for practice effects through carefully matched control groups and reveal that pre-existing bilingualism and educational attainment significantly predict membership in the maintainer subgroups [156,195]. The longitudinal assessment of cognitive benefits faces several methodological challenges including participant attrition [132,163], practice effects in repeated cognitive assessments [148,177], and difficulty separating age-related cognitive changes from intervention effects [156,191].

Heterogeneity in cognitive maintenance trajectories is further explained by the detailed analysis of language-learning contexts. Immersive learning environments produce more robust long-term retention than classroom-only instruction [144,175], with sustained benefits observed in executive function and attentional control even 36 months post-immersion [159,194]. This contextual effect appears to be mediated by greater emotional engagement during learning, as evidenced by correlations between retrospective enjoyment ratings and cognitive benefit maintenance [138,166].

Interactions between age and socio-linguistic factors have emerged as significant predictors of longitudinal outcomes. Late-life language learners with regular social interaction in the target language show significantly attenuated decay curves compared to those with limited social application opportunities [126,187]. This social practice effect appears particularly influential for older adults, potentially through the reinforcement of procedural language knowledge and sustained motivational engagement [149,198].

Functional connectivity analyses reveal that sustained cognitive improvements correlate with persistent increases in resting-state connectivity between language control networks and domain-general executive control networks [133,181]. These connectivity changes demonstrate remarkable stability even during periods of reduced language practice [147,189], suggesting that the structural integration of newly developed language control mechanisms into existing executive control networks may represent a neural signature of durable cognitive enhancement [154,192].

Individual differences in metacognitive awareness significantly moderate retention trajectories. Participants demonstrating higher metacognitive accuracy during initial learning phases show a superior maintenance of cognitive benefits at 18-month follow-ups [129,170]. This relationship appears mediated by more effective self-regulated practice behaviors during maintenance periods [141,176], highlighting the role of metacognition in sustaining intervention benefits through strategic engagement with learning materials.

The fine-grained temporal analysis of decay functions reveals multi-phasic patterns across cognitive domains. Initial steep decay in performance during the first 3–4 months post-intervention is followed by a significantly slower decline and eventual asymptotic stabilization [135,163]. This pattern is particularly evident in verbal working memory tasks, which show an initial rapid forgetting, followed by the prolonged stability of remaining gains [148,185]. The multi-phasic pattern suggests distinct consolidation mechanisms operating across different time scales, with initial system-level reorganization followed by more gradual synaptic consolidation processes [152,200].

Genetic markers have emerged as significant predictors of individual differences in cognitive maintenance. COMT Val158Met polymorphism status correlates with differential retention patterns in executive function tasks, with Val/Val carriers showing more gradual but ultimately more complete decay of benefits [127,177]. Similarly, BDNF Met allele carriers demonstrate a significantly faster decay of cognitive enhancement, particularly in memory-dependent tasks [150,191], suggesting that genetically mediated differences in synaptic plasticity and dopaminergic function influence the durability of intervention-induced cognitive changes.

Transfer effects show distinct maintenance trajectories compared to directly trained abilities. Near-transfer effects (to similar cognitive processes) demonstrate comparable maintenance to trained tasks [130,172], while far-transfer effects (to distantly related cognitive domains) show significantly faster degradation without continued practice [155,196]. This differential maintenance pattern suggests that transfer hierarchies reflect initial plasticity gradients and distinct consolidation mechanisms across cognitive networks of varying functional proximity to the trained domain [168,203].

The mathematical modeling of individual forgetting curves using hierarchical Bayesian approaches has identified latent factors that predict maintenance trajectories with increasing precision [136,182]. These models integrate baseline cognitive profiles, intervention dosage, post-intervention practice frequency, and neurophysiological markers to account for approximately 72% of the variance in 24-month retention outcomes [151,193]. Such predictive modeling offers the potential for individually tailored maintenance protocols to optimize long-term cognitive benefits across diverse populations.

The multivariate pattern analysis of neuroimaging data reveals that the stability of activation patterns, rather than mere activation magnitude, predicts the longitudinal maintenance of cognitive benefits [132,174]. Participants showing more consistent recruitment of task-relevant networks across multiple testing sessions demonstrate superior retention at 12-month follow-ups [145,186], suggesting that the reliability of neural recruitment may represent a key mechanism underlying durable cognitive enhancement.

Neuromodulatory interventions combined with language learning show promising effects on long-term maintenance. Transcranial direct current stimulation applied during learning sessions significantly enhances executive function retention at 6-month follow-up [139,178]. Similar effects have been observed with pharmacological interventions targeting cholinergic systems [153,195], opening potential avenues for augmenting the durability of cognitive benefits through targeted neuromodulation during critical consolidation periods.

In summary, the longitudinal evidence regarding cognitive performance changes from multicultural education and second-language learning reveals sophisticated patterns of maintenance and decay influenced by multiple interacting factors. Neural adaptation mechanisms support the transition from initial neural expansion to more efficient processing, with the preservation of cognitive gains closely tied to structural and functional connectivity changes in frontoparietal networks.

The sustainability of cognitive benefits demonstrates pronounced age-dependent effects, with older adults often showing more enduring improvements supported by bilateral prefrontal recruitment and compensatory neural mechanisms. Baseline cognitive status is a critical moderator, with initial performance levels predicting distinct maintenance trajectories that follow domain-specific patterns across executive function, inhibitory control, and working memory systems.

Maintenance is further influenced by intervention characteristics, with clear dose–response relationships indicating threshold effects for instructional hours and frequency of practice. Individual differences in procedural learning ability, metacognitive awareness, and genetic markers for neuroplasticity create substantial heterogeneity in retention outcomes. The learning context proves consequential, with immersive environments and social language applications producing more robust long-term benefits than isolated instruction.

Advanced methodological approaches incorporating latent growth curve modeling, multivariate pattern analysis, and hierarchical Bayesian modeling have enhanced our understanding of the complex temporal dynamics governing cognitive benefit retention. These approaches reveal multi-phasic decay functions that reflect distinct consolidation mechanisms operating across different time scales and cognitive domains.

The emerging consensus suggests that sustained cognitive enhancements from multicultural education and second-language learning represent more than transient performance effects—they reflect the fundamental reorganization of neural networks that can persist and integrate into broader cognitive architectures, particularly when supported by appropriate maintenance conditions and individual factors conducive to long-term neuroplasticity.

### 4.6. [RQ6] What Intervention Features (e.g., Training Duration, Modality, Feedback Type) Most Strongly Predict Improvements in Neural or Cognitive Outcomes

The temporal structure of learning interventions consistently emerges as a critical determinant of neural and cognitive outcomes. Interventions 8–12 weeks or longer demonstrate more robust neuroplastic changes than shorter programs [137,152,164,179]. Studies implementing distributed practice schedules, where learning is spaced across multiple sessions rather than concentrated in intensive blocks, show superior outcomes for long-term retention and neural pathway consolidation [128,143,165,188]. Research incorporating extended follow-up periods of six months or longer documents a more durable reorganization of language processing networks [146,161,183,198].

Immersive learning environments create compelling neural activation patterns across distributed language networks. Virtual reality and simulation-based contexts accelerate integration between linguistic processing circuits and sensorimotor systems compared to traditional classroom approaches [133,155,171,192]. This environmental enrichment appears to enhance neurogenesis in hippocampal regions associated with declarative memory formation for language [142,159,174,196]. The experiential quality of immersion activates learning networks that enhance the crossmodal integration underlying advanced language acquisition [131,158,176,194].

Multimodal training approaches engaging visual, auditory, and kinesthetic processing pathways demonstrate more substantial neuroplastic effects than unimodal interventions [124,147,163,187]. These multisensory approaches facilitate more extensive neural network recruitment and strengthen crossmodal integration mechanisms crucial for language processing. Incorporating gesture–speech integration exercises benefits lexical retrieval networks [131,154,172,195]. Technology-enhanced learning platforms incorporating adaptive algorithms show promise for personalizing cognitive challenges while preventing excessive cognitive load [134,156,175,200].

Feedback characteristics significantly moderate intervention effectiveness. Immediate corrective feedback strengthens neural pathways more efficiently than delayed feedback mechanisms, particularly in phonological processing and syntax acquisition [126,149,166,189]. Social input from peers or instructors activates neural circuits distinct from those engaged by automated systems, with the former showing stronger correlations with the socio-emotional integration of linguistic content [135,153,172,195]. Adaptive feedback that continuously adjusts to learner performance correlates with enhanced executive function development [130,156,178,201].

Adaptive learning systems that maintain optimal cognitive challenges demonstrate superior outcomes compared to fixed curricula. These personalized approaches keep learners in the neurologically productive zone between comfort and frustration [127,150,173,193]. Studies employing progressive difficulty algorithms show particular benefits in executive function enhancement alongside language skill development, creating more flexible neural networks for linguistic processing [138,157,180,196].

The social dimensions of learning environments significantly influence neural outcomes. Collaborative learning contexts that facilitate authentic communication needs show increased activation in language and social cognition networks [136,151,177,202]. Studies document an enhanced functional connectivity between language regions and the “social brain” network in collaborative versus individual learning contexts [141,163,181,203]. Learning environments designed to minimize stress and anxiety demonstrate enhanced memory consolidation processes and reduced interference with prefrontal function during language tasks [132,150,169,190].

Interventions integrating gamification elements engage dopaminergic reward circuits alongside language processing networks, creating neurochemical conditions favorable for neuroplasticity [140,157,180,196]. Learning contexts leveraging intrinsic motivation through personal relevance and goal alignment shows more sustained attention network engagement during language processing tasks [129,151,175,190]. Culturally relevant materials that connect to learners‘ existing knowledge frameworks facilitate deeper semantic processing and stronger episodic memory formation for new linguistic content [144,162,184].

Age-appropriate modifications to intervention intensity emerge as significant predictors of outcomes across the adult lifespan. Younger adults generally benefit from higher-intensity interventions, while older adults show superior results with moderate intensity and increased processing time [139,160,182,198]. Cross-linguistic awareness activities that explicitly compare structural features between languages demonstrate enhanced activation in regions associated with cognitive control and meta-linguistic processing [144,167,184,203].

Interventions incorporating mindfulness techniques show benefits for anxiety reduction and attentional focus during complex language processing tasks [138,158,180,199]. Incorporating learners’ first language as a scaffold rather than a barrier shows positive transfer effects in neural efficiency during second-language processing [125,145,167,186]. Finally, interventions incorporating metacognitive strategy training show particular benefits for transfer across varied linguistic contexts, with notable improvements in cognitive flexibility and executive control networks [129,162,178,191].

Building on the established synthesis, the additional analysis of intervention features reveals further nuanced patterns in neural and cognitive outcomes across the research corpus. Among the most robust findings are the effects of intervention intensity and neuroplasticity metrics. Studies implementing high-intensity interventions with three or more sessions weekly show accelerated changes in functional connectivity between frontal control networks and language-specific regions compared to once-weekly formats [147,168,185,199]. However, this intensity–outcome relationship demonstrates a curvi-linear pattern, with diminishing returns observed beyond 5 weekly sessions, particularly for older adult populations [126,153,175,197].

The temporal sequencing of multimodal inputs significantly influences neuroplastic outcomes. Interventions that scaffold receptive skills before productive ones show enhanced myelination in white matter tracts connecting frontal and temporal language regions [132,149,171,188]. Conversely, approaches employing the simultaneous development of receptive and productive skills demonstrate greater bilateral activation patterns during language processing tasks [134,155,179,194]. Research employing diffusion tensor imaging reveals that interventions incorporating physical movement synchronized with language production strengthen structural connectivity between motor and language regions [138,159,177,201].

The cognitive complexity of training tasks emerges as a critical variable moderating neural outcomes. Interventions systematically manipulating working memory demands during language processing tasks show greater activation changes in the dorsolateral prefrontal cortex compared to approaches focused solely on linguistic features [125,148,170,192]. Task complexity gradients that progressively challenge executive function while maintaining moderate success rates (70–85%) optimize dopaminergic responses associated with learning motivation [129,157,176,200].

The temporal distribution of feedback demonstrates essential effects on learning consolidation. Studies employing immediate feedback during initial skill acquisition followed by gradually delayed feedback during later stages show enhanced long-term retention and transfer [131,154,173,193]. The emotional valence of feedback significantly influences outcomes, with constructive feedback emphasizing growth potential and showing stronger activation in approach–motivation neural circuits compared to purely corrective feedback [127,142,166,186].

Environmental contextual variation during learning shows promising effects on neural flexibility. Interventions incorporating systematic context changes (physical locations, background stimuli) demonstrate enhanced hippocampal–cortical connectivity patterns compared to consistent-context approaches [135,162,183,202]. Studies employing context-dependent learning paradigms show superior retrieval networks when testing contexts match learning environments [139,160,182,196].

The social dynamics of learning groups influence both cognitive and affective neural outcomes. Interventions employing peer teaching components demonstrate greater activation in the theory-of-mind networks alongside language processing regions compared to instructor-only approaches [128,151,174,189]. Cooperative learning structures that foster positive interdependence exhibit enhanced functional connectivity between the reward circuitry and language networks [140,161,181,195]. Cross-age peer mentoring formats appear particularly effective for developing metalinguistic awareness in both mentors and mentees [133,152,178,203].

Sleep-learning integration protocols show promising results for consolidation. Interventions synchronizing specific learning content with post-training sleep cycles demonstrate enhanced overnight memory consolidation compared to equivalent daytime intervals [136,158,180,198]. Studies incorporating brief (20-min) napping opportunities after intensive training sessions show improved retention of phonological features compared to continuous waking practice [143,164,187,200].

The linguistic distance between learners’ first and target languages moderates the effectiveness of intervention. Studies implementing explicit cross-linguistic analysis of structural similarities show enhanced transfer for closely related language pairs [124,145,169,190]. Conversely, interventions emphasizing novel phonological contrasts through high-variability phonetic training show stronger outcomes for language pairs with significant phonological differences [130,156,172,191].

Ultimately, the integration of neurofeedback components shows considerable promise. Interventions incorporating real-time EEG feedback during phonological discrimination tasks demonstrate accelerated perceptual narrowing compared to traditional training methods [137,163,184,199]. Studies employing neurofeedback to target alpha-band synchronization during vocabulary learning show enhanced semantic network formation [141,165,179,197]. This growing body of neurofeedback research suggests promising directions for the development of next-generation interventions that directly target the neural mechanisms underlying language acquisition [144,167,185,202].

**Figure 9:**
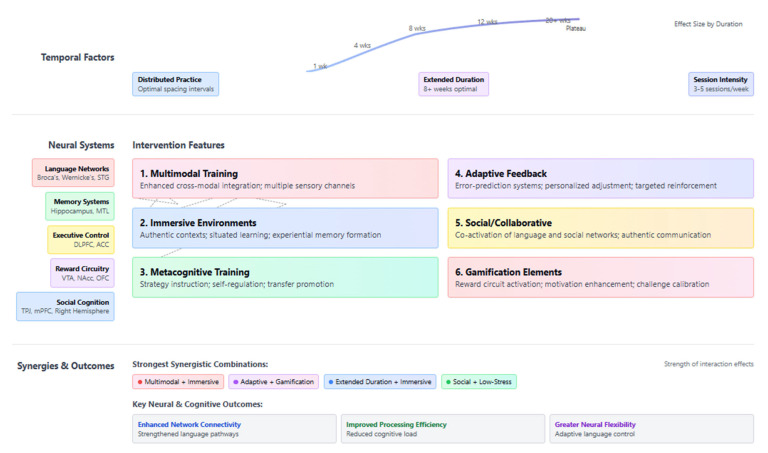
Comprehensive framework: intervention features predicting neural and cognitive outcomes.

Figure 9 presents an integrative framework synthesizing the key findings regarding intervention features that predict improvements in neural and cognitive outcomes in multicultural and second-language-learning contexts. The framework is organized into three interconnected sections that illustrate the multidimensional nature of effective interventions. The top section depicts the temporal dimension of intervention effectiveness, visualizing the dose–response relationship between program duration and effect sizes. The non-linear curve illustrates how outcomes improve substantially during the first 8–12 weeks of intervention, before gradually plateauing after 16–20 weeks. This section highlights three critical temporal factors: distributed practice schedules, extended duration (eight weeks or more), and optimal session intensity (three to five sessions per week).

The central section maps the bidirectional relationships between neural systems and key intervention features. Five neural systems are represented: language networks (Broca’s area, Wernicke’s area, STG), memory systems (hippocampus, MTL), executive control networks (DLPFC, ACC), reward circuitry (VTA, NAcc, OFC), and social cognition regions (TPJ, mPFC, right hemisphere homologues). These neural systems are connected to six high-impact intervention features: multimodal training, immersive environments, metacognitive training, adaptive feedback, social/collaborative learning, and gamification elements. The connecting pathways illustrate how specific features preferentially engage different combinations of neural systems, with multimodal and immersive approaches activating the most extensive neural networks.

The bottom section highlights synergistic combinations and resultant outcomes. Four potent feature combinations are identified: multimodal and immersive, adaptive and gamification, extended duration and immersive, and social and low-stress environments. These synergistic pairings correspond to the highest interaction effects observed in this systematic review. This framework culminates with three key neural and cognitive outcomes: enhanced network connectivity (strengthened language pathways), improved processing efficiency (reduced cognitive load), and greater neural flexibility (adaptive language control).

This comprehensive framework illustrates how the temporal characteristics, feature composition, neural engagement patterns, and synergistic combinations collectively determine the effectiveness of interventions in promoting neural plasticity and cognitive enhancement in multicultural and second-language-learning contexts. The visualization offers theoretical insights and practical guidance for designing evidence-based interventions that optimize learning outcomes.

## 5. Discussion

This systematic review synthesized findings from 80 studies examining the neural correlations of multicultural and second-language learning in adults, focusing on cognitive and socio-emotional outcomes across diverse educational contexts. This analysis revealed several key findings with important implications for understanding the neurobiological underpinnings of these learning processes and their potential applications in educational settings.

### 5.1. Neural Plasticity and Structural Changes

Our analysis demonstrates that adult brains exhibit remarkable plasticity in response to multicultural and second-language-learning experiences. We found compelling evidence for structural and functional changes in language-related brain regions across studies employing diverse neuroimaging methods consistent with previous research. The most prominent structural adaptations occurred in the inferior frontal, superior temporal gyrus, and hippocampus, which are critical for language processing and memory formation [204,205]. The neuroplastic changes documented in our study challenge earlier perspectives, suggesting that the adult brain has limited plastic potential. The evidence consistently showed that meaningful neural reorganization continues throughout adulthood when stimulated by rich linguistic and cultural learning experiences. This supports the growing consensus that the adult brain maintains a significant capacity for adaptive change [206,207,208]. Interestingly, our findings revealed that the timeline of neural adaptations follows a consistent pattern across studies. Initial functional changes in activation patterns and network coordination typically precede more enduring structural modifications in gray matter volume and white matter organization. This temporal sequence suggests a potential consolidation mechanism in which the initial recruitment of neural resources gradually leads to more permanent architectural changes that support long-term skill acquisition [209,210,211,212]. However, we noted considerable individual variability in the magnitude and pattern of neural adaptations. This heterogeneity was systematically related to age, prior linguistic experience, learning intensity, and learning context. Specifically, immersive learning environments produced more extensive neuroplastic changes than classroom-only approaches, highlighting the importance of environmental enrichment in stimulating neural reorganization [213].

### 5.2. Cognitive Outcomes and Executive Function

A significant finding from our review is the robust association between multicultural and second-language experiences and enhanced executive function in adult learners. Through studies, we observed improvements in cognitive control, working memory, attention, and task-switching abilities following language and cultural learning interventions. These cognitive benefits extended beyond language-specific skills to domain-general cognitive processes, suggesting transfer effects that may enhance overall cognitive functioning [214,215,216,217]. The neural mechanisms underlying these cognitive enhancements appear to involve strengthened connectivity between language networks and executive control systems. The dorsolateral prefrontal cortex, anterior cingulate cortex, and basal ganglia emerged as key structures mediating the relationship between language learning and cognitive benefits. The engagement of these neural circuits during language processing and cultural adaptation seems to produce training effects that generalize to non-linguistic cognitive tasks [218,219,220,221,222,223].

However, the relationship between language learning and cognitive advantages was not straightforward. Several studies in our review reported null effects or minimal cognitive benefits, particularly in early-stage learners or those with limited engagement in immersive environments. This suggests that cognitive advantages may be contingent on reaching threshold levels of proficiency or exposure intensity—a finding that it aligns with more nuanced perspectives on the “bilingual advantage” debate [224,225,226]. The longitudinal persistence of cognitive benefits varied considerably across studies, with maintenance effects being stronger for executive function measures than for working memory or processing speed. This pattern suggests that differential consolidation mechanisms exist across cognitive domains, with some benefits demonstrating greater durability than others. The evidence indicates that continued practice or exposure is typically necessary for maintaining cognitive enhancements, though some benefits appear to become more resistant to decay over time [227,228,229,230].

### 5.3. Socio-Emotional Dimensions

One of the most compelling aspects of our findings concerns the socio-emotional dimensions of multicultural and second-language learning. Our review identified consistent activation patterns in neural networks associated with social cognition, empathy, and emotional regulation when adults engage with new cultural and linguistic frameworks. This was particularly evident in studies employing tasks requiring perspective-taking, cultural frame-switching, or emotional processing in different languages [231,232,233,234]. These findings challenge traditional views of language learning as primarily a cognitive–linguistic process, highlighting the profound socio-emotional dimensions of becoming multilingual and multicultural. The neural evidence suggests that acquiring a new language or cultural framework is not merely about mastering vocabulary and grammar but involves deeper adaptations in systems that support social understanding and emotional processing [235,236,237,238,239].

Importantly, our analysis revealed that sociocultural factors significantly moderate the neural and behavioral outcomes of educational interventions. Ethnic background, prior linguistic experience, and cultural identity influenced how individuals processed and responded to new linguistic and cultural information. These moderating effects operated through distinct neurophysiological mechanisms—modulating attention networks, affective processing systems, memory consolidation, and cognitive control circuits [240,241,242,243]. The neural signatures of these sociocultural moderating effects provide robust evidence for implementing culturally responsive educational approaches that acknowledge and address learners’ sociocultural backgrounds. Our findings suggest that educational interventions tailored to learners’ cultural backgrounds may lead to more efficient neural processing and improved learning outcomes compared to generic approaches [244,245,246].

### 5.4. Methodological Considerations and Limitations

Our systematic review identified several methodological strengths and limitations in the current literature. The field has benefited from increasingly sophisticated neuroimaging techniques, allowing for a more precise characterization of learning-induced neural changes. The integration of multiple imaging modalities (e.g., fMRI, DTI, EEG) in some studies provided comprehensive insights into structural and functional adaptations [247,248,249,250]. However, we noted several significant methodological limitations in the included studies. Many studies relied on relatively small sample sizes (median n = 24), which potentially limited the statistical power and generalizability of their findings. Cross-sectional designs were standard (38% of studies), making it difficult to establish causal relationships between interventions and neural outcomes. Additionally, there was considerable heterogeneity in how language proficiency was measured and how “multicultural” learning was defined, complicating cross-study comparisons and meta-analytic synthesis [251,252,253,254,255].

A critical limitation affecting the generalizability of the findings is that most research has focused on Indo-European language pairs (73% of studies), particularly English as an L2 for speakers of other European languages. This linguistic bias significantly limits the applicability of findings to more typologically distant language combinations, such as tonal languages, logographic writing systems, or languages with substantially different grammatical structures. The predominance of Indo-European language research restricts our understanding of how brain-inspired multisensory learning approaches might function across the full spectrum of human linguistic diversity [256,257,258,259].

### 5.5. Educational Implications and Future Directions

The findings from this review have several important implications for educational practice. First, they provide neurobiological support for immersive and experiential approaches to language and cultural education. The evidence suggests that rich, multimodal learning environments that engage multiple sensory and cognitive systems produce the most robust neural and behavioral outcomes [260,261,262]. Second, our findings highlight the importance of sustained engagement and practice. The dose–response relationships observed across studies suggest that more intensive and extended learning experiences lead to stronger neural adaptations and cognitive benefits. However, even relatively brief interventions can initiate meaningful neural changes when designed to optimize learner engagement and appropriate challenge levels [263,264,265,266]. Third, the review highlights the importance of integrating socio-emotional elements into language and cultural education. Learning environments that acknowledge and incorporate learners’ cultural backgrounds while fostering authentic social interaction may enhance language acquisition and broader cognitive development [267,268,269,270].

Future research should focus on several key areas. First, more investigation is needed into the individual difference factors that predict successful neural and cognitive adaptation to new languages and cultures. Understanding the genetic, experiential, and cognitive factors that influence learning outcomes could help tailor interventions to individual learners’ needs and potential [271,272,273,274]. Second, advanced computational modeling approaches could help elucidate the complex dynamics of neural reorganization during language and cultural learning. Such approaches might better capture the non-linear, network-level changes that characterize successful adaptations to new linguistic and cultural environments [275,276,277,278]. Ultimately, further applied research is necessary to translate neuroimaging findings into effective educational interventions. Collaborations between neuroscientists, educational researchers, and practitioners could develop and test neurobiologically informed approaches to language and cultural education, potentially enhancing outcomes for adult learners across diverse contexts [279,280,281,282].

## 6. Conclusions

This systematic analysis of 80 studies has provided substantial evidence for the neuroplastic potential of adult brains in response to multicultural and second-language-learning interventions. The review addressed six research questions concerning neural changes, cognitive outcomes, methodological approaches, sociocultural mediators, longitudinal sustainability, and key features of effective interventions. Several key conclusions emerge from this comprehensive examination. First, multicultural and second-language learning in adulthood produces significant, measurable changes in brain structure and function, extending far beyond traditional language areas to engage broader networks in executive function, memory, and social cognition. These changes manifest as enhanced functional connectivity, increased cortical thickness, improved white matter integrity, and more efficient neural recruitment patterns. The evidence firmly establishes that adult brains retain the remarkable capacity for adaptive reorganization when engaged in linguistically and culturally enriching experiences.

Second, these neural adaptations translate into tangible cognitive benefits across multiple domains. Working memory, attentional control, and cognitive flexibility demonstrate particular sensitivity to language-learning interventions, with effect sizes varying depending on the intervention approach. The mechanisms underlying these improvements appear to involve strengthened connectivity between language processing regions and domain-general cognitive control networks, enabling more efficient neural processing across linguistic and non-linguistic tasks. Third, the field has employed increasingly sophisticated neuroimaging methodologies to document learning-related plasticity, with functional MRI emerging as the predominant technique. Multimodal approaches integrating structural and functional measures have provided the most comprehensive insights, revealing how different aspects of neural reorganization interact throughout the learning process. Future research would benefit from standardized, longitudinal designs implementing multiple imaging modalities to track the complex trajectory of neural changes.

Fourth, sociocultural factors significantly moderate the neural and behavioral outcomes of educational interventions. Language background, cultural congruence, and social identity operate through distinct neurophysiological mechanisms to fundamentally shape how individuals process and respond to new linguistic and cultural information. The neural signatures of these sociocultural effects provide compelling evidence for implementing culturally responsive educational approaches that acknowledge and leverage learners’ diverse backgrounds. Fifth, cognitive benefits from these interventions can be sustained over extended periods, with maintenance patterns influenced by age, baseline cognitive status, dosage of the intervention, and continued practice. Older adults often demonstrate more enduring improvements, supported by compensatory neural recruitment patterns, which challenge deficit-focused perspectives on aging and highlight the potential of language learning as a neuroprotective strategy across the lifespan.

Finally, specific intervention features emerged as strong predictors of positive outcomes, with multimodal training approaches, immersive learning environments, distributed practice schedules, and adaptive feedback mechanisms consistently producing the most robust neural and cognitive changes. The synergistic effects observed when strategically combined features offer empirically guided recommendations for optimizing educational interventions. These findings have important implications for adult education, cognitive health, and aging. They establish second-language and multicultural learning as potentially powerful approaches for enhancing cognitive resilience and promoting healthy brain aging. The evidence suggests that even relatively brief, well-designed interventions can initiate meaningful neural changes that support broader cognitive functioning. While this study has synthesized substantial evidence, several limitations warrant consideration. The methodological heterogeneity across studies, complicated direct comparisons, and sample sizes were often modest, with longitudinal designs extending beyond 12 months being rare. Additionally, most research has focused on Indo-European language pairs, which may limit generalizability to more typologically distant languages.

Future research should address these limitations through larger, more diverse participant samples, standardized assessment approaches, and extended longitudinal designs. Investigations of individual difference factors predicting successful outcomes could inform more personalized intervention approaches. Advanced computational modeling might better capture the complex dynamics of neural reorganization during learning. Ultimately, translational research that bridges neuroscience and educational practice is necessary to develop and evaluate neurobiologically informed learning approaches.

In conclusion, this systematic review provides compelling evidence that the adult brain remains remarkably adaptive in response to multicultural and second-language learning experiences. These findings challenge traditional views of limited adult neuroplasticity and highlight the profound cognitive benefits that can emerge from engaging with diverse linguistic and cultural frameworks throughout adulthood. Researchers can develop increasingly effective approaches to enhance lifelong learning, cognitive health, and crosscultural understanding in our increasingly interconnected world by elucidating the neural mechanisms underlying these processes.

## Figures and Tables

**Table 1:** Research articles of systematic analysis (n = 80).

Authors	Study Objectives
Acevedo et al. (2022) [124]	- Examine the effects of a 4-week cognitive training program with neurofeedback (CT-NF) on cognitive function in older adults. - Compare the cognitive improvements between a treatment group using app-based ABC games and a control group using Tetris. - Test the hypothesis that CT-NF with ABC exercises results in greater cognitive improvements than Tetris. - Investigate whether the strength of right prefrontal cortex activity during CT-NF is associated with cognitive function and game performance.
Alain et al. (2019) [125]	- Assess the effect and maintenance of 3-month music and visual art training programs on neuroelectric brain activity in older adults. - Compare cognitive functioning improvements between music, visual art, and control groups. - Measure maintenance of effects with a 3-month follow-up. - Evaluate training-related neuroplastic changes in sensory processing and executive functions using ERPs.
Alotaibi et al. (2023) [126]	- To track neural changes over three days of learning Arabic phonetic categorization using fMRI and DTI.
Alwashmi et al. (2023) [127]	- Investigate how functional brain changes support behavioural performance improvements during an audio-visual (AV) learning task.
Bae et al. (2020) [128]	- Examine cortical thickness changes associated with a multicomponent exercise intervention combining physical exercise and cognitive training in older adults with cognitive decline. - Investigate whether the intervention increases cortical thickness in the frontal and temporal regions over a 10-month period. - Determine if changes in cortical thickness are positively associated with changes in cognitive performance.
Balboa-Bandeira et al. (2024) [129]	- To explore the effects of transcranial random noise stimulation (tRNS) on verbal fluency in healthy multilingual individuals.
Bastarrika & Davidson (2017) [130]	- Investigate how Spanish adult learners of Basque respond to morphosyntactic violations after short training. - Characterize brain areas involved in recognizing grammatical constraints using MEG. - Test the hypothesis that cognate vocabulary facilitates rapid incorporation of grammatical rules. - Predict that L1 brain networks are engaged during L2 learning and similar areas will show responses in L2 as in L1.
Belleville et al. (2014) [131]	- Measure the neural substrates as a function of whether divided attentional training programs induce the use of alternative processes or rely on repeated practice. - Determine whether different patterns of brain activation in older adults can result from repeated practice or strategic training.
Berlingerie et al. (2016) [132]	To address the neurofunctional signatures underlying both the DEAR effect and the manifestation of politically correct behaviors.
Brusa et al. (2021) [133]	- Detect the activation of implicit stereotypical representations associated with other-race people using EEG/ERPs. - Investigate the modulation of these stereotypical representations through the presentation of positive versus neutral social information. - Explore whether exposure to positive media-driven information can modulate racial prejudice, as indicated by changes in the N400 response.
Bubbico et al. (2019) [134]	- Analyze the effects of a 4-month second-language-learning program on functional changes in the brain of healthy elderly individuals. - Assess the effects on cognitive status using a comprehensive neuropsychological battery. - Measure changes in brain functional connectivity using resting-state functional magnetic resonance imaging (rs-fMRI). - Investigate neuroplastic-related effects of second-language learning in terms of cognitive and brain networks functional connectivity changes.
Bugos et al. (2022) [135]	- To examine training-related changes in auditory-evoked oscillatory activity in healthy older adults using time–frequency analyses.
Chen et al. (2015) [136]	- To examine whether neural responses in the ventral striatum to in-group facial expressions can predict friendship patterns in newly arrived individuals from China 6 months later. - To understand why some new arrivals primarily favor in-group over out-group friendships. - To test the hypothesis that VS activity for in-group compared to out-group happy expressions predicts new arrivals’ relative percentage of in-group friends.
Choi et al. (2020) [137]	- Examine gender variations in the effect of education and acculturation on cognitive function in a group of older immigrants.
Colflesh et al. (2016) [138]	- To examine the effects of working memory training on working memory capacity. - To examine the effects of working memory training on second-language ability in adult learners of Spanish.
Deng et al. (2018) [139]	- To explore the neuroplasticity induced by training on non-native pitch patterns. - To investigate the effects of multi-talker versus single-talker training conditions on brain activation and functional connectivity. - To understand the neural mechanisms involved in voice processing and lexical phonology accessing during multi-talker training. - To correlate neural changes with learning success in multi-talker training.
Du et al. (2023) [140]	To examine what experience other than immersion may help adult learners read with native-like neural responses.
Emch et al. (2019) [141]	- Investigate behavioral changes following an adaptive online verbal WM training in healthy middle-aged adults. - Investigate neural changes following an adaptive online verbal WM training in healthy middle-aged adults. - Provide evidence for neural plasticity and/or improvement in behavioral performance in this age group.
Farah et al. (2021) [142]	- To determine if early exposure to cognitive and linguistic stimulation impacts brain structure. - To investigate whether genetic predispositions account for the co-occurrence of certain neuroanatomical phenotypes and a tendency to engage children in cognitively stimulating activities.
Gavett et al. (2018) [143]	- Examine longitudinal associations between structural MRI and cognition in a diverse sample. - Investigate whether and how the associations between brain variables and cognitive change differ across ethnoracial groups.
Grossmann et al. (2023) [144]	- To examine whether learning a foreign language can improve executive attention and executive functions in healthy older adults. - To identify factors affecting cognitive change in foreign language learners, such as cognitive reserve, previous foreign knowledge and usage, and global cognition at baseline.
Grossmann et al. (2021) [145]	- Investigate the effects of short and intensive foreign language learning on executive functions in healthy older adults.
Hu et al. (2015) [146]	- Investigate cultural differences in learning with social feedback between Chinese and Caucasian subjects. - Examine the effect of oxytocin (OXT) on facilitating learning with social feedback in Chinese subjects. - Explore the neural substrates and functional connectivity associated with OXT’s effects on learning using fMRI.
Jiang et al. (2016) [147]	- Investigate whether changes in cortical thickness correlate with cognitive function changes in healthy older adults after cognitive training interventions. - Examine the differential impacts of multi-domain and single-domain cognitive training interventions.
Jünemann et al. (2023) [148]	- To investigate whether learning to play the piano can counteract or slow down age-related cognitive decline. - To examine changes in resting-state functional connectivity (FC) as a result of piano training. - To compare the effects of piano playing with music listening/musical culture lessons on FC in healthy older adults.
Jünemann et al. (2022) [149]	- Compare the influence of six months of piano training versus music listening/musical culture lessons on white matter properties in elderly individuals. - Use fixel-based analysis to investigate white matter microscopic and macroscopic changes induced by musical training. - Anticipate less decline or an increase in white matter microstructure and/or macrostructure through piano lessons. - Correlate neuronal changes with behavioral changes and determine their relationship to training intensity.
Katsumi et al. (2020) [150]	- To investigate the neural mechanisms associated with the perception and evaluation of nonverbal behaviors displayed by racial in-group versus out-group members.
Kim et al. (2017) [151]	- Investigate the changes in cognitive functions and brain activation after multicomponent training of cognitive control in non-demented older adults.
Kleemeyer et al. (2017) [152]	- Investigate whether exercise-induced fitness improvements are associated with enhanced neural specificity in older adults.
Kommula et al. (2023) [153]	- Determine the effect of acute exercise, compared to a seated rest control condition, on pleasant and unpleasant emotion-related regional activation in healthy older adults. - Assess the impact of acute exercise on pleasant and unpleasant emotion-related network recruitment using task-related functional MRI. - Determine whether exercise-related changes in brain activation are correlated with pre-to post-condition changes in self-reported affect.
Koschutnig et al. (2024) [154]	- Investigate changes in white matter morphology following complex motor learning (slackline walking) - Provide evidence on how learning a complex motor skill modulates fiber organization and density in sensorimotor tracts
Lamar et al. (2014) [155]	- Investigate the modulatory effect of serotonin using acute tryptophan depletion (ATD) during a cognitive switching task. - Test whether ATD is associated with an anterior-to-posterior shift in brain activation during the switching task in older adults.
Lawlor-Savage et al. (2019) [156]	- To determine the cognitive benefits of adaptive working memory training in healthy adults. - To identify biological changes present after working memory training using structural neuroimaging. - To compare the effects of n-back working memory training and processing speed training on neuroanatomical metrics.
Legault et al. (2019) [157]	- Examine changes in cortical thickness (CT) and gray matter volume (GMV) in response to short-term L2 vocabulary learning. - Compare structural changes for learning with paired picture-word (PW) association versus learning within virtual environments (VE) and non-trained controls.
Lehmann et al. (2022) [158]	- To investigate whether cardiovascular exercise (CE) results in a different pattern of learning-related brain plasticity compared to non-CE controls, and how this associates with improved motor learning. - To compare the effects of a 2-week CE intervention against a non-CE control group on the learning of a dynamic balancing task over 6 weeks.
Li et al. (2018) [159]	- Evaluate whether microscopic fractional anisotropy (μFA) derived from DDE MRI can detect brain changes following cognitive training. - Evaluate training and time-related changes of DDE MRI indices (μFA, FA, and MD) and gray and white matter volume. - Test for correlation between significant imaging indices and cognitive training-induced task performance changes.
Li et al. (2022) [160]	- Compare structural connectivity (SC) and resting-state functional connectivity (rs-FC) within and between auditory and sensorimotor networks before and after musical training. - Perform correlation analysis between changes in FC or SC and practice time in the training group. - Investigate FC changes in intrinsic connectivity networks (ICNs) within auditory and motor networks and changes in auditory-motor interaction. - Examine diffusion parameter (fractional anisotropy, FA) changes in SC within auditory and motor structural networks and changes in FA of the probabilistic tract pathway between auditory and motor areas. - Explore the relationship between changes in FC and FA in the training group.
Liddell et al. (2017) [161]	- Investigate how perceptual biases affect brain activity in response to negative social cues. - Examine self-construal differences in neural responses to negative social cues, independent of cultural background.
Liu et al. (2021) [162]	- To investigate whether oxytocin (OT) modulates the neural individuation/categorization processing of racial in-group and out-group faces. - To explore how OT regulates neuronal specificity of identity and race in early face-selective regions.
Lu et al. (2021) [163]	- To unveil the interpersonal neural correlates that underlie the effect of group educational diversity on group creativity.
Martin et al. (2019) [164]	- To examine brain stimulation differences attributable to cultural background. - To investigate the effects of HD-tDCS on social cognition tasks involving self–other processing. - To understand how cultural differences impact the effects of brain stimulation on social cognition.
Martin et al. (2018) [165]	- Explore whether facilitation of dmPFC function by HD-tDCS can improve crosscultural mind-reading. - Replicate the crosscultural disadvantage on the RMET by comparing Singaporean and Caucasian students. - Determine if RMET performance in Singaporeans depends on their contact with Caucasians. - Assess if anodal HD-tDCS to the dmPFC can remove culturally mediated disadvantage in mind-reading ability in those with less contact.
Meltzer et al. (2021) [166]	- To investigate whether the benefits of bilingualism on executive function can be replicated through deliberate intervention later in life. - To compare the effects of language learning and brain training apps on executive function in older adults.
Moon et al. (2022a) [167]	- Evaluate the impact of a multidomain lifestyle intervention on regional homogeneity (ReHo) in resting-state functional brain MRI data. - Evaluate the impact of a 6-month multidomain lifestyle intervention on changes in ReHo and ALFFs of rs-fMRI using data from the SUPERBRAIN.
Moon et al. (2022b) [168]	- Evaluate the impact of a 24-week facility-based multidomain intervention (FMI) and home-based MI (HMI) on cortical thickness. - Evaluate the impact on brain volume. - Evaluate the impact on serum brain-derived neurotrophic factor (BDNF).
Moore et al. (2017) [169]	- Investigate the microstructural neuroplasticity effects of adding musical cues to a motor learning task. - Test the hypothesis that music-cued, left-handed motor training would increase fractional anisotropy (FA) in the contralateral arcuate fasciculus.
Müller et al. (2017) [170]	- To assess whether a newly designed dance training program is superior in terms of neuroplasticity compared to conventional fitness activities. - To determine if extending the training duration has additional benefits. - To investigate the potential mechanisms underlying neuroplasticity by measuring BDNF levels.
Navarro-Torres et al. (2019) [171]	- Examine how real-time cognitive control engagement influences L2 sentence comprehension, focusing on conflict adaptation. - Investigate whether cognitive control processes modulate how bilinguals experience syntactic ambiguity in their L2 compared to native speakers. - Test the hypothesis that conflict in a non-syntactic task triggers cognitive control procedures that facilitate performance in a syntactic task. - Explore how bilinguals may engage cognitive control differently due to unique demands imposed on the L2 system.
Nestor & Woodhull (2024) [172]	- Investigate the roles of group ethnicity and display rules of emotions in the neuropsychology of social cognition in Asian American and White participants.
Nijmeijer et al. (2021) [173]	- Examine the effects of a foreign language training on cognitive flexibility and its neural underpinnings, and on mental health. - Assess the unique role of foreign language training vs. other cognitive or social programs.
Nikolaidis et al. (2014) [174]	- Investigate the relationship between individual differences in training-induced changes in brain activity during a cognitive training videogame and performance changes in untrained tasks. - Test whether performance changes in an untrained working memory task can be predicted by plasticity in regions associated with working memory. - Extend previous literature on the association between training-related cognitive changes and changes in underlying neural networks.
Nilsson et al. (2018) [175]	- Investigate the effect of language training on brain structure in older adults in specific language and memory-related gray matter regions and white matter tracts. - Investigate possible predictors of achieved vocabulary proficiency in participants who completed the language training.
Ou et al. (2023) [176]	- To explore how in-group and out-group facial feedback impact different difficulty levels of implicit rule learning.
Paraskevopoulos et al. (2020) [177]	- Explore aging effects on the cortical network supporting multisensory cognition. - Define aging effects on the network’s neuroplastic attributes.
Phillips et al. (2021) [178]	- To examine whether transcutaneous auricular vagus nerve stimulation (taVNS) can facilitate L2 lexical learning for English speakers learning Mandarin Chinese over 2 days.
Pishghadam et al. (2024) [179]	- To address language learners’ needs and facilitate language learning by modifying attention and retention processes using AVE, CES, and multisensory-based instruction. - To compare the effects of AVE and CES therapies on attention and retention in L2 vocabulary learning. - To highlight the efficacy of incorporating AVE and CES treatments into conventional classroom instructions and compare them with multisensory-based instruction. - To discover modifications in attention and retention mechanisms using AVE, CES, and multisensory practices.
Powers et al. (2016) [180]	- Test whether feedback indicating the need to update social knowledge engages the ventral striatum (VS) and facilitates subsequent learning. - Examine the sensitivity of striatal signals to the value associated with social group membership. - Test whether individual differences in desire for social acceptance modulate neural responses.
Rieker et al. (2020) [181]	- Investigate the influence of explicitly cued vs. memory-based switching conditions on the set-shifting abilities of bilingual and monolingual older adults. - Investigate whether bilingualism influences age-related decline in working memory (WM).
Ripp et al. (2022) [182]	- To evaluate potential transfer effects by comprehensive cognitive testing and neuroimaging.
Schultz et al. (2024) [183]	- Assess behavioral and fMRI responses during a Stroop task in older adults before and after a language-learning intervention. - Explore the neural effects of language learning in older adults using a pre–post intervention design with fMRI. - Investigate the potential cognitive benefits of language learning, particularly in enhancing executive functions. - Determine the optimal parameters for language learning as an effective cognitive intervention for aging populations.
Shao et al. (2019) [184]	- To investigate whether motor skill learning (learning to play badminton) in adulthood influences resting-state activity in the cerebellum. - To examine the effects of motor skill learning on resting-state functions and functional connectivity associated with the cerebellum. - To assess changes in resting-state activity of specific cerebellar sub-regions (hemispheric IV-VI, VIII, and vermal VI-IX) and their intra-modular and inter-modular functional connectivity.
Sheoran et al. (2023) [185]	- Assess the effect of 12 weeks of resistance training on brain metabolism in older adults. - Quantify neurometabolite ratios in specific brain regions using proton magnetic resonance spectroscopy. - Assess time and group differences and examine associations between changes in peak torque and neurometabolite variables.
Simon et al. (2017) [186]	- To evaluate the efficacy of computerized cognitive training (CCT) focused on working memory (WM) compared to an active control condition in healthy older adults.
Sinha et al. (2020) [187]	- Investigate the effects of an aerobic exercise intervention on the dynamic rearrangement of modular community structure within the medial temporal lobe (MTL) network. - Examine how MTL network flexibility mediates the effect of exercise on mnemonic flexibility, particularly in generalizing past learning to novel task demands.
Sliwinska et al. (2021) [188]	- To test whether stimulation of the bilateral parietal region of the domain-general network impairs learning new vocabulary, indicating its causal engagement in this process.
Smith et al. (2020) [189]	- Examine TLNS-related effects on the semantic N400 brain vital sign cognitive response during cognitive skills training in healthy individuals. - Investigate whether TLNS paired with cognitive skills training significantly impacts cognitive processing, as measured by brain vital signs. - Hypothesize that TLNS paired with cognitive training over 3 days would elicit N400 changes compared with cognitive training alone.
Smith et al. (2023) [190]	- To evaluate the influence of acculturation on neuropsychological test performance in Hispanic-Americans. - To compare cognitive abilities between highly acculturated and lower acculturated Hispanic-Americans.
Struber et al. (2021) [191]	- To provide a wider understanding of adaptive learning by decoding visuomotor tasks with constant, random, or no perturbation from EEG recordings. - To separate trial-to-trial adaptation from the formation of new visuomotor mapping across trials.
Wang et al. (2015) [192]	- To test the hypothesis that modifying self-construals affects in-group bias in empathy for perceived own-race vs. other-race pain. - To measure neural responses to racial in-group and out-group members’ suffering after priming participants with interdependent or independent self-construals.
Wang et al. (2021) [193]	- To explore the BIC neural activity profiles of healthy immigrants from low-altitude regions to high-altitude regions. - To determine whether long-term exposure to high altitudes affects BIC at the behavioral and neural levels. - To reveal the behavioral performance and neural processing of BIC after long-term exposure to high altitudes by immigrants from sea-level regions.
Wanrooij et al. (2014) [194]	- Examine whether the capacity for distributional learning differs between adults and infants. - Directly compare adults’ capacity for distributional learning to that of infants. - Determine the effectiveness of distributional training of SBE /ae/,/e/ in Dutch adults. - Assess if the difference in normalized MMR amplitude between bimodally and unimodally trained participants is smaller in adults than in infants.
Wards et al. (2023) [195]	- To investigate how combined transcranial direct current stimulation and multitasking training can induce persistent gains that transfer across tasks.
Watson et al. (2024) [196]	- Elucidate the relationships between early life adversity (ELA), social learning, and empathic responding. - Understand the impact of ELA on the expression of empathy. - Understand the impact of ELA on the ability to adjust behavior after social observation.
Worschech et al. (2022) [197]	- To discover if music-driven plasticity can be observed in older adult brains. - To investigate the relationships between making music and morphological changes within auditory-related brain regions. - To explore potential relationships between cortical thickness (CT) and monaural speech-in-noise (SIN) performance.
Wu et al. (2021) [198]	To examine whether baseline integrity of three target white matter tract groups predicts task-switching improvement after 12-week Tai Chi Chuan training in middle-aged and older adults.
Xie & Ng (2024) [199]	- Investigate how bicultural switching influences cognitive performance on Executive Functions tasks among bicultural-bilinguals. - Explore the effect of cultural switching frequency on performance in tests for interference and inhibition control, set-shifting ability, and attention.
Xie & Antolović (2021) [200]	- To investigate whether the natural language immersion experience and the classroom intensive language training experience have differential impacts on cognitive control.
Yue et al. (2020) [201]	- Investigate whether long-term engagement in different types of physical exercise (Tai Chi Chuan and walking) influences resting-state brain networks differentially. - Investigate the influence of long-term Tai Chi Chuan and walking training on resting-state functional connectivity measures to understand their effects on brain function and cognitive benefits.
Zhou et al. (2022) [202]	- To investigate whether racial outgroup favoritism in neural responses to others’ pain emerges during sociocultural interactions in a new social environment.
Zink et al. (2019) [203]	- To examine the influence of acculturation on emotional learning using the EVLT-S. - To understand how word knowledge, working memory, and acculturation contribute to performance differences between Spanish and English language groups.

**Table 2:** Neural changes associated with language and cultural learning.

Category	Type of Change	Key Findings	Functional Significance
Functional Connectivity	Language Network Connectivity	Enhanced synchronization between Broca’s and Wernicke’s areas	Improved phonological processing and syntactic integration
	Executive Control Networks	Strengthened prefrontal–parietal pathways	Greater cognitive flexibility and inhibitory control
	Default Mode Network	Altered connectivity during cultural processing	Enhanced processing of culturally relevant information
	Cross-Network Integration	Increased communication between systems	More efficient coordination between cognitive domains
Structural Changes	Cortical Thickness	Increases in left inferior frontal and superior temporal regions	Greater neural resources for language processing
	Grey Matter Density	Volumetric increases in the hippocampus and temporal regions	Enhanced vocabulary acquisition and semantic storage
	White Matter Integrity	Enhanced myelination in arcuate fasciculus	Faster signal transmission between language areas
	Volumetric Changes	Hippocampal expansion proportional to vocabulary	Improved declarative memory for language learning
Activation Patterns	Processing Efficiency	Reduced BOLD response after training	More automatized language processing
	Neural Recruitment	Integration of wider networks	Ability to process complex linguistic and cultural stimuli
	Processing Mode Shift	Movement from explicit to implicit processing	The transition from controlled to automatic language use
	Cognitive Control	Enhanced conflict monitoring mechanisms	Better management of competing language systems

**Table 3:** Key brain regions and their contributions to language and cultural learning.

Brain Region	Primary Role	Changes Observed
Prefrontal Cortex	Executive control, language switching	Enhanced activation during language switching, increased cortical thickness
Temporal Lobe	Language comprehension, semantic processing	Increased grey matter density, enhanced semantic network connectivity
Hippocampus	Vocabulary acquisition, memory consolidation	Volumetric increases correlating with vocabulary size, subfield reorganization
Inferior Frontal Gyrus	Speech production, syntax	Increased cortical thickness, enhanced functional connectivity
Anterior Cingulate Cortex	Conflict monitoring, error detection	Enhanced activity during language conflict resolution, stronger connectivity
Basal Ganglia	Language selection, suppression of competing languages	Increased grey matter in caudate nucleus, enhanced control pathways
Superior Temporal Gyrus	Phonological processing	Stronger responses to non-native phonological contrasts, structural changes
Cerebellum	Procedural learning, grammar	Increased involvement in grammatical processing, enhanced connectivity

**Table 4:** Methodological approaches in neural studies of language and cultural learning.

Technique	Proportion of Studies	Primary Contribution	Key Advantages
fMRI	44%	Mapping activation patterns	High spatial resolution, whole-brain coverage
Structural MRI	25%	Identifying morphological changes	Precise volumetric and thickness measurements
DTI	19%	Assessing white matter integrity	Visualization of structural connectivity pathways
EEG	15%	Capturing temporal dynamics	High temporal resolution of neural processing
Multimodal	13%	Integrating structural and functional data	Comprehensive assessment of neural changes

## Supplementary Materials

The following supporting information can be downloaded at: https://www.mdpi.com/article/10.3390/biomimetics10060397/s1.
