# Supplementary material for: Brain-Inspired Multisensory Learning: A Systematic Review of Neuroplasticity and Cognitive Outcomes in Adult Multicultural and Second Language Acquisition
Source: Biomimetics (Basel). 2025 Jun 12;10(6):397. doi: 10.3390/biomimetics10060397 (PMC12190708; doi:10.3390/biomimetics10060397)
Supplement: Supplementary file 1 [file biomimetics-10-00397-s001.zip › Sup_Table_S1_Systematic_Review.pdf]

Table S1. Research Articles of Systematic Analysis (n=80).

Authors	Study objectives	Participants	Methodology	Main findings	Intervention
Acevedo et al. (2022) [124]	- Examine the effects of a 4-week cognitive training program with neurofeedback (CT-NF) on cognitive function in older adults.<br>- Compare the cognitive improvements between a treatment group using app-based ABC games and a control group using Tetris.<br>- Test the hypothesis that CT-NF with ABC exercises results in greater cognitive improvements than Tetris.<br>- Investigate whether the strength of right prefrontal cortex activity during CT-NF is associated with cognitive function and game performance.	86	- Randomized controlled trial with 86 participants.<br>- Participants divided into treatment (ABC games) and control (Tetris) groups.<br>- 4-week intervention with cognitive training and neurofeedback (CT-NF).<br>- Daily training for 10-15 minutes, 5 to 7 days per week.<br>- Cognitive assessments conducted pre- and post-intervention.<br>- Cortical brain activity measured using fNIRS.<br>- Virtual training session provided for device and application use.<br>- Compliance monitored by tracking training days.<br>- Controlled for age and number of training days in analyses.	The study concludes that a 4-week CT-NF program with ABC exercises enhances cognition in older adults, particularly in memory, verbal memory, processing speed, and executive function, while also highlighting the predictive value of brain activity for cognitive performance.	- Type of cognitive training program (ABC games vs. Tetris)<br>- Duration of the intervention (4 weeks)
Alain et al. (2019) [125]	- Assess the effect and maintenance of 3-month music and visual art training programs on neuroelectric brain activity in older adults.<br>- Compare cognitive functioning improvements between music, visual art, and control groups.<br>- Measure maintenance of effects with a 3-month follow-up.<br>- Evaluate training-related neuroplastic changes in sensory processing and executive functions using ERPs.	60	- Partially randomized intervention design with three groups: music training, visual art training, and passive control.<br>- 3-month training program with follow-up assessments 3 months post-training.<br>- High-density EEG used to measure neuroelectric brain activity during auditory oddball paradigms and visual GoNoGo task.<br>- Participants underwent 36 one-hour sessions over three months.<br>- Neuropsychological assessments conducted pre-, post-, and follow-up to evaluate cognitive functioning.<br>- EEG data analyzed for changes in auditory evoked responses and visual processing.	The study demonstrates that short-term music and visual art training can lead to neuroplastic changes and improvements in cognitive functions in older adults, suggesting these programs as effective interventions for enhancing brain plasticity.	- Type of training program (music training, visual art training)<br>- Presence of a control group (no-contact control)
Alotaibi et al. (2023) [126]	- To track neural changes over three days of learning Arabic phonetic categorization using fMRI and DTI.	20	- Combined fMRI and DTI study<br>- Tracks neural changes over three days of Arabic phonetic categorization learning<br>- Participants: 20 adult native English speakers<br>- Scans conducted before and after training<br>- Training: Perception and production of phonetic contrasts for one hour on three consecutive days	Three hours of phonetic categorization learning can induce functional and microstructural neural changes typically associated with long-term learning.	- Language being learned (Arabic)<br>- Time (before and after training)<br>- Training duration (one hour on three consecutive days)

			- Control language: Chinese<br>- Measures: Behavioral performance, fMRI signal changes, FA value changes, functional connectivity changes		- Control language (Chinese)
Alwashmi et al. (2023) [127]	- Investigate how functional brain changes support behavioural performance improvements during an audio-visual (AV) learning task.	20	- Participants: 20 healthy individuals<br>- Training: 30 min daily VR training for four weeks<br>- Task: Audio-visual adaptation of a 'scanning training' paradigm<br>- Data Collection: fMRI and performance data at baseline, after two and four weeks of training, and four weeks post-training<br>- Additional Tests: Conducted in a controlled laboratory environment to assess transfer of performance gains	The study concludes that VR-based multisensory training enhances cognitive function and performance through augmented brain activation, with potential applications in rehabilitation.	- Duration of VR training (30 minutes daily)<br>- Length of training period (four weeks)<br>- Type of task (AV adaptation of a 'scanning training' paradigm)<br>- Conditions (visual-only and AV conditions)<br>- Timing of data collection (baseline, after two and four weeks of training, and four weeks post-training)
Bae et al. (2020) [128]	- Examine cortical thickness changes associated with a multicomponent exercise intervention combining physical exercise and cognitive training in older adults with cognitive decline.<br>- Investigate whether the intervention increases cortical thickness in the frontal and temporal regions over a 10-month period.<br>- Determine if changes in cortical thickness are positively associated with changes in cognitive performance.	280	- Secondary analysis of a randomized controlled trial with 280 older adults with cognitive decline.<br>- Random assignment to multicomponent exercise group or health education control group.<br>- Intervention: Weekly 90-minute sessions of aerobic exercise, dual-task training, muscle strength, and balance training for 10 months.<br>- Control: Three health education classes during the study period.<br>- Cortical thickness measured using FreeSurfer software.<br>- Cognitive performance assessed using NCGG-FAT.<br>- Statistical analyses: t-tests, Chi-square tests, repeated-measures mixed model, Pearson's correlation, linear regression.	A 10-month multicomponent exercise program combining aerobic exercise and cognitive training effectively increases cortical thickness in the temporal lobe and is associated with improved cognitive performance in older adults with cognitive decline.	- Type of intervention (multicomponent exercise vs. health education control)<br>- Time (baseline and 10-month follow-up)<br>- Interaction between group assignment and time
Balboa-Bandeira et al.	- To explore the effects of transcranial random noise stimulation (tRNS) on verbal fluency in healthy multilingual individuals.	50	- Aim: Explore effects of tRNS on verbal fluency in multilingual individuals.<br>- Participants: 50 healthy multilingual adults.	tRNS on the left prefrontal cortex may enhance phonemic but not	- Type of stimulation (tRNS vs. sham)

(2024) [129]			- Design: Randomized controlled trial with tRNS and sham groups. - Stimulation: Electrodes on left dorsolateral prefrontal cortex and left inferior frontal gyrus. - Tasks: Phonemic and semantic verbal fluency tasks in three languages. - Timing: Tasks performed before, during, and after stimulation.	semantic fluency in healthy multilingual adults.	
Bastarrika & Davidson (2017) [130]	- Investigate how Spanish adult learners of Basque respond to morphosyntactic violations after short training. - Characterize brain areas involved in recognizing grammatical constraints using MEG. - Test the hypothesis that cognate vocabulary facilitates rapid incorporation of grammatical rules. - Predict that L1 brain networks are engaged during L2 learning and similar areas will show responses in L2 as in L1.	17	- Magnetoencephalography (MEG) was used to measure brain responses. - Participants: 17 native Spanish speakers aged 20-29. - Study phases: pretest, training, generalization-test. - Feedback provided only during training. - Conducted over two consecutive days with multiple blocks. - Behavioral data analyzed using multilevel generalized linear regression. - MEG data processed with Fieldtrip toolbox and specific filtering techniques.	The study concludes that rapid changes in neural responses during second language acquisition can lead to native-like processing patterns within hours, suggesting that models of L2 learning should account for these quick adaptations.	- Phases of the experiment (pretest, training, generalization-test) - Type of noun-phrases (control phrases, violation phrases, fillers) - Feedback provision (feedback during training blocks) - Experimental sessions (two sessions on consecutive days) - Type of tasks (comprehension and production)
Belleville et al. (2014) [131]	- Measure the neural substrates as a function of whether divided attentional training programs induce the use of alternative processes or rely on repeated practice. - Determine whether different patterns of brain activation in older adults can result from repeated practice or strategic training.	48	- Randomized controlled trial with 48 older adults. - Participants assigned to one of three training programs: SINGLE REPEATED, DIVIDED FIXED, DIVIDED VARIABLE. - Training involved alphanumeric equation and visual detection tasks. - Brain activation measured using fMRI pre- and post-training. - Tasks performed under single-task and dual-task conditions. - E-prime software used for task implementation; responses recorded with fiber optic response pads. - Data analyzed using MATLAB and SPM8; correlational analyses conducted.	The study found that the aging brain is highly plastic and responds differently to various attentional training programs, with the type of training being a critical factor in determining the pattern of brain activation.	- Training program (SINGLE REPEATED, DIVIDED FIXED, DIVIDED VARIABLE) - Time (Pre, Post)

Berlingerie et al. (2016) [132]	To address the neurofunctional signatures underlying both the DEAR effect and the manifestation of politically correct behaviors.		- Participants: Caucasian individuals<br>- Method: Functional magnetic resonance imaging (fMRI)<br>- Stimuli: Videos of African or Caucasian actors being touched by a rubber eraser or a needle<br>- Procedure:<br>- Stimulus phase: Participants instructed to empathize with actors<br>- Response phase: Participants judged the pain level experienced by actors<br>- Measurement: Implicit racial bias measured by the Implicit Association Test (IAT)	The study discusses how racial biases and politically correct behaviors manifest in brain activity and behavior, proposing a model that integrates these contrasting forces at neurobiological and behavioral levels.	- Race of the actors (African or Caucasian)<br>- Type of object used (rubber eraser or needle)
Brusa et al. (2021) [133]	- Detect the activation of implicit stereotypical representations associated with other-race people using EEG/ERPs.<br>- Investigate the modulation of these stereotypical representations through the presentation of positive versus neutral social information.<br>- Explore whether exposure to positive media-driven information can modulate racial prejudice, as indicated by changes in the N400 response.	40	- EEG/ERPs were used to measure implicit stereotypical representations.<br>- 40 Italian Caucasian participants were involved, unaware of the study's purpose.<br>- Participants were exposed to 285 sentences related to racial stereotypes.<br>- Prior to the task, participants watched a 10-minute video (positive for experimental group, neutral for control group).<br>- EEG signals were recorded during a sentence reading task.<br>- A fictitious task involving rare animal names was used as a distraction.	The study demonstrates that exposure to positive media-driven information about other-race individuals can modulate pre-existing racial prejudice, as indicated by changes in the N400 response, and suggests further research to explore the duration and context of this effect.	- Type of video documentary (positive social information vs. neutral)
Bubbico et al. (2019) [134]	- Analyze the effects of a 4-month second language learning program on functional changes in the brain of healthy elderly individuals.<br>- Assess the effects on cognitive status using a comprehensive neuropsychological battery.<br>- Measure changes in brain functional connectivity using resting-state functional magnetic resonance imaging (rs-fMRI).<br>- Investigate neuroplastic-related effects of second language learning in terms of cognitive and brain networks functional connectivity changes.	30	- A 4-month second language learning program with 16 sessions of 2 hours each.<br>- Participants: 14 in the intervention group and 12 in the control group.<br>- Neuropsychological assessments and rs-fMRI conducted pre-and post-intervention.<br>- Intervention: Group lessons with a native English teacher.<br>- Control group maintained daily habits without training.<br>- Statistical analysis: General linear model, P-value < 0.05 for significance.<br>- Imaging: Philips Achieva 3 Tesla scanner, analyzed with linear mixed effects model in R.	The study demonstrates that a 4-month second language learning program can lead to functional reorganization in the brain and improvements in global cognition in elderly individuals, supporting the potential of such programs as non-pharmacological interventions to counteract cognitive aging and delay dementia.	Second language learning program (4 months English course)
Bugos et al. (2022) [135]	- To examine training-related changes in auditory-evoked oscillatory activity in healthy older adults using time-frequency analyses.	50	- Participants: 50 healthy older adults divided into three groups (music training, visual art training, no-treatment control).<br>- Method: Time-frequency analyses of auditory-evoked oscillatory	Music and visual art training in older adults lead to sustained neuroplastic changes in auditory	- Type of training program (music training, visual art

			activity.<br>- Task: Oddball auditory paradigms with synthesized piano tones or vowels.<br>- Data Collection: High-density EEG.<br>- Time Points: Pre-training, post-training, and three-month follow-up.<br>- Duration: 12-week training programs.	processing, enhancing cognitive functions such as memory.	training, no-treatment control)<br>- Time points of measurement (pre-training, post-training, three-month follow-up)
Chen et al. (2015) [136]	- To examine whether neural responses in the ventral striatum to in-group facial expressions can predict friendship patterns in newly arrived individuals from China 6 months later.<br>- To understand why some new arrivals primarily favor in-group over out-group friendships.<br>- To test the hypothesis that VS activity for in-group compared to out-group happy expressions predicts new arrivals' relative percentage of in-group friends.	27	- Participants: 27 newly arrived Chinese international graduate students.<br>- Stimuli: In-group (Chinese) and out-group (Caucasian) faces with various emotional expressions.<br>- Pilot test: Conducted to ensure similar emotional intensity across face stimuli.<br>- Paradigm: Backward masking to present emotional expressions without explicit awareness.<br>- Imaging: Functional MRI used to measure brain activity during viewing of masked faces.<br>- Analysis: Contrast images and ANOVA used to analyze brain responses.<br>- Outcome measure: Changes in percentages of in-group and out-group friends on social networking services.	The study found that greater reward reactivity to in-group happy expressions predicted friendship patterns, highlighting the potential of using neural responses to predict real-world behaviors and supporting the concept of in-group favoritism.	- VS parameter estimate difference scores comparing in-group to out-group happy expressions<br>- VS parameter estimate difference scores comparing in-group to out-group fearful expressions<br>- VS parameter estimate difference scores comparing in-group to out-group surprised expressions<br>- Extraversion scores<br>- Openness scores
Choi et al. (2020) [137]	- Examine gender variations in the effect of education and acculturation on cognitive function in a group of older immigrants.	2061	- Data source: Study of Older Korean Americans, a multistate survey<br>- Population: Korean Americans aged 60 years and older (N = 2061)<br>- Analysis method: Multivariate linear regression models<br>- Cognitive function measurement: Mini-Mental State Examination<br>- Focus: Independent and interactive effects of education, acculturation, and gender	Gender significantly influences the cognitive health benefits derived from education and acculturation, with women experiencing greater benefits than men, especially in low acculturation contexts.	- Education<br>- Acculturation
Colflesh et al. (2016) [138]	- To examine the effects of working memory training on working memory capacity.<br>- To examine the effects of working memory		- Examined effects of working memory training on working memory capacity and second language ability.<br>- Participants: Adult learners of Spanish.<br>- Training tasks used Spanish words and sentences as stimuli.	The study found that while working memory training did not improve working memory capacity, it helped learners	- Working memory training<br>- Group assignment (training vs. control)

	training on second language ability in adult learners of Spanish.		- Included a control group for comparison.<br>- Outcome measures: Working memory assessments and a Spanish self-paced reading task.	exhibit more native-like language processing patterns, suggesting benefits in coping with language learning demands.	
Deng et al. (2018) [139]	- To explore the neuroplasticity induced by training on non-native pitch patterns.<br>- To investigate the effects of multi-talker versus single-talker training conditions on brain activation and functional connectivity.<br>- To understand the neural mechanisms involved in voice processing and lexical phonology accessing during multi-talker training.<br>- To correlate neural changes with learning success in multi-talker training.	32	- Participants: Native English speakers<br>- Training: Non-native pitch patterns (Mandarin Chinese tones)<br>- Groups: Multi-talker (N = 16) and single-talker (N = 16) training conditions<br>- Neuroimaging: Functional magnetic resonance imaging (fMRI)<br>- Brain Regions: Anterior right superior temporal gyrus (aRSTG) and posterior left superior temporal gyrus (pLSTG)<br>- Task: Tone identification task conducted before and after training	The study concludes that multi-talker training enhances neural efficiency and cooperation between brain areas, influencing the processing of newly learned speech patterns.	- Training condition (multi-talker vs. single-talker)
Du et al. (2023) [140]	To examine what experience other than immersion may help adult learners read with native-like neural responses.	25	- Randomized controlled trial design<br>- Participants: 13 native Chinese English learners in English letter-sound association training, 12 in visual symbol-sound association training, and a reference group of native English readers<br>- Interventions: English letter-sound association training and visual symbol-sound association training<br>- Duration: Six hours of training with observations at three and six hours<br>- Measurements: Neural responses including cross-modal mismatch negativity (MMN) and theta oscillations	With specific training in critical second language reading skills, adult learners can achieve native-like neural responses, overcoming native language constraints.	- Type of training (English letter-sound association vs. visual symbol-sound association)<br>- Duration of training (three hours vs. six hours)
Emch et al. (2019) [141]	- Investigate behavioral changes following an adaptive online verbal WM training in healthy middle-aged adults.<br>- Investigate neural changes following an adaptive online verbal WM training in healthy middle-aged adults.<br>- Provide evidence for neural plasticity and/or improvement in behavioral performance in this age group.	63	- Used functional magnetic resonance imaging (fMRI) to measure brain plasticity.<br>- Conducted an 8-week adaptive online verbal working memory training with 32 sessions.<br>- Participants were pseudo-randomly assigned to experimental and active control groups.<br>- Employed an adaptive n-back task for the experimental group and a fixed low-level task for the control group.<br>- Assessed verbal working memory using the HAWIE-R digit span test before and after training.	The study demonstrates that adaptive online verbal working memory training can enhance neural efficiency and improve performance in middle-aged adults, supporting the potential for lifelong cognitive improvement.	- Group assignment (experimental vs. control)<br>- Type of training (adaptive online verbal WM training vs. low-level vWM training)<br>- Number of training sessions (32 sessions

			- Analyzed data using JASP, IBM SPSS, and SPM12 in MATLAB.<br>- Imaging protocol included T1 MPRAGE, T2, FLAIR, DTI, EPI resting state, task-fMRI, and FDG-PET sequences.		over 8 weeks)<br>- Demographic variables (age, gender, years of education)
Farah et al. (2021) [142]	- To determine if early exposure to cognitive and linguistic stimulation impacts brain structure.<br>- To investigate whether genetic predispositions account for the co-occurrence of certain neuroanatomical phenotypes and a tendency to engage children in cognitively stimulating activities.		- Randomized controlled trial<br>- Participants: Low socioeconomic status infants<br>- Intervention: 5 years of cognitively and linguistically stimulating center-based care<br>- Comparison: Control group with a different condition<br>- Outcome: Changes in brain structure measured in midlife	The study demonstrates that early cognitive and linguistic stimulation leads to significant changes in adult brain structure, particularly in males, extending findings from animal studies to humans and highlighting the role of linguistic stimulation.	- Type of early cognitive experience (cognitively and linguistically stimulating center-based care vs. comparison condition)
Gavett et al. (2018) [143]	- Examine longitudinal associations between structural MRI and cognition in a diverse sample.<br>- Investigate whether and how the associations between brain variables and cognitive change differ across ethnoracial groups.	444	- Diverse sample of older adults from the UCD Aging Diversity Cohort.<br>- Participants underwent multiple annual study visits with cognitive evaluations and MRI scans.<br>- MRI measures included gray matter volume, hippocampal volume, and white matter hyperintensity.<br>- Cognitive outcomes measured using SENAS.<br>- Multilevel latent variable modeling to test ethnoracial differences in brain-cognition associations.<br>- Mixed effects regression analyses for cognitive trajectories.<br>- Multiple group models to evaluate ethnoracial differences in brain effects on cognition.	The study found that the associations between changes in brain structure and cognitive decline differ across ethnoracial groups, with Alzheimer's disease having a greater influence in Whites, global gray matter atrophy being the strongest contributor in African Americans, and baseline white matter hyperintensities being the strongest predictor in Hispanics.	- Baseline lobar MRI volumes<br>- Change in lobar MRI volumes<br>- Global gray matter volumes (baseline and change)<br>- Age<br>- Education<br>- Gender<br>- Ethnoracial group<br>- Language of test administration<br>- APOE ε4 status
Grossmann et al. (2023) [144]	- To examine whether learning a foreign language can improve executive attention and executive functions in healthy older adults.<br>- To identify factors affecting cognitive change in foreign language learners, such as cognitive reserve, previous foreign knowledge and usage, and global cognition at baseline.	54	- Randomized controlled trial design with two parallel groups: language learning group and waiting list control group.<br>- Participants: 34 monolinguals aged 65-80 years.<br>- Intervention: Spanish course for beginners, 1.5 hours per day, five days a week for three weeks.<br>- Assessments: Conducted before, immediately after, and three months post-intervention.<br>- Primary outcome: Executive attention assessed by Stroop Interference Test and Divided Attention subtest.	Studying a foreign language does not generally improve executive attention or executive functioning, but individuals with poorer baseline cognition may benefit in response inhibition, highlighting the need for targeted dementia prevention efforts and more	- Group assignment (language learning group vs. waiting list control group)<br>- Cognitive reserve (CR)<br>- Previous foreign language knowledge and usage

			- Secondary outcomes: Executive functions including inhibition, shifting, and updating.<br>- Recruitment: Via advertisements; inclusion criteria included age and language proficiency.<br>- Data analysis: Conducted using IBM SPSS Statistics 26.	individualized learning approaches.	- Baseline level of general cognition
Grossmann et al. (2021) [145]	- Investigate the effects of short and intensive foreign language learning on executive functions in healthy older adults.	60	- Randomised controlled trial design<br>- Participants: 60 native German-speaking monolingual healthy older adults, aged 65–80 years<br>- Groups: Foreign language learning group and waiting list control group<br>- Intervention: Face-to-face, group-based Spanish course for beginners, 1.5 hours/day, 5 days/week, for 3 weeks<br>- Assessments: Cognitive performance in executive functions assessed before and after intervention, and after a 3-month follow-up<br>- Control group receives Spanish lessons after final assessment	The study aims to determine if short and intensive foreign language learning can enhance executive functions and promote healthy cognitive aging in older adults.	- Foreign language learning (presence or absence of the intervention)<br>- Duration and intensity of the language course (1.5 hours per day, 5 days a week, for 3 weeks)
Hu et al. (2015) [146]	- Investigate cultural differences in learning with social feedback between Chinese and Caucasian subjects.<br>- Examine the effect of oxytocin (OXT) on facilitating learning with social feedback in Chinese subjects.<br>- Explore the neural substrates and functional connectivity associated with OXT's effects on learning using fMRI.	151	- Two independent double-blind placebo-controlled experiments.<br>- Participants: Male Chinese subjects.<br>- Administration of intranasal oxytocin (24IU) or placebo.<br>- Use of Reinforcement Association Learning Task (RALT) with social and non-social feedback.<br>- Experiment 3 included fMRI to measure brain activity.<br>- fMRI data analyzed using SPM8 software; behavioral data analyzed using three-way ANOVA.	The study demonstrates cultural differences in learning with social feedback, with Chinese subjects performing worse than Caucasians, but oxytocin selectively enhances learning with social feedback in both groups by increasing activity in emotional memory and reward processing brain regions.	- Administration of oxytocin (OXT) vs. placebo (PLC)<br>- Type of feedback (social vs. non-social)<br>- Gender of face used in social feedback (male vs. female)
Jiang et al. (2016) [147]	- Investigate whether changes in cortical thickness correlate with cognitive function changes in healthy older adults after cognitive training interventions.<br>- Examine the differential impacts of multi-domain and single-domain cognitive training interventions.	48	- Longitudinal MRI scanning to measure cortical thickness using FreeSurfer Software.<br>- Cognitive assessments using RBANS at baseline and 12-month follow-up.<br>- Participants aged 65–75 received cognitive training interventions in small groups.<br>- Multi-domain training included memory, reasoning, problem-solving, and physical activities.<br>- Single-domain training focused on reasoning tasks.<br>- Training consisted of 24 sessions over 12 weeks, with booster	The study found that cortical thickness changes were associated with cognitive function changes in healthy elderly after cognitive training, with multi-domain training offering more benefits than single-domain training, particularly in visuospatial/constructional,	- Type of cognitive training intervention (multi-domain vs. single-domain)<br>- Duration and frequency of the intervention (24 sessions, 60 minutes each, over 12 weeks)<br>- Specific activities

			sessions monthly from 6 to 9 months post-intervention. - Statistical analysis involved repeated-measures ANOVA and partial correlation analysis.	attention, and delayed memory abilities.	included in the training programs - Timing of assessments (baseline and 12-month follow-up)
Jünemann et al. (2023) [148]	- To investigate whether learning to play the piano can counteract or slow down age-related cognitive decline. - To examine changes in resting-state functional connectivity (FC) as a result of piano training. - To compare the effects of piano playing with music listening/musical culture lessons on FC in healthy older adults.	109	- Comparative study design with two groups: piano training vs. music listening/musical culture lessons. - Sample size: 109 healthy older adults. - Use of resting-state functional magnetic resonance imaging (fMRI) to measure functional connectivity. - Longitudinal measurements at three time points: baseline, 6 months, and 12 months.	Piano training in healthy older adults induces functional neuroplasticity, increasing network efficiency for auditory-motor integration and potentially counteracting age-related cognitive decline.	- Type of intervention (learning to play the piano vs. music listening/musical culture lessons) - Time (baseline, 6 months, 12 months)
Jünemann et al. (2022) [149]	- Compare the influence of six months of piano training versus music listening/musical culture lessons on white matter properties in elderly individuals. - Use fixel-based analysis to investigate white matter microscopic and macroscopic changes induced by musical training. - Anticipate less decline or an increase in white matter microstructure and/or macrostructure through piano lessons. - Correlate neuronal changes with behavioral changes and determine their relationship to training intensity.	155	- Randomized controlled trial with two groups: piano training (PP) and music listening/musical culture (MC). - Participants: 121 musically naïve healthy elderly individuals. - Duration: 6 months of weekly 60-minute lessons and daily 30-minute homework. - Neuroimaging: Diffusion-weighted MRI on 3.0 T Siemens scanners. - Data preprocessing: Denoising, distortion correction using MRtrix3 and FSL. - Analysis: Fixel-based analysis of white matter tracts. - Behavioral assessment: Rey Auditory Verbal Learning Test for episodic memory. - Statistical analysis: General Linear Model with difference scores for group comparisons.	Learning to play the piano stabilizes white matter microstructure in the fornix in healthy elderly individuals, with changes related to training intensity and positively correlated with episodic memory performance.	- Type of intervention (piano training vs. music listening/musical culture lessons) - Duration of intervention (six months) - Frequency of lessons (60 minutes once a week) - Amount of homework (at least 30 minutes each day)
Katsumi et al. (2020) [150]	- To investigate the neural mechanisms associated with the perception and evaluation of nonverbal behaviors displayed by racial in-group versus out-group members.	42	- 42 white participants were involved. - Electroencephalographic (EEG) recording was used. - Participants observed avatars displaying dynamic nonverbal behaviors (approach and avoidance poses and expressions). - Participants rated the avatars after observation. - Event-related potentials (ERPs) analysis focused on N450 and late positivity components.	The study highlights neural mechanisms underlying racial in-group bias in social cognition, showing that differential neural sensitivity to nonverbal cues is linked to in-group bias and has	- Type of nonverbal behavior (approach vs. avoidance) - Racial group of avatars (in-group vs. out-group)

			- Time–frequency analysis identified suppression of beta-range power.	implications for interactions with diverse racial backgrounds.	
Kim et al. (2017) [151]	- Investigate the changes in cognitive functions and brain activation after multicomponent training of cognitive control in non-demented older adults.	27	- Developed and implemented a computerized Multicomponent Training of Cognitive Control (MTCC) with task variability and adaptive procedures.<br>- Participants: 27 community-dwelling adults aged 64-77 years, divided into a training group (14) and a control group (13).<br>- Duration: 8 weeks of MTCC for the training group; no training for the control group.<br>- Assessments: Neuropsychological tests and fMRI conducted before and after the training.	The study concludes that multicomponent training of cognitive control improves cognitive functions and brain activation in older adults, but further research is needed to confirm these findings.	- Multicomponent Training of Cognitive Control (MTCC)<br>- Duration of training (8 weeks)
Kleemeyer et al. (2017) [152]	- Investigate whether exercise-induced fitness improvements are associated with enhanced neural specificity in older adults.	52	- Participants: 52 older adults aged 59–74 years.<br>- Random assignment to two aerobic-fitness training regimens (low-intensity and high-intensity).<br>- Training: Stationary bicycle exercise, three times a week for six months.<br>- Fitness Assessment: Graded maximal exercise test measuring VO2 max and VO2 AT.<br>- Neural Assessment: Functional MRI experiments before and after training.<br>- Data Analysis: Multivariate pattern analysis (MVPA) and General Linear Model (GLM) for neural activation patterns.	Exercise-induced fitness improvements are positively associated with enhanced neural specificity, suggesting that regular physical activity may mitigate age-related declines in neural specificity and contribute to cognitive benefits.	- Type of aerobic-fitness training regimen (high-intensity vs. low-intensity)
Kommula et al. (2023) [153]	- Determine the effect of acute exercise, compared to a seated rest control condition, on pleasant and unpleasant emotion-related regional activation in healthy older adults.<br>- Assess the impact of acute exercise on pleasant and unpleasant emotion-related network recruitment using task-related functional MRI.<br>- Determine whether exercise-related changes in brain activation are correlated with pre-to post-condition changes in self-reported affect.	32	- Participants: 32 active older adults.<br>- Design: Within-subjects, counterbalanced order across two testing days.<br>- Conditions: 30 minutes of moderate to vigorous cycling and 30 minutes of seated rest.<br>- Measurements: fMRI data acquisition, PANAS for affect, heart rate, and RPE.<br>- Stimuli: International Affective Pictures System (IAPS) images.<br>- Analysis: Meta-analysis informed ROI analysis, meta-analysis informed mask analysis, exploratory functional ROI analysis.	The study suggests that acute exercise influences brain activation related to emotional processing in older adults, supporting improved emotional reactivity and positive affect, and highlights the potential of exercise as a lifestyle intervention for mental health, though long-term effects remain to be explored.	- Acute exercise (30 minutes of moderate to vigorous intensity cycling)<br>- Seated rest (30 minutes of wakeful seated chair rest)
Koschutnig et al.	- Investigate changes in white matter morphology following complex motor learning (slackline		- Participants: Young adults from the general population<br>- Intervention: Learning to walk a slackline	The study provides substantial new evidence that learning a	- Slackline skill acquisition

(2024) [154]	walking)<br>- Provide evidence on how learning a complex motor skill modulates fiber organization and density in sensorimotor tracts		- Imaging: Brain imaging conducted before intervention, after learning, and after follow-up<br>- Measures: Voxel-based and fixel-based analyses for micro- and macrostructural characteristics of WM fiber tracts<br>- Control Group: Randomly assigned, scanned at the same time points without intervention<br>- Analyses: Whole brain fixel-based analyses and NODDI parameters	complex motor skill modulates fiber organization and density in sensorimotor tracts.	(intervention)<br>- Time points of assessment (before intervention, after learning, follow-up)
Lamar et al. (2014) [155]	- Investigate the modulatory effect of serotonin using acute tryptophan depletion (ATD) during a cognitive switching task.<br>- Test whether ATD is associated with an anterior-to-posterior shift in brain activation during the switching task in older adults.	10	- Ten healthy women over 55 years participated in a within-group double-blind sham-controlled crossover study.<br>- Participants underwent two testing sessions: one with acute tryptophan depletion (ATD) and one with a sham condition.<br>- A rapid mixed trial event-related fMRI design was used to measure brain activity.<br>- Participants consumed a tryptophan depletion mixture or a sham mixture before testing.<br>- fMRI data were collected using a 1.5 T scanner.<br>- Image preprocessing and analysis were conducted using X-BAM v4.0 software.<br>- Data analysis included first-level and second-level analyses with transformation into Talairach space.	In older adults, acute tryptophan depletion causes an anterior-to-posterior shift in brain activation during task switching, suggesting a compensatory mechanism to maintain performance despite reduced prefrontal activation.	- Acute tryptophan depletion (ATD)<br>- Sham depletion condition
Lawlor-Savage et al. (2019) [156]	- To determine the cognitive benefits of adaptive working memory training in healthy adults.<br>- To identify biological changes present after working memory training using structural neuroimaging.<br>- To compare the effects of n-back working memory training and processing speed training on neuroanatomical metrics.	48	- Participants: 24 healthy adults aged 18-40 years<br>- Intervention: 6-week n-back working memory training program<br>- Control: Active control group with 6-week processing speed training<br>- Measurements: Structural MRI scans before and after training<br>- Neuroanatomical focus: Cortical surface area, thickness, volume in frontal and parietal lobes; subcortical and total gray matter volumes<br>- Statistical analysis: Group by time repeated measures ANOVAs	The study concludes that adaptive n-back working memory training does not induce neuroanatomical changes, and further research into other training forms is needed to advance the field.	- Training type (n-back working memory training vs. processing speed training)<br>- Time (before and after the 6-week training period)
Legault et al. (2019) [157]	- Examine changes in cortical thickness (CT) and gray matter volume (GMV) in response to short-term L2 vocabulary learning.<br>- Compare structural changes for learning with paired picture-word (PW) association versus learning within virtual environments (VE) and non-trained controls.		- Examined changes in cortical thickness (CT) and gray matter volume (GMV) using neuroimaging.<br>- Compared two learning methods: paired picture-word (PW) association and virtual environments (VE).<br>- Included a non-trained control group.<br>- Participants learned 90 Mandarin Chinese nouns over 7 sessions in approximately 20 days.	The study concludes that short-term second language training leads to structural brain changes, which vary based on learning contexts and individual cognitive differences.	- Learning context (paired picture-word association vs. virtual environments)<br>- Presence or absence of training (trained vs. non-trained controls)

					- Duration of training (7 sessions over approximately 20 days)
Lehmann et al. (2022) [158]	- To investigate whether cardiovascular exercise (CE) results in a different pattern of learning-related brain plasticity compared to non-CE controls, and how this associates with improved motor learning.<br>- To compare the effects of a 2-week CE intervention against a non-CE control group on the learning of a dynamic balancing task over 6 weeks.		- Randomized controlled trial with healthy human participants<br>- Comparison of a 2-week cardiovascular exercise (CE) intervention against a non-CE control group<br>- Subsequent learning of a dynamic balancing task (DBT) over 6 weeks<br>- Structural and functional MRI measurements conducted at regular 2-week intervals	The study concludes that cardiovascular exercise leads to distinct patterns of brain plasticity that correlate with improved motor learning, enhancing our understanding of the neural mechanisms linking exercise and learning.	- Cardiovascular exercise (CE) intervention (presence vs. absence)<br>- Time (2-week intervals for MRI measurements)
Li et al. (2018) [159]	- Evaluate whether microscopic fractional anisotropy (μFA) derived from DDE MRI can detect brain changes following cognitive training.<br>- Evaluate training and time-related changes of DDE MRI indices (μFA, FA, and MD) and gray and white matter volume.<br>- Test for correlation between significant imaging indices and cognitive training-induced task performance changes.	29	- Prospective study design<br>- Random assignment of 29 healthy volunteers into training (n = 21) and control (n = 8) groups<br>- Use of double diffusion encoding (DDE) MRI and 3D-T1-weighted imaging<br>- Cognitive training with dual N-back and attention network tasks, five days per week for four weeks<br>- Evaluation of DDE MRI indices (μFA, FA, MD) and gray/white matter volume using mixed-design ANOVA<br>- Partial correlation analyses to test correlations between imaging indices and task performance changes	Microscopic fractional anisotropy (μFA) can serve as a sensitive index to detect neuroplastic changes induced by cognitive training.	- Cognitive training (presence or absence)<br>- Group assignment (training group vs. control group)<br>- Time (interval of 4–6 weeks)
Li et al. (2022) [160]	- Compare structural connectivity (SC) and resting-state functional connectivity (rs-FC) within and between auditory and sensorimotor networks before and after musical training.<br>- Perform correlation analysis between changes in FC or SC and practice time in the training group.<br>- Investigate FC changes in intrinsic connectivity networks (ICNs) within auditory and motor networks and changes in auditory-motor interaction.<br>- Examine diffusion parameter (fractional anisotropy, FA) changes in SC within auditory and	60	- Participants: 29 novice healthy young adults in the training group and 27 in the control group.<br>- Intervention: Musical training program including professional instruction and practice.<br>- Measurements: Structural connectivity (SC) and resting-state functional connectivity (rs-FC) using neuroimaging techniques.<br>- Analysis: Mixed ANOVA for group effects, correlation analysis for practice time effects, and probabilistic fiber tracking for SC assessment.	Musical training enhances functional connectivity within sensorimotor regions and increases both functional and structural connectivity between auditory and motor networks, with stronger effects observed in those with longer practice durations.	- Musical training<br>- Time<br>- Group (training group vs. control group)

	motor structural networks and changes in FA of the probabilistic tract pathway between auditory and motor areas.<br>- Explore the relationship between changes in FC and FA in the training group.				
Liddell et al. (2017) [161]	- Investigate how perceptual biases affect brain activity in response to negative social cues.<br>- Examine self-construal differences in neural responses to negative social cues, independent of cultural background.	35	- Participants: Healthy individuals high in collectivistic (n=16) and individualistic (n=19) self-construal.<br>- Task: Negative social cue evaluation task.<br>- Measurement: fMRI scanning to observe neural processes.<br>- Analysis: Between-group analyses of brain activity.	The study concludes that individual differences in self-construal significantly influence neural responses to negative social cues, serving as an important framework for perceptual processes beyond cultural group comparisons.	- Self-construal type (collectivistic vs. individualistic)<br>- Negative social cue evaluation task
Liu et al. (2021) [162]	- To investigate whether oxytocin (OT) modulates the neural individuation/categorization processing of racial in-group and out-group faces.<br>- To explore how OT regulates neuronal specificity of identity and race in early face-selective regions.	46	- Intranasal administration of oxytocin (OT) or placebo.<br>- 46 male participants (24 OT, 22 placebo).<br>- Presentation of face pairs with varying identities or races in rapid succession.<br>- Measurement of neural repetition suppression (RS) effects using functional magnetic resonance imaging (fMRI).<br>- RS effects used as indices of individuation/categorization face-processing.	The study provides preliminary evidence that oxytocin can regulate neuronal specificity of identity and race in early face-selective regions, enhancing adaptive individuation and categorization face-processing.	- Administration of oxytocin (OT) versus placebo<br>- Identity of face pairs (same or different)<br>- Race of face pairs (same or different)
Lu et al. (2021) [163]	- To unveil the interpersonal neural correlates that underlie the effect of group educational diversity on group creativity.	116	- Participants: 116 college students divided into high and low educational diversity groups based on academic majors.<br>- Tasks: Solved two problems, one requiring creativity (alternative uses task, AUT) and one not (object characteristics task).<br>- Technology: Functional near-infrared spectroscopy (fNIRS)-based hyperscanning to record neural responses.	High educational diversity enhances cognitive flexibility but does not necessarily improve idea quality or quantity, while low educational diversity is associated with greater fluency and perspective-taking behaviors.	- Educational diversity (high vs. low)<br>- Type of task (creativity-demanding vs. non-creativity-demanding)
Martin et al. (2019) [164]	- To examine brain stimulation differences attributable to cultural background.<br>- To investigate the effects of HD-tDCS on social cognition tasks involving self-other processing.<br>- To understand how cultural differences impact the effects of brain stimulation on social cognition.	104	- Participants: 104 young adults (52 South-East Asian Singaporeans, 52 Caucasian Australians)<br>- Intervention: Anodal high-definition transcranial direct current stimulation (HD-tDCS)<br>- Targeted brain regions: Dorsomedial prefrontal cortex (dmPFC) and right temporoparietal junction (rTPJ)	The study concludes that cultural differences in social cognition significantly influence the effects of brain stimulation, highlighting the need to consider	- Anodal high-definition transcranial direct current stimulation (HD-tDCS)<br>- Site of stimulation

			- Tasks: Visual perspective taking (VPT) and episodic memory tasks<br>- Objective: Examine cultural differences in response to brain stimulation	these differences in social brain stimulation research.	(dorsomedial prefrontal cortex (dmPFC) or right temporoparietal junction (rTPJ))<br>- Cultural background (South-East Asian Singaporeans (SEA) or Caucasian Australians (CA))
Martin et al. (2018) [165]	- Explore whether facilitation of dmPFC function by HD-tDCS can improve cross-cultural mind-reading.<br>- Replicate the cross-cultural disadvantage on the RMET by comparing Singaporean and Caucasian students.<br>- Determine if RMET performance in Singaporeans depends on their contact with Caucasians.<br>- Assess if anodal HD-tDCS to the dmPFC can remove culturally mediated disadvantage in mind-reading ability in those with less contact.	104	- Participants: 52 Singaporeans and 52 Caucasians.<br>- Design: Sham-controlled, double-blinded, crossover study.<br>- Intervention: HD-tDCS to dmPFC or rTPJ.<br>- Task: Reading the Mind in the Eyes Test (RMET).<br>- Measurement: Contact with Caucasians as a mediator.<br>- Stimulation: 1 mA for 20 minutes (active), 40 seconds (sham).<br>- Cognitive Battery: Included Stroop Test, NART, Boston Naming Test, etc.<br>- Assessments: Hospital Anxiety and Depression Scale, Autism Spectrum Quotient.<br>- Timing: Sessions at least three days apart.	The study demonstrates that electrical brain stimulation can improve cross-cultural mind-reading by targeting the dmPFC, particularly benefiting individuals with less contact with other cultures, and highlights the role of increased cultural contact in mitigating cross-cultural disadvantages.	- Stimulation Site (dmPFC or rTPJ)<br>- Contact with Caucasians
Meltzer et al. (2021) [166]	- To investigate whether the benefits of bilingualism on executive function can be replicated through deliberate intervention later in life.<br>- To compare the effects of language learning and brain training apps on executive function in older adults.	76	- Randomized clinical trial<br>- Participants: 76 adults aged 65–75<br>- Intervention duration: 16 weeks<br>- Groups: Duolingo (language learning), BrainHQ (brain training), waitlist control<br>- Daily activity: 30 minutes<br>- Assessment: Executive function tests before and after intervention<br>- Preregistered tests linked to bilingual performance	App-based language learning can improve executive function in seniors similarly to brain training, but with less impact on processing speed, and future app design improvements could enhance these benefits.	- Spanish learning using the app Duolingo<br>- Brain training using the app BrainHQ<br>- Waitlist control condition
Moon et al. (2022a) [167]	- Evaluate the impact of a multidomain lifestyle intervention on regional homogeneity (ReHo) in resting-state functional brain MRI data.<br>- Evaluate the impact of a 6-month multidomain lifestyle intervention on changes in ReHo and	152	- Participants: 152 elderly individuals without dementia, aged 60–79 years.<br>- Randomization: Participants were randomly assigned to facility-based multidomain intervention (FMI), home-based MI, or control groups.<br>- Interventions: Included monitoring and management of	Significant changes in regional spontaneous brain connectivity in at-risk elderly without substantial impairment after the FMI suggest that facility-based group preventive strategies may	- Type of intervention group (facility-based multidomain intervention, home-based multidomain intervention, control)

	ALFFs of rs-fMRI using data from the SUPERBRAIN.		metabolic/vascular risk factors, cognitive training, physical exercise, nutritional guidance, and motivational enhancement.<br>- MRI Scans: Conducted at baseline and after 24 weeks to assess changes in brain activity.<br>- Data Analysis: Used resting-state functional MRI (rs-fMRI) to measure regional homogeneity (ReHo) and amplitude of low-frequency fluctuations (ALFF).<br>- Software: Data processed using DPARSFA v5.1 and SPM 12 on Matlab 2020b.	confer cognitive benefits through neuroplastic changes of functional processing circuits in the brain areas which play a crucial role in adaptive learning and internally directed cognition.	- Time (baseline vs. post-intervention)
Moon et al. (2022b) [168]	- Evaluate the impact of a 24-week facility-based multidomain intervention (FMI) and home-based MI (HMI) on cortical thickness.<br>- Evaluate the impact on brain volume.<br>- Evaluate the impact on serum brain-derived neurotrophic factor (BDNF).	152	- 24-week intervention study with facility-based (FMI) and home-based (HMI) interventions.<br>- Participants: 152 elderly individuals with modifiable dementia risk factors, randomly assigned to FMI, HMI, or control groups.<br>- Brain MRI scans conducted at baseline and 24 weeks for 55 participants.<br>- Analysis of covariance used to compare changes in cortical thickness and gray matter volume, adjusting for age, sex, and education.<br>- ComBat site harmonization applied for standardizing measurements across scanners.<br>- Controlled for multiple comparisons with a false discovery rate threshold of $p < 0.05$.	The increase in cortical thickness and serum BDNF in the FMI group suggests that group preventive strategies at the facility may be beneficial through structural neuroplastic changes in brain areas, which facilitates learning and neurotrophic factors.	- Type of intervention (FMI, HMI, or control)
Moore et al. (2017) [169]	- Investigate the microstructural neuroplasticity effects of adding musical cues to a motor learning task.<br>- Test the hypothesis that music-cued, left-handed motor training would increase fractional anisotropy (FA) in the contralateral arcuate fasciculus.	30	- Investigated microstructural neuroplasticity effects of music-cued motor learning.<br>- Thirty right-handed participants divided into Music Group and Control Group.<br>- Training: 20 minutes, three times per week, over four weeks.<br>- Used diffusion tensor MRI and probabilistic neighbourhood tractography to measure FA, AD, and RD before and after training.	The study provides the first evidence that music-cued movement learning can induce rapid microstructural changes in white matter pathways, with potential implications for therapeutic clinical practice.	- Presence of musical cues (Music Group vs. Control Group)<br>- Training duration and frequency (20 minutes, three times per week, over four weeks)
Müller et al. (2017) [170]	- To assess whether a newly designed dance training program is superior in terms of neuroplasticity compared to conventional fitness activities.<br>- To determine if extending the training duration has additional benefits.	52	- 18-month controlled intervention study.<br>- Participants: 62 elderly individuals (63-80 years), screened and randomly assigned to dance or sport groups.<br>- Exclusion criteria: claustrophobia, tinnitus, metal implants, tattoos, diabetes, depression, cognitive deficits, neurological diseases, regular exercising.	A long-term dance program is superior to repetitive physical exercise in inducing neuroplasticity in seniors, due to its multimodal nature combining physical, cognitive,	- Type of physical activity intervention (dance training vs. conventional sport training)<br>- Duration of

	- To investigate the potential mechanisms underlying neuroplasticity by measuring BDNF levels.		- Dance group: learned new movement sequences; Sport group: performed repetitive exercises.<br>- Assessments at baseline, 6 months, and 18 months.<br>- Neuropsychological tests and fasting blood samples for BDNF levels.<br>- MRI scans analyzed with voxel-based morphometry.	and coordinative challenges, and it holds promise for preventing gray matter and cognitive decline.	intervention (6 months vs. 18 months)
Navarro-Torres et al. (2019) [171]	- Examine how real-time cognitive control engagement influences L2 sentence comprehension, focusing on conflict adaptation.<br>- Investigate whether cognitive control processes modulate how bilinguals experience syntactic ambiguity in their L2 compared to native speakers.<br>- Test the hypothesis that conflict in a non-syntactic task triggers cognitive control procedures that facilitate performance in a syntactic task.<br>- Explore how bilinguals may engage cognitive control differently due to unique demands imposed on the L2 system.	50	- Participants: 26 English monolinguals and 24 bilinguals.<br>- Method: Cross-task adaptation paradigm combining Stroop tasks with a visual-world paradigm.<br>- Tasks: Stroop task (color identification) and sentence comprehension task (following spoken instructions).<br>- Data Collection: Eye movements recorded using EyeLink eye-trackers.<br>- Analysis: Mixed effects models used to analyze fixation data.<br>- Additional Assessments: Category fluency and O-span tasks for verbal and working memory abilities.	The study found that Stroop-induced conflict facilitates recovery from syntactic ambiguity, with bilinguals showing earlier engagement in cognitive control processes compared to monolinguals, suggesting adaptive changes in cognitive control due to language experience.	- Sentence type (ambiguous vs. unambiguous)<br>- Preceding Stroop trial type (congruent vs. incongruent)<br>- Group (monolinguals vs. bilinguals)
Nestor & Woodhull (2024) [172]	- Investigate the roles of group ethnicity and display rules of emotions in the neuropsychology of social cognition in Asian American and White participants.	128	- 128 participants with a mean age of 24.9 years.<br>- Participants completed the Advanced Clinical Solutions-Social Perception (ACS-SP) for affect naming and prosody interpretation.<br>- Participants completed the Display Rule Assessment Inventory (DRAI) for emotional expressivity across different settings and domains.	The study's findings contribute to the development of culturally responsive neuropsychological models of social cognition.	- Group ethnicity<br>- Display rules of emotions
Nijmeijer et al. (2021) [173]	- Examine the effects of a foreign language training on cognitive flexibility and its neural underpinnings, and on mental health.<br>- Assess the unique role of foreign language training vs. other cognitive or social programs.	198	- Participants: 198 elderly with subjective cognitive decline.<br>- Design: Open-label randomized controlled trial with three parallel conditions.<br>- Interventions: Language training (English), music training (guitar), social control (art workshops).<br>- Duration: 3 to 6 months.<br>- Measurements: Clinical, cognitive, and brain activity assessments using EEG and fNIRS at baseline, 3 months, and 6 months post-intervention.<br>- Phases: Screening, baseline examination, intervention phase, post-intervention examination, follow-up examination.<br>- Language training: Blended learning with online activities and in-	The study is the first to explore the combined effects of language learning on cognition, language proficiency, socio-affective measures, and brain activity in elderly individuals, potentially leading to interventions that reduce cognitive decline and depression.	- Language training intervention<br>- Music training intervention<br>- Social (art) intervention

			person classes.<br>- Controls: Music intervention as high-level active control; social intervention as low-level active control.		
Nikolaidis et al. (2014) [174]	- Investigate the relationship between individual differences in training-induced changes in brain activity during a cognitive training videogame and performance changes in untrained tasks.<br>- Test whether performance changes in an untrained working memory task can be predicted by plasticity in regions associated with working memory.<br>- Extend previous literature on the association between training-related cognitive changes and changes in underlying neural networks.	70	- Participants: 45 young adults trained with a video game for 15 sessions.<br>- Assessments: Pre- and post-training neuropsychological tests on working memory, attention, and procedural learning.<br>- Imaging: Pre- and post-training fMRI scans during game play.<br>- Analysis: Correlation of brain activity with performance changes; backward multiple regression for data analysis.<br>- Recruitment: Flyers and online ads; criteria included age, gaming habits, and health status.<br>- Training: Instructional videos and practice sessions before main training.	The study demonstrates that training-induced changes in brain activity, particularly in regions associated with working memory, predict performance improvements in untrained tasks, highlighting the importance of individual differences and suggesting future research directions.	- Training with the Space Fortress video game<br>- Duration of training sessions (15 sessions of 2 hours each)<br>- Pre- and post-training neuropsychological assessments<br>- Pre- and post-training fMRI scans<br>- Instructional video provided to participants
Nilsson et al. (2018) [175]	- Investigate the effect of language training on brain structure in older adults in specific language-and memory-related gray matter regions and white matter tracts.<br>- Investigate possible predictors of achieved vocabulary proficiency in participants who completed the language training.	160	- Randomized controlled trial with two groups: language training and relaxation training.<br>- 11-week intervention with language group attending Italian classes and relaxation group participating in relaxation exercises.<br>- Structural MRI scans conducted before and after intervention to assess brain changes.<br>- Mixed factorial design with repeated-measures ANOVA to analyze data.<br>- Cognitive performance assessed with a test battery pre- and post-intervention.<br>- Structural equation modeling used to explore predictors of vocabulary learning success.	A short-term entry-level language course did not result in detectable structural changes in brain regions relevant for language and memory in older adults, but hippocampal volume and associative memory ability were robust predictors of vocabulary learning success.	- Type of intervention (language course vs. relaxation course)<br>- Time (pretest vs. posttest)
Ou et al. (2023) [176]	- To explore how in-group and out-group facial feedback impact different difficulty levels of implicit rule learning.		- Two experiments were conducted.<br>- Participants were exposed to in-group (East Asian) or out-group (Western) facial feedback.<br>- Implicit rule learning was assessed through happy and sad facial expressions.	The study concludes that highlighting group identity contrast affects implicit learning accuracy, with out-group feedback reducing accuracy in	- Type of facial feedback (in-group vs. out-group)<br>- Difficulty level of implicit rule learning

			- Experiment 2 included exposure to both in-group and out-group faces before group assignment to enhance group identity contrast.	easier tasks, which has implications for educational internationalization.	- Group identity contrast (presence or absence of both in-group and out-group faces before assignment)
Paraskevopoulos et al. (2020) [177]	- Explore aging effects on the cortical network supporting multisensory cognition.<br>- Define aging effects on the network's neuroplastic attributes.		- Development of a computer-based music reading protocol.<br>- Evaluation using electroencephalography (EEG) measurements.<br>- Pre- and post-training assessments.<br>- Comparison between young and older adults.	The study concludes that music training can induce neuroplastic changes in specific brain regions, but aging affects the strategies and extent of cortical reorganization, with older adults showing limited connectivity changes compared to younger adults.	- Age (young vs. older adults)<br>- Computer-based music reading protocol (intervention)<br>- Timing of evaluation (pre- and post-training)
Phillips et al. (2021) [178]	- To examine whether transcutaneous auricular vagus nerve stimulation (taVNS) can facilitate L2 lexical learning for English speakers learning Mandarin Chinese over 2 days.		- The study used a double-blind design.<br>- Participants were divided into three groups: continuous priming taVNS, peristimulus taVNS, and sham stimulation.<br>- Lexical recognition tests were administered to assess learning.<br>- N400 ERP components were analyzed to evaluate lexico-semantic encoding.	The study concludes that transcutaneous auricular vagus nerve stimulation (taVNS) enhances L2 lexical learning and lexico-semantic encoding, with priming taVNS particularly improving response times and being linked to sustained attentional effort.	- Type of taVNS (continuous priming taVNS, peristimulus taVNS, passive sham stimulation)<br>- Duration/timing of taVNS (10 min continuous vs. 500 msec peristimulus)
Pishghadam et al. (2024) [179]	- To address language learners' needs and facilitate language learning by modifying attention and retention processes using AVE, CES, and multisensory-based instruction.<br>- To compare the effects of AVE and CES therapies on attention and retention in L2 vocabulary learning.<br>- To highlight the efficacy of incorporating AVE and CES treatments into conventional classroom instructions and compare them with multisensory-based instruction.	32	- Participants: 32 intermediate English language learners.<br>- Design: Within-subjects design with four conditions: control (audio-visual), multisensory, AVE, and CES.<br>- Instruction: Five words taught per session using different sensory inputs.<br>- Multisensory session: Involved auditory, visual, tactile, olfactory, and gustatory inputs.<br>- Assessment: Pragmatic-Stroop task for attention; true/false test for retention.<br>- Analysis: Linear mixed-effects model for data analysis.	Multisensory instruction significantly enhances attention and L2 vocabulary retention compared to AVE and CES, as it effectively consolidates information through sensory involvement.	- Control (audio-visual instruction)<br>- Multisensory instruction (auditory, visual, tactile, olfactory, gustatory)<br>- Audio-Visual Entrainment (AVE)<br>- Cranio-Electro Stimulation (CES)

	- To discover modifications in attention and retention mechanisms using AVE, CES, and multisensory practices.				
Powers et al. (2016) [180]	- Test whether feedback indicating the need to update social knowledge engages the ventral striatum (VS) and facilitates subsequent learning.<br>- Examine the sensitivity of striatal signals to the value associated with social group membership.<br>- Test whether individual differences in desire for social acceptance modulate neural responses.	53	- Two fMRI studies were conducted to examine feedback-based learning about social groups.<br>- Participants answered questions about in-group and out-group preferences and received feedback.<br>- Experiment 1 focused on in-group knowledge; Experiment 2 compared in-group vs. out-group.<br>- Participants underwent fMRI scanning, and data were preprocessed using SPM8.<br>- A surprise memory test assessed retention of feedback post-scan.<br>- Preprocessing included noise removal, realignment, normalization, and smoothing.	The study demonstrates that the ventral striatum is sensitive to social value, with stronger learning signals for in-group members, highlighting its role in motivated learning and the importance of updating social group knowledge for sustaining harmonious intergroup relations.	- Type of feedback (correct/incorrect)<br>- Social group (in-group/out-group)
Rieker et al. (2020) [181]	- Investigate the influence of explicitly cued vs. memory-based switching conditions on the set-shifting abilities of bilingual and monolingual older adults.<br>- Investigate whether bilingualism influences age-related decline in working memory (WM).	40	- Participants: 20 bilingual and 20 monolingual older adults.<br>- Task: Task-switching task under explicit task-cuing vs. memory-based switching conditions.<br>- Conditions: Single-task, cued-switching, and memory-based switching.<br>- Duration: Each session lasted about 90 minutes.<br>- Analysis: Reaction times (RTs) for correct trials were analyzed.<br>- Inclusion Criteria: MMSE score ≥ 26, Geriatric Depression Scale score < 5, no psychiatric/neurological pathology, monolinguals with no foreign language mastery above A1 level.<br>- Bilingualism Assessment: Bilingual Language Profile questionnaire.<br>- Setup: Participants seated approximately 60 cm from the monitor.	The study suggests that bilinguals are more efficient at attention shifting in tasks mimicking dual-language management, but results should be interpreted cautiously due to small sample size.	- Group (bilingual vs. monolingual)<br>- Task type (cued vs. memory-based)<br>- Trial type (switch vs. repeat)
Ripp et al. (2022) [182]	- To evaluate potential transfer effects by comprehensive cognitive testing and neuroimaging.	55	- Prospective, randomized-controlled, single-blind study design<br>- 8-week n-back training for working memory<br>- 55 healthy middle-aged participants (50-64 years)<br>- Comprehensive cognitive testing<br>- State-of-the-art multimodal neuroimaging for anatomical and functional assessment<br>- Comparison between adaptive and non-adaptive WM training groups	Working memory training does not produce transfer effects at the cognitive or neural level, and observed practice effects may result from optimized task processing strategies rather than increased working memory capacity.	- Type of working memory training (adaptive vs. non-adaptive)

Schultz et al. (2024) [183]	- Assess behavioral and fMRI responses during a Stroop task in older adults before and after a language-learning intervention.<br>- Explore the neural effects of language learning in older adults using a pre-post intervention design with fMRI.<br>- Investigate the potential cognitive benefits of language learning, particularly in enhancing executive functions.<br>- Determine the optimal parameters for language learning as an effective cognitive intervention for aging populations.	41	- Participants: 41 older adults (age 60-80) from a monolingual environment.<br>- Intervention: Four-month online language course using Rosetta Stone, 90 minutes/day, 5 days/week.<br>- Behavioral Assessment: Pre- and post-intervention Stroop task to measure cognitive control and executive function.<br>- Neuroimaging: fMRI scans pre- and post-intervention using a 3 T Siemens Skyra scanner.<br>- Data Analysis: ANOVA for behavioral data; voxel-wise analysis for fMRI data using AFNI.	Language learning can be an effective, accessible, and enjoyable cognitive intervention for healthy aging, though further research with larger samples and control conditions is needed to confirm these benefits.	- Language-learning intervention (four-month online course)<br>- Time (pre- and post-intervention)<br>- Condition (congruent and incongruent trials in the Stroop task)
Shao et al. (2019) [184]	- To investigate whether motor skill learning (learning to play badminton) in adulthood influences resting-state activity in the cerebellum.<br>- To examine the effects of motor skill learning on resting-state functions and functional connectivity associated with the cerebellum.<br>- To assess changes in resting-state activity of specific cerebellar sub-regions (hemispheric IV-VI, VIII, and vermal VI-IX) and their intra-modular and inter-modular functional connectivity.	36	- Longitudinal design with pre- and post-training assessments.<br>- Participants: Young adults with no prior ball training experience, randomly assigned to experimental and control groups.<br>- Intervention: 12-week badminton training for the experimental group.<br>- Data collection: Resting-state fMRI scans before and after training.<br>- Data analysis: Preprocessing using DPABI V2.1 and Matlab R2012a; ALFF and whole-brain level FC analyses.<br>- Statistical analysis: Full factorial design focusing on interaction effects of test time and group.	The study found that learning to play badminton in adulthood decreases resting-state activity in certain cerebellar sub-regions while increasing functional connectivity between these sub-regions and cerebral cortices, suggesting enhanced integration of motor and cognitive functions.	- Learning to play badminton (intervention)
Sheoran et al. (2023) [185]	- Assess the effect of 12 weeks of resistance training on brain metabolism in older adults.<br>- Quantify neurometabolite ratios in specific brain regions using proton magnetic resonance spectroscopy.<br>- Assess time and group differences and examine associations between changes in peak torque and neurometabolite variables.	41	- 12-week resistance training program for older adults.<br>- Randomized control trial with experimental and control groups.<br>- Resistance training: two sessions per week, four lower body exercises, 3 sets of 6–10 reps at 70–85% of 1RM.<br>- Proton magnetic resonance spectroscopy to measure neurometabolite ratios in the hippocampus, sensorimotor, and prefrontal cortices.<br>- Peak torque of knee extension and flexion assessed using an isokinetic dynamometer.<br>- Statistical analyses: repeated measures ANOVA and correlation coefficient analyses.	Resistance training in older adults leads to beneficial alterations in neurometabolites that preserve brain health and improve muscle function.	- Type of intervention (resistance training vs. control)<br>- Frequency of training (two times weekly)<br>- Duration of training (12 weeks)<br>- Intensity of training (70–85% of 1 repetition maximum)

Simon et al. (2017) [186]	- To evaluate the efficacy of computerized cognitive training (CCT) focused on working memory (WM) compared to an active control condition in healthy older adults.	76	- Randomized controlled trial with two groups: Adaptive CCT and Non-Adaptive CCT (active control).<br>- Participants: 76 cognitively normal adults aged 65 and older from the US and Sweden.<br>- Intervention: Home-based training sessions lasting ~40 minutes, 5 days per week for 5 weeks.<br>- Adaptive CCT: Difficulty level adjusted continuously; Non-Adaptive CCT: Constant difficulty level.<br>- Baseline neuropsychological evaluation conducted.<br>- Outcome measures: Changes in age-adjusted percentile scores of Trails A and B, Digit Symbol, COWAT, and Semantic Fluency.<br>- Statistical analysis: ANOVA and ANCOVA used to assess group-by-time interactions and control for IQ.	Adaptive working memory training effectively improved executive functioning and processing speed in older adults, with benefits linked to the challenging nature of the training rather than practice effects.	- Type of computerized cognitive training (Adaptive vs. Non-Adaptive)
Sinha et al. (2020) [187]	- Investigate the effects of an aerobic exercise intervention on the dynamic rearrangement of modular community structure within the medial temporal lobe (MTL) network.<br>- Examine how MTL network flexibility mediates the effect of exercise on mnemonic flexibility, particularly in generalizing past learning to novel task demands.	34	- Used novel techniques from dynamic network neuroscience.<br>- Applied high-resolution resting-state fMRI to measure neural flexibility.<br>- Investigated effects of an aerobic exercise intervention.<br>- Employed a two-group non-randomized, repeated measures, and matched control design.<br>- Included 34 healthy older adults as participants.	The study concludes that aerobic exercise increases medial temporal lobe network flexibility, which mediates improvements in cognitive generalization by enhancing complex communication patterns within the brain.	aerobic exercise intervention
Sliwinska et al. (2021) [188]	- To test whether stimulation of the bilateral parietal region of the domain-general network impairs learning new vocabulary, indicating its causal engagement in this process.	20	- Use of repetitive transcranial magnetic stimulation (rTMS) in the form of continuous theta burst stimulation (cTBS).<br>- Participants: 20 individuals with no prior knowledge of Polish.<br>- Learning task: Polish vocabulary learning across three stages.<br>- cTBS applied to either bilateral parietal or control bilateral precentral site during the first stage.<br>- Vocabulary training immediately followed cTBS application.<br>- Different word sets for each site to control for learning effects.<br>- Vocabulary tests conducted immediately after training to assess learning and stimulation effects.<br>- Additional training without cTBS to measure effects on more established words, followed by repetition of initial procedures.	The study concludes that the bilateral parietal region of the domain-general network is crucial for successful novel word learning during the initial stages, as its stimulation impairs learning at this stage but not later.	- Application of cTBS (repetitive transcranial magnetic stimulation)<br>- Site of stimulation (bilateral parietal site vs. bilateral precentral site)<br>- Set of words used for learning

Smith et al. (2020) [189]	- Examine TLNS-related effects on the semantic N400 brain vital sign cognitive response during cognitive skills training in healthy individuals. - Investigate whether TLNS paired with cognitive skills training significantly impacts cognitive processing, as measured by brain vital signs. - Hypothesize that TLNS paired with cognitive training over 3 days would elicit N400 changes compared with cognitive training alone.	37	- Randomized controlled trial with 37 healthy volunteers. - Participants divided into treatment (TLNS) and control groups. - Cognitive training conducted twice daily for three days. - TLNS administered using the PoNS device. - Brain vital signs evaluated using EEG, focusing on N400 response. - Statistical analysis using mixed-effects linear regression.	Pairing TLNS with cognitive training led to sustained N400 amplitudes indicating increased cognitive vigilance and attention, suggesting further investigation is warranted.	- Presence or absence of TLNS (Translingual Neurostimulation)
Smith et al. (2023) [190]	- To evaluate the influence of acculturation on neuropsychological test performance in Hispanic-Americans. - To compare cognitive abilities between highly acculturated and lower acculturated Hispanic-Americans.	75	- Sample: 75 neurologically and psychologically healthy Hispanic-American undergraduate students. - Age: Mean age of 19.44 years (SD = 1.37). - Grouping: Participants divided into high (n = 39) and low (n = 36) acculturation groups. - Tools: Comprehensive neuropsychological battery and background questionnaire in English. - Acculturation Measure: Acculturation Rating Scale for Hispanic/Latino Americans (20-item scale). - Statistical Analysis: ANOVAs to evaluate cognitive differences between groups.	The study found that highly acculturated Hispanic-Americans demonstrated better language abilities, while lower acculturated individuals excelled in processing speed and executive functioning tasks, suggesting a link between language dominance and cognitive performance.	Acculturation level (high vs. low)
Struber et al. (2021) [191]	- To provide a wider understanding of adaptive learning by decoding visuomotor tasks with constant, random, or no perturbation from EEG recordings. - To separate trial-to-trial adaptation from the formation of new visuomotor mapping across trials.		- EEG recordings were used to measure brain activity. - Visuomotor tasks with constant, random, or no perturbation were employed. - A multiple kernel learning approach was used for data analysis. - Different bandwidths and brain regions were analyzed. - Tasks were designed to separate trial-to-trial adaptation from new visuomotor mapping formation.	The study concludes that there is a relationship between the modulation of low (theta) and higher (beta) frequency oscillations in prefrontal and sensorimotor regions and adaptive learning, distinguishing trial-to-trial adaptation from learning new visuomotor mappings.	- Type of perturbation in visuomotor tasks (constant, random, or no perturbation)
Wang et al. (2015) [192]	- To test the hypothesis that modifying self-construals affects in-group bias in empathy for perceived own-race vs other-race pain. - To measure neural responses to racial in-group and out-group members' suffering after priming	30	- Participants: 30 Chinese university students (16 males, 14 females; aged 18-27). - Priming: Participants were primed with independent or interdependent self-construals using essays with specific pronouns. - Imaging: Functional magnetic resonance imaging (fMRI) was used	The study demonstrates that priming independent self-construals reduces racial in-group bias in neural responses to others' pain, providing insight	- Priming (interdependent vs. independent self-construals) - Race (Asian vs.

	participants with interdependent or independent self-construals.		to measure neural responses.<br>- Design: An event-related design with four functional scans; each scan followed a priming session.<br>- Data Analysis: Functional images were preprocessed using SPM8; a general linear model was applied; ROI analyses were conducted.	into cultural differences in empathy and parochial altruism.	Caucasian models)<br>- Type of stimulus (painful vs. non-painful)
Wang et al. (2021) [193]	- To explore the BIC neural activity profiles of healthy immigrants from low-altitude regions to high-altitude regions.<br>- To determine whether long-term exposure to high altitudes affects BIC at the behavioral and neural levels.<br>- To reveal the behavioral performance and neural processing of BIC after long-term exposure to high altitudes by immigrants from sea-level regions.	71	- Two-choice oddball paradigm used to assess BIC.<br>- Practice phase with 100% accuracy required before main experiment.<br>- Two experimental blocks with standard and deviant stimuli.<br>- EEG data collected using Ag/AgCl electrodes across 64 scalp sites.<br>- Focus on N2 and P3 event-related components and theta/delta band power.<br>- Participants: 71 Han college students, with 37 in high-altitude group.<br>- Data analysis included filtering EEG waveforms and analyzing specific frequency bands.	Long-term high-altitude exposure impairs behavioral inhibitory control by affecting neural processing, leading to delayed reaction times, lower accuracy, and weaker prefrontal activity in immigrants from low-altitude regions.	- Altitude of residence (High-altitude vs. Low-altitude)
Wanrooij et al. (2014) [194]	- Examine whether the capacity for distributional learning differs between adults and infants.<br>- Directly compare adults' capacity for distributional learning to that of infants.<br>- Determine the effectiveness of distributional training of SBE /ae/,/e/ in Dutch adults.<br>- Assess if the difference in normalized MMR amplitude between bimodally and unimodally trained participants is smaller in adults than in infants.	44	- The study repeated a previous experiment conducted on infants with adult participants.<br>- Participants underwent a pre-test, training, and post-test (pre-test not done for infants).<br>- Training involved exposure to unimodal or bimodal vowel distributions.<br>- Post-test used an oddball paradigm to record MMR via EEG.<br>- EEG data were collected using a 64-channel system.<br>- Data analysis involved computing mean standard and deviant ERPs.<br>- ANOVA was used to analyze MMR amplitude differences based on distribution type and standard vowel.	Distributional training of speech sounds is less effective in adults than in infants, possibly due to weaker bottom-up learning and stronger top-down processing in adults.	- Distribution Type (unimodal vs. bimodal)<br>- Standard Vowel ([e] vs. [ae])
Wards et al. (2023) [195]	- To investigate how combined transcranial direct current stimulation and multitasking training can induce persistent gains that transfer across tasks.	178	- Combined brain stimulation and cognitive training protocols<br>- Transcranial direct current stimulation (tDCS) of the prefrontal cortex<br>- Multitasking training<br>- Functional magnetic resonance imaging (fMRI) to observe neural activity<br>- Sample size: 178 participants<br>- Stimulation parameters: 1 mA to left or right prefrontal cortex<br>- Analysis of activity changes in specific brain regions (dorsolateral prefrontal cortex, intraparietal sulcus, cerebellum)	The study concludes that functional dynamics in task-general brain regions are crucial for the transfer of training gains to untrained tasks, as demonstrated by persistent improvements in visual search performance following combined brain stimulation and multitasking training.	- Transcranial direct current stimulation (tDCS) of the prefrontal cortex<br>- Side of stimulation (left or right)<br>- Intensity of stimulation (1 mA)<br>- Multitasking training

Watson et al. (2024) [196]	- Elucidate the relationships between early life adversity (ELA), social learning, and empathic responding.<br>- Understand the impact of ELA on the expression of empathy.<br>- Understand the impact of ELA on the ability to adjust behavior after social observation.	60	- Recruitment of 60 healthy participants aged 18-65 from the Baltimore area.<br>- Use of a placebo manipulation paradigm with EEG recording to capture neural oscillations and event-related potentials.<br>- Observation of a demonstrator experiencing pain relief from an inert cream.<br>- Application of heat pain stimulations with secret reduction to measure placebo response.<br>- Participants rate their pain experience using a visual analog scale.<br>- Administration of questionnaires assessing personality, psychological factors, life history, empathy, and social life.	The study expands understanding of how early life adversity impacts behavior, highlighting neural correlates of social difficulties and empathy expression.	- Early Life Adversity (ELA)<br>- Placebo manipulation paradigm<br>- Observation of a demonstrator indicating pain relief
Worsch ech et al. (2022) [197]	- To discover if music-driven plasticity can be observed in older adult brains.<br>- To investigate the relationships between making music and morphological changes within auditory-related brain regions.<br>- To explore potential relationships between cortical thickness (CT) and monaural speech-in-noise (SIN) performance.	156	- Participants: 134 healthy, right-handed, normal-hearing, musically-naive older adults (64–76 years old).<br>- Random assignment to piano training or music listening group.<br>- Duration: 6 months of intervention.<br>- MRI scans before and after intervention using Siemens Trio and Skyra systems.<br>- Measurement: Cortical thickness (CT) and total intracranial volume (TIV) using CAT12 and SPM12 software.<br>- Regions of interest: 12 auditory-related regions as defined by the Destrieux atlas.<br>- Data analysis: Bayesian multilevel model framework using R package brms.	Playing an instrument stimulates cortical plasticity in older adults, increasing cortical thickness in specific brain areas and suggesting that musical training contributes to neuroanatomical differences between musicians and nonmusicians.	- Group (Piano training vs. Musical culture/music listening)<br>- Time (Pre vs. Post intervention)<br>- Site (Hanover vs. Geneva)<br>- Sex (Female vs. Male)<br>- Hemisphere (Left vs. Right)
Wu et al. (2021) [198]	To examine whether baseline integrity of three target white matter tract groups predicts task-switching improvement after 12-week Tai Chi Chuan training in middle-aged and older adults.	38	- Randomized controlled trial design<br>- Participants: 38 middle-aged and older adults, randomly assigned to TCC or control group<br>- Intervention: 12-week Tai Chi Chuan training, 1-hour sessions, three times a week<br>- Measurements: Cognitive task-switching and physical performance before and after training<br>- Imaging: Brain diffusion spectrum MR imaging to assess white matter integrity<br>- Analysis: General fractional anisotropy (GFA) calculated for white matter tracts<br>- Cognitive assessment: Intra-Extra Dimensional Set Shift (IED) test	The study found that Tai Chi Chuan training improves task-switching and physical functions in older adults, with pre-training integrity of the PSTP loop fiber group predicting greater cognitive improvements, highlighting the importance of prefronto-striatal circuits.	- Group assignment (TCC group vs. control group)<br>- Baseline integrity of white matter tract groups (measured by GFA)<br>- TCC training protocol (duration and frequency)

			- Data analysis: Tract-based automatic analysis and intention-to-treat approach		
Xie & Ng (2024) [199]	- Investigate how bicultural switching influences cognitive performance on Executive Functions tasks among bicultural-bilinguals.<br>- Explore the effect of cultural switching frequency on performance in tests for interference and inhibition control, set-shifting ability, and attention.	281	- Study 1:<br>- Participants: 233 young adult bilinguals<br>- Methods: Self-report questionnaires, priming experiment<br>- Analysis: Repeated measures ANOVA, two-way mixed ANOVA<br>- Study 2:<br>- Participants: 48 young adult bicultural bilinguals<br>- Methods: Tests for interference and inhibition control, set-shifting ability, and attention (Simon, Flanker, WCST)<br>- Analysis: One-way ANOVA, mixed-ANOVA for Attention Network task	The study concludes that frequent bicultural switching enhances performance on Executive Functions tasks and should be considered a significant factor in bilingual research, with broad implications for understanding multiculturalism's impact on cognition.	- Cultural switching frequency<br>- Different primes<br>- Type of interlocutor<br>- Switching Group
Xie & Antolović (2021) [200]	- To investigate whether the natural language immersion experience and the classroom intensive language training experience have differential impacts on cognitive control.		- Participants: Unbalanced Chinese-English bilingual students<br>- Groups: Natural L2 immersion group, L2 public speaking training group, control group<br>- Tasks: Flanker task, Wisconsin Card Sorting Test (WCST)<br>- Control: Demographic factors strictly controlled	The study concludes that natural L2 immersion and intensive L2 public speaking training have distinct impacts on cognitive control, supporting the Adaptive Control Hypothesis.	- Natural L2 immersion experience<br>- Classroom intensive L2 public speaking training experience
Yue et al. (2020) [201]	- Investigate whether long-term engagement in different types of physical exercise (Tai Chi Chuan and walking) influences resting-state brain networks differentially.<br>- Investigate the influence of long-term Tai Chi Chuan and walking training on resting-state functional connectivity measures to understand their effects on brain function and cognitive benefits.	42	- Cross-sectional study design.<br>- Participants: 20 older women practicing tai chi chuan and 22 older women walking regularly.<br>- Resting-state fMRI scans conducted on all participants.<br>- Data analysis using independent component analysis (ICA).<br>- Controlled for age and years of education.<br>- Imaging data collected with a 3T whole body scanner.<br>- Preprocessing and analysis using DPABI, Matlab, and GIFT software.<br>- Statistical analysis with independent sample t-tests and familywise error correction.	The study suggests that Tai Chi Chuan and walking have different effects on brain networks and functional plasticity in elderly women, with implications for preventing neurological diseases.	- Type of physical exercise (Tai Chi Chuan vs. Walking)
Zhou et al. (2022) [202]	- To investigate whether racial outgroup favoritism in neural responses to others' pain emerges during sociocultural interactions in a new social environment.		- Participants: White students divided into an experimental group (6-36 weeks in China) and a control group (2-4 weeks in China).<br>- Technique: Magnetoencephalography (MEG) was used to record neural responses.<br>- Stimuli: Pain and neutral expressions of Asian and White faces.	The study concludes that racial outgroup favoritism in neural responses to others' pain can emerge during sociocultural interactions in a new social environment.	- Duration of stay in China (6-36 weeks for Experimental group, 2-4 weeks for Control group)<br>- Type of facial

			- Focus: Neural decoding and sensorimotor responses to these expressions.		expression (pain vs. neutral)<br>- Race of the face (Asian vs. White)
Zink et al. (2019) [203]	- To examine the influence of acculturation on emotional learning using the EVLT-S.<br>- To understand how word knowledge, working memory, and acculturation contribute to performance differences between Spanish and English language groups.	77	- Participants: 50 Spanish-dominant and 27 English-dominant bilingual individuals.<br>- Tests administered: EVLT-S, LLT, Vocabulary (VC), Digit Span (DS), Short Acculturation Scale for Hispanics (SASH).<br>- Analysis: Mixed model ANOVA with test and trial as within-subjects variables and group as a between-subjects variable; repeated with VC, DS, and SASH as covariates.	The discussion suggests that differences in emotional learning and memory between Spanish and English speakers are largely due to differences in word knowledge, working memory, and acculturation, which when controlled for, eliminate group performance differences.	- Acculturation<br>- Language dominance (Spanish-dominant vs. English-dominant)<br>- Type of test (emotional vs. non-emotional)
