# Supplementary material for: Brain-Inspired Multisensory Learning: A Systematic Review of Neuroplasticity and Cognitive Outcomes in Adult Multicultural and Second Language Acquisition
Source: Biomimetics (Basel). 2025 Jun 12;10(6):397. doi: 10.3390/biomimetics10060397 (PMC12190708; doi:10.3390/biomimetics10060397)
Supplement: Supplementary file 1 [file biomimetics-10-00397-s001.zip › Sup_Table_S2_Abbreviation List.pdf]

Table S2. Abbreviation List

ACC Anterior Cingulate Cortex
ALFF Amplitude of Low-Frequency Fluctuations
ALE Activation Likelihood Estimation
ANOVA Analysis of Variance
ANT Attention Network Test
ATL Anterior Temporal Lobe
AV Audio-Visual
BDNF Brain-Derived Neurotrophic Factor
BIC Behavioral Inhibitory Control
BOLD Blood Oxygen Level-Dependent
CA1 Cornu Ammonis 1 (hippocampal subfield)
CCT Computerized Cognitive Training
CES Cranio-Electro Stimulation
COMT Catechol-O-Methyltransferase
CT Cortical Thickness
cTBS Continuous Theta Burst Stimulation
DBT Dynamic Balancing Task
DCM Dynamic Causal Modeling
DLPFC Dorsolateral Prefrontal Cortex
DMN Default Mode Network
DRAI Display Rule Assessment Inventory
DTI Diffusion Tensor Imaging
DWI Diffusion-Weighted Imaging
ECoG Electrocorticography
EEG Electroencephalography
ELA Early Life Adversity
ERP Event-Related Potential
EVLT-S Emotional Verbal Learning Test-Spanish
FA Fractional Anisotropy
FDG-PET Fluorodeoxyglucose Positron Emission Tomography
FLAIR Fluid Attenuated Inversion Recovery
fMRI Functional Magnetic Resonance Imaging
fNIRS Functional Near-Infrared Spectroscopy
GFA General Fractional Anisotropy
GMV Grey Matter Volume
HAWIE-R Hamburg-Wechsler Intelligence Test for Adults-Revised
HD-tDCS High-Definition Transcranial Direct Current Stimulation
IAT Implicit Association Test
ICA Independent Component Analysis
ICN Intrinsic Connectivity Network
IED Intra-Extra Dimensional Set Shift
IFG Inferior Frontal Gyrus
Ins Insula
L1 First Language
L1A First Language Acquisition
L2 Second Language
L2A Second Language Acquisition
LLT Language Learning Task
MDPI Multidisciplinary Digital Publishing Institute
MEG Magnetoencephalography
mPFC Medial Prefrontal Cortex
MMN Mismatch Negativity
MMR Mismatch Response
MPRAGE Magnetization Prepared Rapid Acquisition Gradient Echo
MRI Magnetic Resonance Imaging
MRS Magnetic Resonance Spectroscopy
MTCC Multicomponent Training of Cognitive Control

MVPA Multivariate Pattern Analysis
NART National Adult Reading Test
NCGG-FAT National Center for Geriatrics and Gerontology-Functional Assessment Tool
NODDI Neurite Orientation Dispersion and Density Imaging
OXT/OT Oxytocin
PANAS Positive and Negative Affect Schedule
phMRI Pharmacological Magnetic Resonance Imaging
PLC Placebo
PoNS Portable Neuromodulation Stimulator
PPI Psychophysiological Interaction
PSTP Prefronto-Striato-Thalamo-Prefrontal
RALT Reinforcement Association Learning Task
RBANS Repeatable Battery for the Assessment of Neuropsychological Status
RD Radial Diffusivity
ReHo Regional Homogeneity
REM Rapid Eye Movement
RMET Reading the Mind in the Eyes Test
ROI Region of Interest
RPE Rating of Perceived Exertion
rs-fMRI Resting-State Functional Magnetic Resonance Imaging
RS Repetition Suppression
SASH Short Acculturation Scale for Hispanics
sEEG Stereo-Electroencephalography
SENAS Spanish and English Neuropsychological Assessment Scales
SES Socioeconomic Status
SIN Speech-in-Noise
SLFII Superior Longitudinal Fasciculus II
SPM Statistical Parametric Mapping
STG Superior Temporal Gyrus
SUPERBRAIN Controlling Vascular and Lifestyle Risk Factors for Alzheimer's Disease
taVNS Transcutaneous Auricular Vagus Nerve Stimulation
TCC Tai Chi Chuan
tDCS Transcranial Direct Current Stimulation
TEI Trait Emotional Intelligence
TLNS Translingual Neurostimulation
TIV Total Intracranial Volume
TMS Transcranial Magnetic Stimulation
TNF-α Tumor Necrosis Factor Alpha
TPJ Temporoparietal Junction
tRNS Transcranial Random Noise Stimulation
VBM Voxel-Based Morphometry
VE Virtual Environment
VO2 AT Oxygen Consumption at Anaerobic Threshold
VO2 max Maximal Oxygen Consumption
VPT Visual Perspective Taking
VR Virtual Reality
VS Ventral Striatum
WCST Wisconsin Card Sorting Test
WM Working Memory
μFA Microscopic Fractional Anisotropy
